# Synthesis and Antiprotozoal Activity of Azabicyclo-Nonane Pyrimidine Hybrids

**DOI:** 10.3390/molecules28010307

**Published:** 2022-12-30

**Authors:** Clemens Hinteregger, Johanna Dolensky, Werner Seebacher, Robert Saf, Pascal Mäser, Marcel Kaiser, Robert Weis

**Affiliations:** 1 Pharmaceutical Chemistry, Institute of Pharmaceutical Sciences, University of Graz, Schubertstraße 1, A-8010 Graz, Austria; 2 Institute for Chemistry and Technology of Materials (ICTM), Graz University of Technology, Stremayrgasse 9, A-8010 Graz, Austria; 3 Swiss Tropical and Public Health Institute, Kreuzstraße 2, CH-4123 Allschwil, Switzerland; 4 Swiss Tropical and Public Health Institute, University of Basel, Petersplatz 1, CH-4003 Basel, Switzerland

**Keywords:** azabicyclo-nonanes, hybrids, *Plasmodium falciparum*, pyrimidine, *Trypanosoma brucei rhodesiense*

## Abstract

2,4-Diaminopyrimidines and (dialkylamino)azabicyclo-nonanes possess activity against protozoan parasites. A series of fused hybrids were synthesized and tested in vitro against pathogens of malaria tropica and sleeping sickness. The activities and selectivities of compounds strongly depended on the substitution pattern of both ring systems as well as on the position of the nitrogen atom in the bicycles. The most promising hybrids of 3-azabicyclo-nonane with 2-aminopyrimidine showed activity against *P. falciparum* NF54 in submicromolar concentration and high selectivity. A hybrid with pyrrolidino substitution of the 2-azabicyclo-nonane as well as of the pyrimidine moiety exhibited promising activity against the multiresistant K1 strain of *P. falciparum*. A couple of hybrids of 2-azabicyclo-nonanes with 2-(dialkylamino)pyrimidines possessed high activity against *Trypanosoma brucei rhodesiense* STIB900 and good selectivity.

## 1. Introduction

Malaria and human African trypanosomiasis (HAT, sleeping sickness) are both tropical diseases transmitted by the bite of infected insects. Malaria is caused by *Plasmodium* parasites. In 2020 there were about 241 million estimated cases of malaria and about 627,000 reported deaths [1]. There are five types of human malaria parasites, but the majority of infections are caused by *Plasmodium falciparum*, the deadliest malaria parasite. A lot of strains of *Plasmodium falciparum* have become resistant to previous generations of medicines [1]. In recent years even resistance to recommended artemisinin-based therapies has become prevalent across an expanding area of Southeast Asia [2,3]. There is growing evidence that resistant strains have reached regions in Africa [1]. Therefore, there is still a necessity to develop new compounds with distinct activity against *Plasmodium falciparum*.

Human African trypanosomiasis is caused by *Trypanosoma brucei* parasites. After continued control efforts the number of reported cases dropped below 1000 in 2019. However, as history has shown it may re-emerge and the estimated population at risk is 65 million people. In case of *T. b. gambiense* infections, the disease is chronic, generally lasting several years without any major signs or symptoms. However, infections with *T. b. rhodesiense* cause the acute form of the disease, lasting from a few weeks to several months and are lethal if untreated [4]. This East African sleeping sickness is difficult to treat considering the toxicity and complex administration of the drugs currently in use. For the treatment of *T. b. rhodesiense* infections of the central nervous system, melarsoprol is the only effective drug. Unfortunately, this arsenic-containing medication may cause encephalopathy, killing one in twenty patients [5]. Therefore, the development of new drugs against the East African form of HAT is urgently required. 

**Figure 1:**
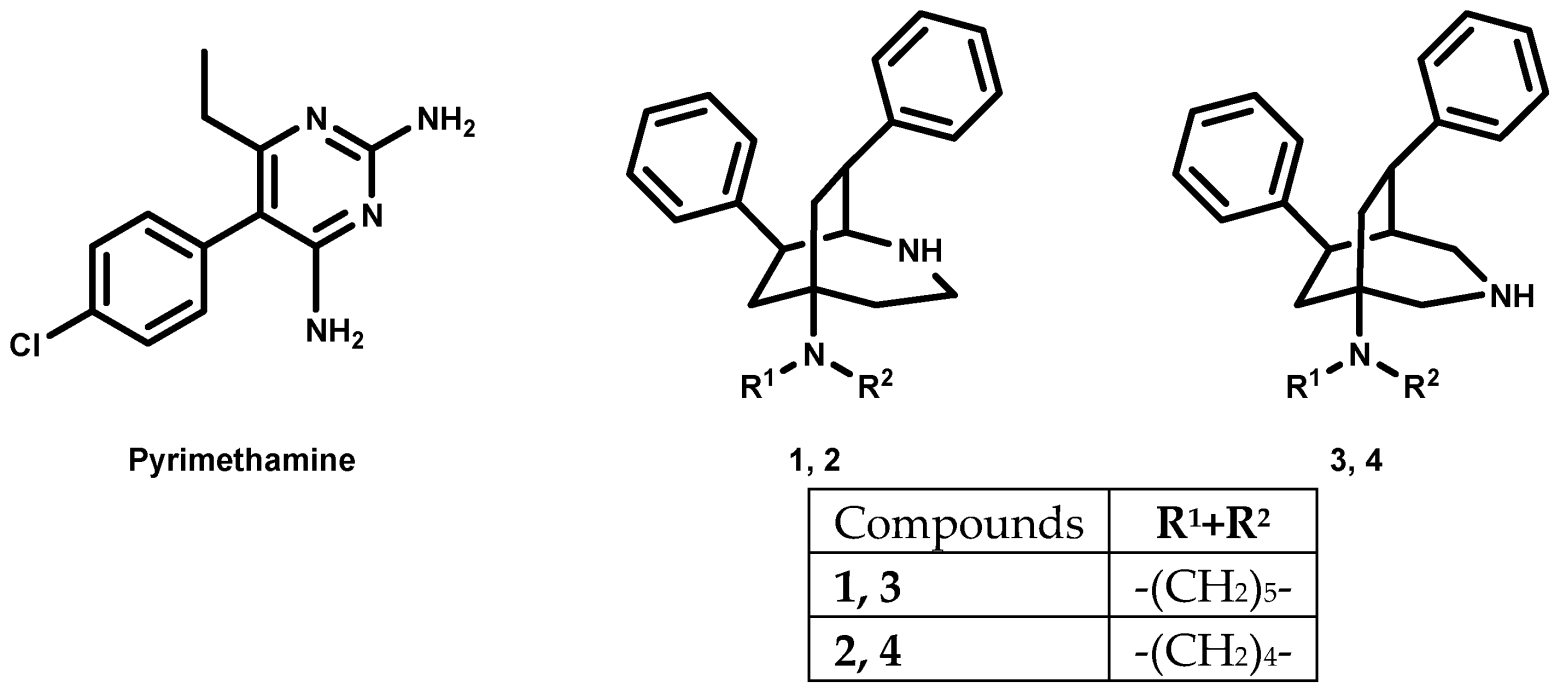
Pyrimethamine, 2-azabicyclo[3.2.2]nonanes and 3-azabicyclo[3.2.2]nonanes.

An important drug in the therapy against protozoan parasites (Figure 1) is 2,4-diaminopyrimidine pyrimethamine. It targets the enzymes of the folate metabolism, and so, in the case of malaria it selectively inhibits the plasmodial form of dihydrofolate reductase and reduces the production of folic acid required for nucleic acid synthesis in the *Plasmodium* parasite [6]. It is effective against the erythrocytic stages of all *Plasmodium spp*. that are pathogenic for humans, but resistance has significantly limited its usefulness as a single agent [7]. As a consequence, it is used for treatment of acute malaria only in combination with other drugs to prevent resistance. In addition, pyrimethamine is also used for the development of novel antiparasitic treatments [8,9,10]. Trypanosomes are unable to synthesize folates and pterins, which they need for nucleic acid synthesis. They use the extracellular pterin precursors of their hosts. These have to be modified by two reductions to yield active tetrahydro-derivates. Pyrimethamine was found to selectively inhibit the trypanosomal form of DHFR-thymidylate synthase (DHFR-TS) and pteridine reductase 1 (PTR1), and in this way, the production of folic acid required by trypanosomes [11].

The 2-azabicyclo[3.2.2]nonanes **1, 2** and the 3-azabicyclo[3.2.2]nonane **3, 4** show antiplasmodial activity in submicromolar concentration. Their antitrypanosomal activity requires low micromolar concentrations [12,13].

In drug development against plasmodia the combination of different pharmacophores in one hybrid molecule is a potential option to bypass drug resistance, to improve pharmacokinetics or to achieve multistage antimalarial activity [14]. Recently, we reported about the impact of the linkage of tetrazole or sulfonamide cores to antiprotozoal active (dialkylamino)azabicyclo-nonanes [15]. A series of new quinoline-pyrimidine hybrids and their biological activities have been published in the last decade [16,17,18,19,20]. This paper presents the preparation of fused hybrids of (dialkylamino)azabicyclo-nonanes with the 2,4-diaminopyrimidine pharmacophore of pyrimethamine and selected analogues.

## 2. Results

### 2.1. Chemistry

The 2-azabicyclo[3.2.2]nonanes **1**, **2** were prepared from bicyclo[2.2.2]octan-2-ones [21] via a Beckmann rearrangement and the subsequent reduction of the lactam intermediates [22]. Alternatively, bicyclo[2.2.2]octan-2-ones were converted to lactams via a Schmidt reaction. Their reduction yielded 3-azabicyclo[2.2.2]nonanes **3**, **4** [12,23]. A scheme is given in the Supplemental Materials.

**Scheme 1:**
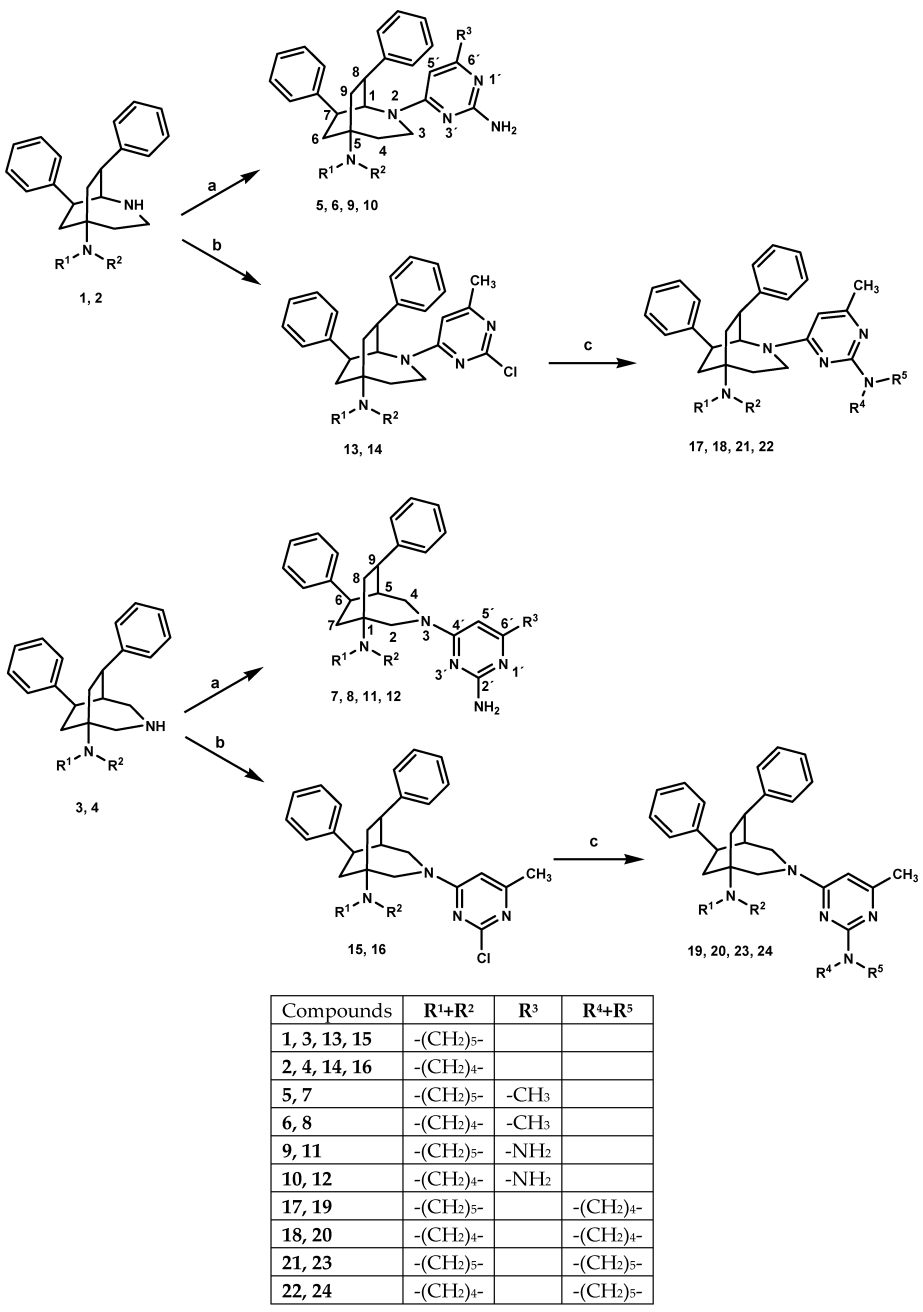
Preparation of compounds **5**–**24**. Reagents and conditions: (a) corresp. 4-chloropyrimidine, DIPEA, 1-BuOH, 145 °C, 48h; (b) 2,4-dichloro-6-methylpyrimidine, DIPEA, EtOH, 0 °C→RT, 44–188 h; (c) sec. amine, DIPEA, ACN, 105 °C, 20–48h.

The azabicyclo-nonanes **1**–**4** were refluxed with the corresponding 4-chloropyrimidine in boiling butan-1-ol for 2 d giving pyrimidine hybrids **5**–**12**. DIPEA served as a proton catcher. Regioselective reactions with 2,4-dichloro-6-methylpyrimidine yielding **13**–**16** required milder conditions. The experiments were carried out at room temperature and were more time-consuming, accordingly. The second chloro substituent was finally replaced by reaction with excess secondary amine in boiling acetonitrile giving **17**–**24**. (Scheme 1)

The structures of all newly synthesized compounds were clarified by one- and two-dimensional NMR spectroscopy. Successful N-arylation was detected by typical shifts of resonances and remarkable signal broadening, particularly of the bicyclic ring atoms in the vicinity of the new bonding sites. The most striking change was a 17–19 ppm upfield shift of C-5′ of the pyrimidine moiety due to the mesomeric effect of the new nitrogen substituent. In consequence of restricted rotation, the signals of each of the two protons H-2 and H-4 of the 3-azabicyclo-nonanes were strongly broadened. The corresponding signals of H-1 and one of the two H-3 protons of 2-azabicyclo-nonanes were even invisible in the ^1^H NMR spectrum. The signals of the adjacent ring carbon atoms of both azabicyclo-nonanes were massively broadened and typically shifted about 1.5–5.5 ppm to lower frequencies. Their chemical shifts were usually deduced from 2D spectra, and ^1^H- and ^13^C-nmr spectra of the new compounds are given in the supplementary material.

### 2.2. Antiprotozoal Activity and Cytotoxicity

Compounds were tested for their activities against *Plasmodium falciparum* NF54 (sensitive to chloroquine) and *Trypanosoma brucei rhodesiense* STIB900, via serial dilution assays. Furthermore, selected compounds were tested against the K1 strain of *Plasmodium falciparum* (resistant against chloroquine, sulfadoxine and pyrimethamine). Their cytotoxicity was determined with rat skeletal myoblasts (L-6 cells). Chloroquine, melarsoprol and podophyllotoxin served as standards; additional data for pyrimethamine are included (Table 1).

## 3. Discussion

**Figure 2:**
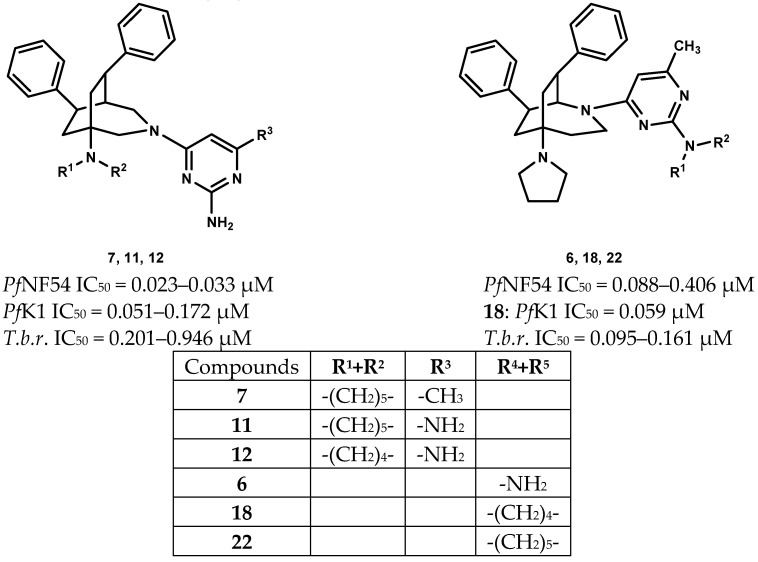
Most active compounds.

Many of the newly prepared compounds showed activity against *P.falciparum* NF54 (*Pf*NF54 IC_50_ = 0.023–0.694 µM) in submicromolar concentration (Table 1). Their activity was highly influenced by the substituents of the pyrimidine scaffold. Compounds **6**–**12** (*Pf*NF54 IC_50_ = 0.023–0.088 µM) with a 2,6-diamino or a 2-amino-6-methyl substitution were the most active ones. Within this group the 3-azabicyclo-nonanes (**7**, **11**, **12** (*Pf*NF54 IC_50_ = 0.023–0.033 µM) were more active than their 2-azabicyclo-nonane analogues **5**, **9**, **10** (*Pf*NF54 IC_50_ = 0.051–0.212 µM) (Figure 2). They showed roughly the same activity as pyrimethamine (*Pf*NF54 IC_50_ = 0.017 µM). The parent *N*-unsubstituted 3-azabicyclo-nonane **4** was less active (*Pf*NF54 IC_50_ = 0.072 µM) but highly selective (SI = 1013). In comparison with their pyrrolidino analogues, compounds with piperidino substitution of the bridgehead atom showed higher activity. With the exception of **8** (SI = 72.3), their selectivity indices were very good due to their low cytotoxicity (**6**, **7**, **9**–**12**: SI = 142.5–337.3). Compounds with a 2-dialkylamino- **17**–**24** (*Pf*NF54 IC_50_ = 0.087–5.29 µM) or a 2-chloropyrimidine moiety **13**–**16** (*Pf*NF54 IC_50_ = 0.281–1.49 µM) were less active and less selective (SI = 4.47–110.7).

In addition, selected compounds were tested against the multiresistant K1 strain of *Plasmodium falciparum*. Pyrimethamine was inactive against this strain (*Pf*K1 IC_50_ = 10.00 µM). In comparison to the activities against the NF54 strain, the observed activities were lower for the 2,6-diamino or 2-amino-6-methyl substituted compounds **7** and **9**–**12** (*Pf*K1 IC_50_ = 0.051–0.216 µM). However, compounds **5**–**7** and **9**–**11** were more active than their parent azabicyclo-nonanes **1**–**3**. Compounds **7** and **9** exhibited good activity (*Pf*K1 IC_50_ = 0.051–0.094 µM) and selectivity (SI = 103.6–155.6). In contrast, compounds with a 2-dialkylamino or a 2-chloropyrimidine moiety were by trend more active against the K1 than against the NF54 strain. Highest activities were observed for the bis(pyrrolidino) substituted compounds **18** and **20**. The 3-azabicyclo-nonane **20** showed equal activity against both strains (*Pf*K1 IC_50_ = 0.087 µM). Its activity was comparable to its parent 3-azabicyclo-nonane **4** (*Pf*K1 IC_50_ = 0.095 µM), but the selectivity was considerably lower. Compared to its parent 2-azabicyclo-nonane **2** (*Pf*K1 IC_50_ = 0.56 µM; SI = 215.0), compound **18** showed improved activity (*Pf*K1 IC_50_ = 0.059 µM) and selectivity (SI = 254.5). A further promising issue is its increased activity against the multiresistant strain.

The antitrypanosomal activity of the parent azabicyclo-nonanes **1**–**4** was moderate (*T.b.r*. IC_50_ = 1.00–6.57 µM). Their activity was changed by the substitution with the pyrimidine substituent, but the impact of the substitution of the pyrimidine ring was varying. Compounds with a 2-azabicyclo-nonane skeleton were in general more active than their 3-azabicyclo-nonane analogues, and pyrrolidino substitution of the bridgehead atom furnished higher activity compared to piperidino substitution. The 2-(6-methylpyrimidin-4-yl)-5-pyrrolidino-2-azabicyclo[3.2.2]nonanes **6**, **18**, **22** showed the highest activity (*T.b.r*. IC_50_ = 0.095–0.161 µM) and selectivity (SI = 62.90–158.4). All the other test compounds were by far less selective (SI ≤ 42.63). (Table 1)

The molecular targets and the mechanisms of the antiplasmodial and the antitrypanosomal action of azabicyclo-nonanes are not yet known. The interpretation of test results relies on phenotypic screening, considering that inhibitors act against their antitrypanosomal targets in approximately physiological circumstances.

## 4. Materials and Methods

### 4.1. Instrumentation and Chemicals

IR spectra were recorded using a Bruker Alpha Platinum ATR FTIR spectrometer; frequencies are reported in cm^−1^. NMR spectra: Varian Unity Inova 400 (298 K) 5 mm tubes, TMS as internal standard; ^1^H NMR (400 MHz) and ^13^C NMR (100 MHz) spectra are reported in ppm; ^1^H- and ^13^C-resonances were assigned using ^1^H,^1^H-; and ^1^H,^13^C-correlation spectra and are numbered as given in the formulas. Signal multiplicities are abbreviated as follows: br broad, d doublet, dd doublet of doublets, ddd doublet of doublet of doublets, m multiplet, s singlet, t triplet, td triplet of doublets; resonances marked with a single quote belong to the pyrimidine substituent of the compounds (Scheme 1). HRMS: GCT-Premier, Waters (EI, 70 eV). Materials: column chromatography (CC): aluminium oxide 90 (neutral, Merck, Rahway, NJ, USA), aluminium oxide 60 (pH: 9.5, Merck), silica gel 60 (Merck 70–230 mesh, pore-diameter 60 Å), thin-layer chromatography (TLC): TLC plates silica gel 60 F254 (Merck), aluminium oxide 60 F254 (neutral, Merck).

### 4.2. Syntheses

The syntheses of compounds **1**–**4** have already been reported elsewhere [12,22,23].

#### 4.2.1. General Procedure for the Syntheses of **5**–**12**

The corresponding azabicyclo[3.2.2]nonane **1**, **2**, **3**, **4**, the 4-chloro-6-methylpyrimidin-2-amine, respectively, 6-chloropyrimidine-2,4-diamine and DIPEA were suspended in butan-1-ol. The reaction batch was refluxed for 48h at 145 °C in an atmosphere of Ar. After cooling to room temperature, the mixture was diluted with diethyl ether or CH_2_Cl_2_ and alkalized with 2*N* NaOH. The combined organic phases were washed with water, dried over anhydrous sodium sulfate, filtered and finally the solvent was removed in vacuo giving crude products which were purified by column chromatography.

*rac*-4-[(7*R*,8*R*)-7,8-diphenyl-5-(piperidin-1-yl)-2-azabicyclo[3.2.2]nonan-2-yl]-6-methylpyrimidin-2-amine **5**

The reaction of 1.3g 5-(piperidin-1-yl)-2-azabicyclo-nonane **1** (3.61 mmol), 0.535 g 4-chloro-6-methylpyrimidin-2-amine (3.73 mmol) and 0.982 g DIPEA (7.60 mmol) in 50 mL butan-1-ol gave after 48 h a crude product which was purified by column chromatography (silica, CH_2_Cl_2_ + MeOH = 19 + 1) yielding 0.715 g **5** (42%, 1.53 mmol) as amorphous solid. IR = 2929, 1584, 1543, 1448, 1416, 1255, 1103, 792, 698; ^1^H NMR (CDCl_3_) δ = 1.42–1.50 (m, 2H, CH_2_), 1.56–1.68 (m, 4H, 2CH_2_), 1.83 (br, td, *J* = 13.5, 4.6 Hz, 1H, 4-H), 1.91–2.02 (m, 2H, 4-H, 6-H), 2.10–2.19 (m, 1H, 9-H), 2.16 (s, 3H, CH_3_), 2.33–2.44 (m, 2H, 6-H, 9-H), 2.51–2.68 (m, 4H, N(CH_2_)_2_), 3.02–3.11 (m, 1H, 3-H), 3.22 (t, *J* = 9.6 Hz, 1H, 7-H), 3.43–3.52 (m, 1H, 8-H), 4.68 (s, 2H, NH_2_), 5.71 (br, s, 1H, 5’-H), 7.09–7.51 (m, 10H, aromatic H); ^13^C NMR (CDCl_3_) δ = 24.06 (CH_3_), 24.99 (CH_2_), 26.73 (2CH_2_), 31.37 (C-4), 36.26 (C-6), 36.60 (C-9), 37.08 (C-8), 41.17 (br, C-3), 46.05 (C-7), 46.30 (N(CH_2_)_2_), 56.56 (br, C-1), 58.32 (C-5), 93.30 (C-5′), 126.22, 126.61, 126.79, 127.54, 128.41, 128.59 (aromatic C), 143.47, 144.35 (aromatic C_q_), 162.31 (C-4′), 162.52 (C-2′), 165.94 (C-6′); HRMS (EI+) calcd for C_30_H_37_N_5_: 467.3049; found: 467.3050.

*rac*-4-[(7*R*,8*R*)-7,8-diphenyl-5-(pyrrolidin-1-yl)-2-azabicyclo[3.2.2]nonan-2-yl]-6-methylpyrimidin-2-amine **6**.

The reaction of 1.32 g 5-(pyrrolidin-1-yl)-2-azabicyclononane **2** (3.81 mmol), 0.547 g 4-chloro-6-methylpyrimidin-2-amine (3.81 mmol) and 0.985 g DIPEA (7.62 mmol) in 50 mL butan-1-ol gave after 48 h 1.54 g **6** (89%, 3.39 mmol) as amorphous solid. IR = 3087, 2952, 2872, 2807, 1639, 1586, 1541, 1497, 1485, 1448, 1417, 1355, 1254, 1213, 1175, 1122, 1032, 1011, 966, 934, 881, 790, 750, 698; ^1^H NMR (CDCl_3_) δ = 1.76–1.82 (m, 4H, (CH_2_)_2_), 1.92 (br, td, *J* = 14.2, 4.2 Hz, 1H, 4-H), 1.93–1.99 (m, 1H, 4-H), 2.07–2.20 (m, 2H, 6-H, 9-H), 2.17 (s, 3H, CH_3_), 2.30–2.37 (m, 1H, 6-H), 2.49 (t, *J* = 12.3 Hz, 1H, 9-H), 2.69–2.79 (m, 4H, N(CH_2_)_2_), 3.09 (ddd, *J* = 14.6, 11.7, 4.5 Hz, 1H, 3-H), 3.28 (t, *J* = 9.5 Hz, 1H, 7-H), 3.48–3.56 (m, 1H, 8-H), 4.69 (s, 2H, NH_2_), 5.73 (br, s, 1H, 5′-H), 7.11-7.50 (m, 10H, aromatic H); ^13^C NMR (CDCl_3_) δ = 23.54 ((CH_2_)_2_), 24.08 (CH_3_), 31.63 (C-4), 36.38 (C-8), 37.16 (C-9), 37.38 (C-6), 40.55 (br, C-3), 45.21 (N(CH_2_)_2_), 45.71 (C-7), 56.87 (C-1, C-5), 93.28 (C-5′), 126.20, 126.63, 126.74, 127.62, 128.40, 128.62 (aromatic C), 143.42, 144.33 (aromatic C_q_), 162.37 (C-4′), 162.50 (C-2′), 166.02 (C-6′); HRMS (EI+) calcd for C_29_H_35_N_5_: 453.2892; found: 453.2914.

*rac*-4-[(7*R*,8*R*)-7,8-diphenyl-1-(piperidin-1-yl)-3-azabicyclo[3.2.2]nonan-3-yl]-6-methylpyrimidin-2-amine **7**.

The reaction of 1.17 g 1-(piperidin-1-yl)-3-azabicyclononane **3** (3.25 mmol), 0.480 g 4-chloro-6-methylpyrimidin-2-amine (3.34 mmol) and 0.947 g DIPEA (7.33 mmol) in 45 mL butan-1-ol gave after 48 h a product of 1.35 g **7** (89%, 2.89 mmol) as amorphous solid. IR = 3153, 3027, 2927, 1585, 1494, 1453, 1422, 1307, 1252, 1221, 1182, 1152, 1104, 1011, 963, 792, 755, 733, 700; ^1^H NMR (CDCl_3_) δ = 1.45–1.52 (m, 2H, CH_2_), 1.58–1.66 (m, 4H, 2CH_2_), 1.90 (dd, *J* = 13.0, 9.8 Hz, 1H, 8-H), 2.00 (t, *J* = 11.8 Hz, 1H, 7-H), 2.09–2.17 (m, 2H, 5-H, 8-H), 2.22 (s, 3H, CH_3_), 2.24–2.33 (m, 1H, 7-H), 2.57–2.65 (m, 2H, 2NCH), 2.81–2.90 (m, 2H, 2NCH), 3.08 (d, *J* = 12.8 Hz, 1H, 4-H), 3.16 (d, *J* = 14.6 Hz, 1H, 2-H), 3.35–3.43 (m, 2H, 6-H, 9-H), 3.77–3.85 (m, 1H, 4-H), 4.73 (s, 2H, NH_2_), 5.04-5.14 (br, 1H, 2-H), 5.75 (s, 1H, 5′-H), 7.07 (d, *J* = 7.0 Hz, 2H, aromatic H), 7.15–7.43 (m, 8H, aromatic H); ^13^C NMR (CDCl_3_) δ = 24.12 (CH_3_), 25.06 (CH_2_), 26.82 (2CH_2_), 30.78 (C-8), 38.19 (C-7), 39.07 (C-9), 43.78 (C-6), 44.91 (C-5), 46.89 (N(CH_2_)_2_), 49.14 (C-2), 50.37 (C-4), 60.07 (C-1), 93.20 (C-5′), 126.36, 126.44, 126.68, 128.18, 128.38, 128.77 (aromatic C), 143.46, 146.12 (aromatic C_q_), 162.16 (C-2′), 163.80 (C-4′), 166.25 (C-6′); HRMS (EI+) calcd for C_30_H_37_N_5_: 467.3049; found: 467.3051.

*rac-*4-[(7*R*,8*R*)-7,8-diphenyl-1-(pyrrolidin-1-yl)-3-azabicyclo[3.2.2]nonan-3-yl]-6-methylpyrimidin-2-amine **8**.

The reaction of 0.935 g 1-(pyrrolidin-1-yl)-3-azabicyclononane **4** (2.70 mmol), 0.410 g 4-chloro-6-methylpyrimidin-2-amine (2.86 mmol) and 0.750 g DIPEA (5.80 mmol) in 45 mL butan-1-ol gave after 48 h a crude product which was purified by column chromatography (silica, CH_2_Cl_2_ + MeOH = 19 + 1) yielding 1.00 g **8** (82%, 2.21 mmol) as amorphous solid. IR = 2960, 1583, 1494, 1450, 1419, 1308, 1252, 1219, 1180, 1155, 1032, 965, 792, 753, 699; ^1^H NMR (CDCl_3_) δ = 1.78–1.84 (m, 4H, (CH_2_)_2_), 1.96 (dd, *J* = 12.8, 9.9 Hz, 1H, 8-H), 2.06–2.15 (m, 2H, 5-H, 7-H), 2.18–2.25 (m, 1H, 8-H), 2.21 (s, 3H, CH_3_), 2.36 (ddd, *J* = 13.0, 8.4, 1.7 Hz, 1H, 7-H), 2.74–2.82 (m, 2H, 2NCH), 2.91–2.99 (m, 2H, 2NCH), 3.14 (br, d, *J* = 13.2 Hz, 1H, 4-H), 3.26 (d, *J* = 13.9 Hz, 1H, 2-H), 3.38–3.47 (m, 2H, 6-H, 9-H), 3.77–3.86 (m, 1H, 4-H), 4.73 (s, 2H, NCH_2_), 5.07 (br, 1H, 2-H), 5.75 (s, 1H, 5′-H), 7.07 (d, *J* = 7.0 Hz, 2H, aromatic H), 7.15–7.45 (m, 8H, aromatic H); ^13^C NMR (CDCl_3_) δ = 23.62 ((CH_2_)_2_), 24.12 (CH_3_), 31.81 (C-8), 38.71 (C-9), 38.82 (C-7), 43.74 (C-6), 45.36 (C-5), 45.70 (N(CH_2_)_2_), 49.16 (C-2), 50.35 (C-4), 58.32 (C-1), 93.23 (C-5′), 126.35, 126.46, 126.76, 128.15, 128.34, 128.77 (aromatic C), 143.38, 146.05 (aromatic C_q_), 162.19 (C-2′), 163.77 (C-4′), 166.24 (C-6′); HRMS (EI+) calcd for C_29_H_35_N_5_: 453.2892; found: 453.2894.

*rac*-6-[(7*R*,8*R*)-7,8-diphenyl-5-(piperidin-1-yl)-2-azabicyclo[3.2.2]nonan-2-yl]pyrimidine-2,4-diamine **9**.

The reaction of 0.160 g 5-(piperidin-1-yl)-2-azabicyclononane **1** (0.44 mmol), 0.098 g 6-chloropyrimidine-2,4-diamine (0.68 mmol) and 0.129 g DIPEA (0.10 mmol) in 7 mL butan-1-ol gave after 48 h a crude product which was purified by column chromatography (silica, CH_2_Cl_2_ + MeOH = 19 + 1) yielding 0.027 g **9** (14%, 0.06 mmol) as amorphous solid. IR = 3397, 2929, 1579, 1423, 1240, 1103, 1032, 793, 699; ^1^H NMR (CDCl_3_) δ = 1.46–1.53 (m, 2H, CH_2_), 1.65–1.76 (m, 4H, 2CH_2_), 1.88 (td, *J* = 13.5, 4.0 Hz, 1H, 4-H), 1.98 (br, d, *J* = 13.5 Hz, 1H, 4-H), 2.03–2.19 (m, 2H, 6-H, 9-H), 2.39 (ddd, *J* = 12.4, 8.9, 2.6 Hz, 1H, 6-H), 2.52 (t, *J* = 12.0 Hz, 1H, 9-H), 2.58–2.79 (m, 4H, N(CH_2_)_2_), 2.98–3.08 (m, 1H, 3-H), 3.25 (t, *J* = 9.5 Hz, 1H, 7-H), 3.45-3.54 (m, 1H, 8-H), 4.31 (br, s, 2H, NH_2_), 4.52 (s, 2H, NH_2_), 5.00 (s, 1H, 5′-H), 7.13–7.36 (m, 8H, aromatic H), 7.48 (d, *J* = 7.5 Hz, 2H, aromatic H); ^13^C NMR (CDCl_3_) δ = 24.66 (CH_2_), 26.14 (2CH_2_), 31.62 (C-4), 36.13 (C-6), 36.35 (C-9), 37.09 (C-8), 40.53 (C-3), 45.90 (C-7), 46.44 (N(CH_2_)_2_), 56.51 (C-1), 59.79 (C-5), 75.32 (C-5′), 126.30, 126.65, 126.84, 127.53, 128.48, 128.69 (aromatic C), 143.40, 144.29 (aromatic C_q_), 162.11 (C-6′), 163.15 (C-4′), 164.17 (C-2′); HRMS (EI+) calcd for C_29_H_36_N_6_: 468.3001; found: 468.2985.

*rac*-6-[(7*R*,8*R*)-7,8-diphenyl-5-(pyrrolidin-1-yl)-2-azabicyclo[3.2.2]nonan-2-yl]pyrimidine-2,4-diamine **10**.

The reaction of 0.350 g 5-(pyrrolidin-1-yl)-2-azabicyclononane **2** (2.70 mmol), 0.172 g 6-chloropyrimidine-2,4-diamine (1.19 mmol), 0.278 g DIPEA (2.15 mmol) in 14 mL butan-1-ol gave after 48h a crude product which was purified by column chromatography (silica, CH_2_Cl_2_ + MeOH = 19 + 1→9 + 1) yielding 0.100 g **10** (22%, 0.22 mmol) as amorphous solid. IR = 3405, 2936, 1577, 1424, 1237, 1123, 1032, 792, 699; ^1^H NMR (CDCl_3_) δ = 1.79 (br, s, 4H, (CH_2_)_2_), 1.90–1.95 (m, 2H, 4-H), 2.05–2.17 (m, 2H, 6-H, 9-H), 2.29–2.37 (m, 1H, 6-H), 2.49 (br, t, *J* = 12.3 Hz, 1H, 9-H), 2.70–2.79 (m, 4H, N(CH_2_)_2_), 3.00–3.08 (m, 1H, 3-H), 3.29 (t, *J* = 9.5 Hz, 1H, 7-H), 3.47–3.55 (m, 1H, 8-H), 4.25 (s, 2H, NH_2_), 4.47 (s, 2H, NH_2_), 5.01 (s, 1H, 5′-H), 7.12-7.36 (m, 8H, aromatic H), 7.48 (d, *J* = 7.6 Hz, 2H, aromatic H); ^13^C NMR (CDCl_3_) δ = 23.60 ((CH_2_)_2_), 31.73 (C-4), 36.67 (C-8), 37.26 (C-9), 37.68 (C-6), 40.53 (C-3), 45.27 (N(CH_2_)_2_), 45.79 (C-7), 56.60 (C-1), 57.07 (C-5), 75.36 (C-5′), 126.17, 126.56, 126.82, 127.59, 128.43, 128.65 (aromatic C), 143.64, 144.65 (aromatic C_q_), 162.48 (C-6′), 163.22 (C-4′), 164.52 (C-2′); HRMS (EI+) calcd for C_28_H_34_N_6_: 454.2845; found: 454.2846.

*rac*-6-[(7*R*,8*R*)-7,8-diphenyl-1-(piperidin-1-yl)-3-azabicyclo[3.2.2]nonan-3-yl]pyrimidine-2,4-diamine **11**.

The reaction of 0.293 g 1-(piperidin-1-yl)-3-azabicyclononane **3** (0.81 mmol), 0.143 g 6-chloropyrimidine-2,4-diamine (0.99 mmol) and 0.211 g DIPEA (1.69 mmol) in 12 mL butan-1-ol gave after 48 h a crude product which was purified by column chromatography (1. alox neutral, CH + EtOAc + MeOH = 18 + 1 + 1; 2. silica, CH_2_Cl_2_ + MeOH = 9 + 1) yielding 0.025 g **11** (6%, 0.05 mmol) as amorphous solid. IR = 3403, 2929, 1578, 1425, 1235, 1152, 1103, 1032, 793, 753, 699; ^1^H NMR (CDCl_3_) δ = 1.44–1.52 (m, 2H, CH_2_), 1.57-1.66 (m, 4H, 2CH_2_), 1.90 (dd, *J* = 12.7, 9.9 Hz, 1H, 8-H), 1.98 (t, *J* = 11.9 Hz, 1H, 7-H), 2.08–2.22 (m, 2H, 5-H, 8-H), 2.24–2.31 (m, 1H, 7-H), 2.47–2.55 (m, 2H, 2NCH), 2.82–2.90 (m, 2H, 2NCH), 3.03 (d, *J* = 12.5 Hz, 1H, 4-H), 3.13 (d, *J* = 14.5 Hz, 1H, 2-H), 3.34–3.42 (m, 2H, 6-H, 9-H), 3.67–3.75 (m, 1H, 4-H), 4.35 (s, 2H, NH_2_), 4.56 (s, 2H, NH_2_), 5.04 (s, 1H, 5′-H), 5.03–5.07 (m, 1H, 2-H), 7.11 (d, *J* = 7.0 Hz, 2H, aromatic H), 7.17 (t, *J* = 7.2 Hz, 1H, aromatic H), 7.22–7.42 (m, 7H, aromatic H); ^13^C NMR (CDCl_3_) δ = 25.07 (CH_2_), 26.79 (2CH_2_), 30.91 (C-8), 38.04 (C-7), 39.26 (C-9), 43.77 (C-6), 44.86 (C-5), 46.90 (N(CH_2_)_2_), 49.44 (C-2), 50.51 (C-4), 60.09 (C-1), 75.38 (C-5′), 126.28, 126.40, 126.69, 128.30, 128.37, 128.75 (aromatic C), 143.69, 146.27 (aromatic C_q_), 162.18 (C-6′), 164.56 (C-2′), 164.62 (C-4′); HRMS (EI+) calcd for C_29_H_36_N_6_: 468.3001; found: 468.2999.

*rac*-6-[(7*R*,8R)-7,8-diphenyl-1-(pyrrolidin-1-yl)-3-azabicyclo[3.2.2]nonan-3-yl]pyrimidine-2,4-diamine **12**.

The reaction of 0.424 g 1-(pyrrolidin-1-yl)-3-azabicyclononane **4** (1.22 mmol), 0.354 g 6-chloropyrimidine-2,4-diamine (2.45 mmol) and 0.403 g DIPEA (3.12 mmol) in 10 mL butan-1-ol gave after 68 h a crude product which was purified by column chromatography (alox basic, CH_2_Cl_2_ + MeOH = 29 + 1→19 + 1) yielding 0.147 g **12** (26%, 0.323 mmol) as amorphous solid. IR = 3398, 2933, 1579, 1494, 1428, 1237, 793, 753, 699; ^1^H NMR (CDCl_3_) δ = 1.78–1.83 (m, 4H, (CH_2_)_2_), 1.96 (dd, *J* = 13.0, 9.9 Hz, 1H, 8-H), 2.06-2.12 (m, 2H, 5-H, 7-H), 2.27 (ddd, *J* = 12.3, 10.1, 1.8 Hz, 1H, 8-H), 2.34 (ddd, *J* = 12.9, 8.6, 1.8 Hz, 1H, 7-H), 2.75–2.81 (m, 2H, NCH_2_), 2.92–2.97 (m, 2H, NCH_2_), 3.08 (br d, *J* = 12.7 Hz, 1H, 4-H), 3.23 (d, *J* = 14.1 Hz, 1H, 2-H), 3.38-3.45 (m, 2H, 6-H, 9-H), 3.72 (dd, *J* = 12.8, 4.3 Hz, 1H, 4-H), 4.35 (s, 2H, NH_2_), 4.56 (s, 2H, NH_2_), 5.01-5.08 (m, 1H, 2-H), 5.04 (s, 1H, 5′H), 710–7.43 (m, 10H, aromatic H); ^13^C NMR (CDCl_3_) δ = 23.63 ((CH_2_)_2_), 32.00 (C-8), 38.76 (C-7), 38.92 (C-9), 43.75 (C-6), 45.34 (C-5), 45.71 (N(CH_2_)_2_), 49.36 (C-2), 50.49 (C-4), 58.34 (C-1), 75.41 (C-5′), 126.26, 126.40, 126.77, 128.28, 128.36, 128.74 (aromatic C), 143.62, 146.20 (aromatic C_q_), 162.24 (C-6′), 164.59 (C-2′, C-4′); HRMS (EI+) calcd for C_28_H_34_N_6_: 454.2845; found: 454.2852.

#### 4.2.2. General Procedure for the Synthesis of **13**–**16**

The respective bicyclononane **1**,**2**,**3**,**4**, 2,4-dichloro-6-methylpyrimidine and DIPEA were suspended in ethanol cooling with an ice bath for 2 h. Afterwards the reaction batch was warmed up to 20 °C and stirred until the reaction was completed. The reaction progress was monitored by thin-layer chromatography (silica, CH_2_Cl_2_ + MeOH = 19 + 1). When the reaction was completed, the mixture was diluted with CH_2_Cl_2_ and alkalized with 2*N* NaOH. The organic phase was washed with water, dried over anhydrous sodium sulfate, filtered and finally the solvent was removed in vacuo giving crude products which were purified by column chromatography.

*rac*-(7*R*,8*R*)-2-(2-chloro-6-methylpyrimidin-4-yl)-7,8-diphenyl-5-(piperidin-1-yl)-2-azabicyclo[3.2.2]nonane **13**.

The reaction of 1.07 g 5-(piperidin-1-yl)-2-azabicyclononane **1** (2.97 mmol), 0.532 g 2,4-dichloro-6-methylpyrimidine (3.27 mmol) and 0.768 g DIPEA (5.94 mmol) in 17 mL ethanol gave after 188 h a crude product which was purified by column chromatography (silica, CH_2_Cl_2_ + MeOH = 29 + 1) yielding 0.920 g **13** (64%, 1.89 mmol) as a pale yellow oil. IR = 2931, 1587, 1536, 1495, 1289, 1165, 1089, 757, 699; ^1^H NMR (CDCl_3_) δ = 1.43–1.51 (m, 2H, CH_2_), 1.56–1.68 (m, 4H, 2CH_2_), 1.88 (td, *J* = 13.8, 4.7 Hz, 1H, 4-H), 1.98–2.09 (m, 2H, 4-H, 6-H), 2.13–2.31 (m, 4H, 9-H, CH_3_), 2.33–2.43 (m, 2H, 6-H, 9-H), 2.52–2.70 (m, 4H, N(CH_2_)_2_), 3.17–3.28 (m, 2H, 3-H, 7-H), 3.38–3.57 (m, 1H, 8-H), 5.97 (br, 1H, 5′-H), 7.08–7.40 (m, 10H, aromatic H); ^13^C NMR (CDCl_3_) δ = 23.96 (CH_3_), 24.90 (CH_2_), 26.64 (2CH_2_), 31.41 (C-4), 35.99 (C-6, C-9), 37.39 (C-8), 41.24 (C-3), 46.05 (C-7), 46.36 (N(CH_2_)_2_), 58.29 (C-5), 99.98 (C-5′), 126.54, 126.79, 126.95, 127.42, 128.56, 128.82 (aromatic C), 142.65, 143.58 (aromatic C_q_), 160.10 (C-2′), 162.65 (C-4′), 167.48 (C-6′); HRMS (EI+) calcd for C_30_H_35_ClN_4_: 486.2550; found: 486.2554.

*rac*-(7*R*,8*R*)-2-(2-chloro-6-methylpyrimidin-4-yl)-7,8-diphenyl-5-(pyrrolidin-1-yl)-2-azabicyclo[3.2.2]nonane **14**.

The reaction of 0.949 g 5-(pyrrolidin-1-yl)-2-azabicyclononane **2** (2.74 mmol), 0.490 g 2,4-dichloro-6-methylpyrimidine (3.01 mmol) and 0.701 g DIPEA (5.42mmol) in 15 mL ethanol gave after 44 h a crude product which was purified by column chromatography (silica, CH_2_Cl_2_ + MeOH = 29 + 1) yielding 0.610 g **14** (47%, 1.29 mmol) as a pale yellow oil. IR = 2955, 1588, 1485, 1358, 1290, 1208, 1165, 1049, 757, 700; ^1^H NMR (CDCl_3_) δ = 1.76–1.85 (m, 4H, (CH_2_)_2_), 1.91–2.07 (m, 2H, 4-H), 2.13–2.28 (m, 5H, 6-H, 9-H, CH_3_), 2.29–2.39 (m, 1H, 6-H), 2.49 (t, *J* = 12.3 Hz, 1H, 9-H), 2.70–2.81 (m, 4H, N(CH_2_)_2_), 3.23–3.34 (m, 2H, 3-H, 7-H), 3.47–3.59 (m, 1H, 8-H), 6.01 (br, 1H, 5′-H), 7.08–7.65 (m, 10H, aromatic H); ^13^C NMR (CDCl_3_) δ = 23.58 ((CH_2_)_2_), 23.99 (CH_3_), 31.73 (C-4), 35.04 (C-8), 36.61 (C-6, C-9), 40.82 (C-3), 45.08 (C-7), 45.28 (N(CH_2_)_2_), 56.82 (C-1), 59.08 (C-5), 100.02 (C-5′), 126.53, 126.78, 126.98, 127.61, 128.58, 128.88 (aromatic C), 142.65, 143.36 (aromatic C_q_), 160.15 (C-2′), 162.66 (C-4′), 167.51 (C-6′); HRMS (EI+) calcd for C_29_H_33_ClN_4_: 472.2394; found: 472.2390.

rac-(7*R*,8*R*)-3-(2-chloro-6-methylpyrimidin-4-yl)-6,9-diphenyl-1-(piperidin-1-yl)-3-azabicyclo[3.2.2]nonane **15**.

The reaction of 1.05 g 1-(piperidin-1-yl)-3-azabicyclononane **3** (2.91 mmol), 0.522 g 2,4-dichloro-6-methylpyrimidine (3.20 mmol) and 0.764 g DIPEA (5.91 mmol) in 17 mL ethanol gave after 140h a crude product which was purified by column chromatography (silica, CH_2_Cl_2_ + MeOH = 29 + 1) yielding 0.400 g **15** (28%, 0.82 mmol) as a pale yellow oil. IR = 2929, 1589, 1495, 1308, 1247, 1229, 1174, 1061, 753, 699; ^1^H NMR (CDCl_3_) δ = 1.46–1.54 (m, 2H, CH_2_), 1.61–1,69 (m, 4H, 2CH_2_), 1.94 (br, t, *J* = 11.2 Hz, 1H, 8-H), 2.00–2.12 (m, 2H, 7-H, 8-H), 2.22 (br, s, 1H, 5-H), 2.32 (s, 3H, CH_3_), 2.28–2.37 (m, 1H, 7-H), 2.58–2.68 (m, 2H, 2NCH), 2.84–2.95 (m, 2H, 2NCH), 3.17 (br, d, *J* = 12.2. Hz, 1H, 4-H), 3.27 (br, d, *J* = 14.5 Hz, 1H, 2-H), 3.37–3.46 (m, 2H, 6-H, 9-H), 3.75 (br, 1H, 4-H), 5.13 (br, 1H, 2-H), 6.10 (br, s, 1H, 5′-H), 7.02 (d, *J* = 7.2 Hz, 2H, aromatic H), 7.16–7.43 (m, 8H, aromatic H); ^13^C NMR (CDCl_3_) δ = 24.02 (CH_3_), 24.96 (CH_2_), 26.74 (2CH_2_), 30.20 (C-8), 38.16 (C-7), 38.57 (C-9), 43.70 (C-6), 44.92 (C-5), 46.94 (N(CH_2_)_2_), 49.81 (C-2), 50.68 (C-4), 60.20 (C-1), 99.85 (C-5′), 126.58, 126.65, 126.66, 127.87, 128.50, 128.88 (aromatic C), 142.86, 145.71 (aromatic C_q_), 159.94 (C-2′), 163.69 (C-4′), 167.71 (C-6′); HRMS (EI+) calcd for C_30_H_35_ClN_4_: 486.2550; found: 486.2571.

*rac*-(7*R*,8*R*)-3-(2-chloro-6-methylpyrimidin-4-yl)-6,9-diphenyl-1-(pyrrolidin-1-yl)-3-azabicyclo[3.2.2]nonane **16**.

The reaction 0.385 g of 1-(pyrrolidin-1-yl)-3-azabicyclononane **4** (1.11 mmol), 0.199 g 2,4-dichloro-6-methylpyrimidine (1.22 mmol) and 0.291 g DIPEA (2.25 mmol) in 6 mL ethanol gave after 44 h a crude product which was purified by column chromatography (silica, CH_2_Cl_2_ + MeOH = 19 + 1) yielding 0.225 g **16** (45%, 0.50 mmol) as a pale yellow oil. IR = 2926, 1589, 1494, 1173, 1056, 753, 700; ^1^H NMR (CDCl_3_) δ = 1.80–1.87 (m, 4H, (CH_2_)_2_), 2.00 (br, t, *J* = 11.5 Hz, 1H, 8-H), 2.09–2.23 (m, 3H, 5-H, 7-H, 8-H), 2.32 (s, 3H, CH_3_), 2.35–2.42 (m, 1H, 7-H), 2.74–2.82 (m, 2H, 2NCH), 2.93–3.00 (m, 2H, 2NCH), 3.18–3.51 (m, 4H, 2-H, 4-H, 6-H, 9-H), 3.77 (br, 1H, 4-H), 5.08 (br, 1H, 2-H), 6.10 (br, s, 1H, 5′-H), 7.02 (d, *J* = 7.1 Hz, 2H, aromatic H), 7.16–7.43 (m, 8-H, aromatic H); ^13^C NMR (CDCl_3_) δ = 23.67 ((CH_2_)_2_), 24.00 (CH_3_), 31.55 (C-8), 38.20 (C-9), 38.90 (C-7), 43.64 (C-6), 45.26 (C-5), 45.74 (N(CH_2_)_2_), 49.58 (C-2), 50.69 (C-4), 58.35 (C-1), 99.90 (C-5′), 126.56, 126.66, 126.75, 127.85, 128.49, 128.88 (aromatic C), 142.84, 145.68 (aromatic C_q_), 159.94 (C-2′), 163.65 (C-4′), 167.65 (C-6′); HRMS (EI+) calcd for C_29_H_33_ClN_4_: 472.2394; found: 472.2397.

#### 4.2.3. General Procedure for the Synthesis of **17**–**20**

The respective bicyclononane derivate **19**–**22** was mixed with excess pyrrolidine and acetonitrile and was refluxed at 105 °C for 20 h. The reaction progress was monitored by thin-layer chromatography (silica, CH_2_Cl_2_ + MeOH = 19 + 1 and 9 + 1). When the reaction was completed, the mixture was diluted with CH_2_Cl_2_ and alkalized with 2*N* NaOH. The organic phase was washed with water, dried over anhydrous sodium sulfate, filtered and finally the solvent was removed in vacuo giving crude products which were purified by column chromatography.

*rac*-(7*R*,8*R*)-2-[6-methyl-2-(pyrrolidin-1-yl)pyrimidin-4-yl]-7,8-diphenyl-5-(piperidin-1-yl)-2-azabicyclo[3.2.2]nonane **17**.

The reaction of 0.150 g **13** (0.31 mmol) and 0.291 g pyrrolidine (3.10 mmol) in 10 mL acetonitrile gave a crude product which was purified by column chromatography (silica, CH_2_Cl_2_ + MeOH = 29 + 1) yielding 0.110 g **17** (68%, 0.21 mmol) as amorphous solid. IR = 2930, 2854, 1571, 1449, 1414, 1342, 1251, 1220, 1174,1104, 1032, 787, 698; ^1^H NMR (CDCl_3_) δ = 1.42–1.50 (m, 2H, CH_2_), 1.56–1.66 (m, 4H, 2CH_2_), 1.79–1.87 (m, 4H, (CH_2_)_2_), 1.85–2.05 (m, 3H, 4-H, 6-H), 2.12 (br, t, *J* = 10.8 Hz, 1H, 9-H), 2.20 (s, 3H, CH_3_), 2.31–2.44 (m, 2H, 6-H, 9-H), 2.51–2.70 (m, 4H, N(CH_2_)_2_), 3.07 (br, t, *J* = 12.3 Hz, 1H, 3-H), 3.22 (t, *J* = 9.5 Hz, 1H, 7-H), 3.34–3.50 (m, 5H, 8-H, N(CH_2_)_2_), 5.63 (s, 1H, 5′-H), 7.09–7.25 (m, 6H, aromatic H), 7.31 (t, *J* = 7.4 Hz, 2H, aromatic H), 7.45 (d, *J* = 7.5 Hz, 2H, aromatic H); ^13^C NMR (CDCl_3_) δ = 24.61 (CH_3_), 25.02 (CH_2_), 25.46 ((CH_2_)_2_), 26.73 (2CH_2_), 31.55 (C-4), 36.57 (C-6, C-9), 37.41 (C-8), 41.00 (C-3), 46.21 (C-7), 46.36 (N(CH_2_)_2_), 46.40 (N(CH_2_)_2_), 55.84 (C-1), 58.48 (C-5), 90.98 (C-5′), 126.08, 126.40, 126.88, 127.52, 128.37, 128.43 (aromatic C), 143.83, 144.78 (aromatic C_q_), 160.43 (C-2′), 162.00 (C-4′), 165.80 (C-6′); HRMS (EI+) calcd for C_34_H_43_N_5_: 521.3519; found: 521.3530.

*rac*-(7*R*,8*R*)-2-[6-methyl-2-(pyrrolidin-1-yl)pyrimidin-4-yl]-7,8-diphenyl-5-(pyrrolidin-1-yl)-2-azabicyclo[3.2.2]nonane **18**.

The reaction of 0.120 g **14** (0.25 mmol) and 0.178 g pyrrolidine (2.50 mmol) in 8mL acetonitrile gave a crude product which was purified by column chromatography (silica, CH_2_Cl_2_ + MeOH = 29 + 1) yielding 0.072 g **18** (56%, 0.14 mmol) as amorphous solid. IR = 2946, 2869, 1571, 1447, 1413, 1341, 1251, 1173, 787, 698; ^1^H NMR (CDCl_3_) δ = 1.79–1.90 (m, 8H, 2(CH_2_)_2_), 1.96–2.02 (m, 2H, 4-H), 2.13–2.22 (m, 2H, 6-H, 9-H), 2.21 (s, 3H, CH_3_), 2.30–2.38 (m, 1H, 6-H), 2.52–2.61 (m, 1H, 9-H), 2.77–2.88 (m, 4H, N(CH_2_)_2_), 3.05–3.15 (m, 1H, 3-H), 3.29 (t, *J* = 9.5 Hz, 1H, 7-H), 3.40–3.57 (m, 5H, 8-H, N(CH_2_)_2_), 5.64 (s, 1H, 5′-H), 7.10–7.25 (m, 6H, aromatic H), 7.34 (t, *J* = 7.5 Hz, 2H, aromatic H), 7.47 (d, *J* = 7.5 Hz, 2H, aromatic H); ^13^C NMR (CDCl_3_) δ = 23.67 ((CH_2_)_2_), 24.58 (CH_3_), 25.47 ((CH_2_)_2_), 31.94 (C-4), 36.60 (C-8), 36.80 (C-9), 37.31 (C-6), 40.18 (C-3), 45.53 (N(CH_2_)_2_), 45.81 (C-7), 46.45 (N(CH_2_)_2_), 55.77 (C-1), 57.86 (C-5), 91.01 (C-5′), 126.15, 126.54, 126.82, 127.61, 128.42, 128.52 (aromatic C), 143.52, 144.45 (aromatic C_q_), 160.38 (C-2′), 161.96 (C-4′), 165.85 (C-6′); HRMS (EI+) calcd for C_33_H_41_N_5_: 507.3362; found: 507.3366.

*rac*-(7*R*,8*R*)-3-[6-methyl-2-(pyrrolidin-1-yl)pyrimidin-4-yl]-6,9-diphenyl-1-(piperidin-1-yl)-3-azabicyclo[3.2.2]nonane **19**.

The reaction of 0.139 g **15** (0.29 mmol), 0.244 g pyrrolidine (3.38 mmol) and 0.088 g DIPEA (0.68 mmol) in 8mL acetonitrile gave a crude product which was purified by column chromatography (silica, CH_2_Cl_2_ + MeOH = 19 + 1) yielding 0.130 g **19** (86%, 0.25 mmol) as amorphous solid. IR = 2928, 1572, 1453, 1419, 1306, 1247, 1218, 1179, 787, 753, 699; ^1^H NMR (CDCl_3_) δ = 1.44–1.52 (m, 2H, CH_2_), 1.58–1.68 (m, 4H, 2CH_2_), 1.84–1.99 (m, 5H, 8-H, (CH_2_)_2_), 2.00–2.16 (m, 3H, 5-H, 7-H, 8-H), 2.18–2.23 (m, 1H, 7-H), 2.24 (s, 3H, CH_3_), 2.54–2.64 (m, 2H, 2NCH), 2.91–3.01 (m, 2H, 2NCH), 3.05 (d, *J* = 12.8 Hz, 1H, 4-H), 3.17 (br, d, *J* = 14.6 Hz, 1H, 2-H), 3.38 (br, t, *J* = 9.3 Hz, 1H, 9-H), 3.45 (t, *J* = 9.4 Hz, 1H, 6-H), 3.50–3.67 (m, 4H, N(CH_2_)_2_), 3.80 (br, 1H, 4-H), 5.39 (br, 1H, 2-H), 5.62 (s, 1H, 5′-H), 7.09 (d, *J* = 7.2 Hz, 2H, aromatic H), 7.15–7.44 (m, 8H, aromatic H); ^13^C NMR (CDCl_3_) δ = 24.61 (CH_3_), 25.08 (CH_2_), 25.60 ((CH_2_)_2_), 26.64 (2CH_2_), 29.53 (C-8), 38.92 (C-7), 39.43 (C-9), 44.02 (C-6), 45.03 (C-5), 46.55 (N(CH_2_)_2_), 47.14 (N(CH_2_)_2_), 49.32 (C-2), 50.31 (C-4), 60.48 (C-1), 90.85 (C-5′), 126.38, 126.44, 126.69, 128.36, 128.39, 128.79 (aromatic C), 143.62, 146.25 (aromatic C_q_), 160.27 (C-2′), 163.07 (C-4′), 166.06 (C-6′); HRMS (EI+) calcd for C_34_H_43_N_5_: 521.3519; found: 521.3555.

*rac*-(7*R*,8*R*)-3-[6-methyl-2-(pyrrolidin-1-yl)pyrimidin-4-yl]-6,9-diphenyl-1-(pyrrolidin-1-yl)-3-azabicyclo[3.2.2]nonane **20**.

The reaction of 0.080 g **16** (0.17 mmol), 0.051 g pyrrolidine (0.72 mmol) and 0.071 g DIPEA (0.55 mmol) in 3mL acetonitrile gave a crude product which was purified by column chromatography (silica, CH_2_Cl_2_ + MeOH = 19 + 1) yielding 0.029 g **20** (33%, 0.06 mmol) as amorphous solid. IR = 2961, 1572, 1453, 1419, 1177, 753, 699; ^1^H NMR (CDCl_3_) δ = 1.80–1.87 (m, 4H, (CH_2_)_2_), 1.91–2.00 (m, 5H, 8-H, N(CH_2_)_2_), 2.10 (br, s, 1H, 5-H), 2.12–2.21 (m, 1H, 7-H), 2.24 (s, 3H, CH_3_), 2.27 (br, t, *J* = 12.0 Hz, 1H, 8-H), 2.34–2.42 (m, 1H, 7-H), 2.80–2.88 (m, 2H, 2NCH), 3.01–3.09 (m, 2H, 2NCH), 3.10 (d, *J* = 13.2 Hz, 1H, 4-H), 3.25 (d, *J* = 14.4 Hz, 1H, 2-H), 3.39–3.48 (m, 2H, 6-H, 9-H), 3.52–3.64 (m, 4H, N(CH_2_)_2_), 3.84 (br, 1H, 4-H), 5.34 (br, 1H, 2-H), 5.64 (s, 1H, 5′-H), 7.08 (d, *J* = 7.3 Hz, 2H, aromatic H), 7.15–7.44 (m, 8H, aromatic H); ^13^C NMR (CDCl_3_) δ = 23.99 ((CH_2_)_2_), 24.63 (CH_3_), 25.60 ((CH_2_)_2_), 32.07 (C-8), 38.97 (C-9), 39.11 (C-7), 43.98 (C-6), 45.59 (C-5), 46.00 (N(CH_2_)_2_), 46.51 (N(CH_2_)_2_), 48.24 (C-2), 50.43 (C-4), 59.07 (C-1), 90.94 (C-5′), 126.36, 126.47, 126.80, 128.32, 128.38, 128.79 (aromatic C), 143.51, 146.02 (aromatic C_q_), 160.30 (C-2′), 163.11 (C-4′), 166.16 (C-6′); HRMS (EI+) calcd for C_33_H_41_N_5_: 507.3362; found: 507.3346.

#### 4.2.4. General Procedure for the Synthesis of **21**–**24**

The respective bicyclononane derivate **13**–**16** was mixed with excess piperidine and acetonitrile and was refluxed at 105 °C for 48 h. The reaction progress was monitored by thin-layer chromatography (silica, CH_2_Cl_2_ + MeOH = 9+1). When the reaction was completed, the mixture was diluted with CH_2_Cl_2_ and alkalized with 2*N* NaOH. The organic phase was washed with water, dried over anhydrous sodium sulfate, filtered and finally the solvent was removed in vacuo giving crude products which were purified by column chromatography.

*rac*-(7*R*,8*R*)-2-[6-methyl-2-(piperidin-1-yl)pyrimidin-4-yl]-7,8-diphenyl-5-(piperidin-1-yl)-2-azabicyclo[3.2.2]nonane **21**.

The reaction of 0.125 g **13** (0.26 mmol) and 0.219 g piperidine (2.57 mmol) in 7 mL acetonitrile gave a crude product which was purified by column chromatography (silica, CH_2_Cl_2_ + MeOH = 49 + 1) yielding 0.095 g **21** (69%, 0.18 mmol) as amorphous solid. IR = 2930, 2850, 1572, 1445, 1414, 1245, 1031, 787, 698; ^1^H NMR (CDCl_3_) δ = 1.41–1.72 (m, 12H, 6CH_2_), 1.82–2.03 (m, 3H, 4-H, 6-H), 2.07–2.17 (m, 1H, 9-H), 2.18 (s, 3H, CH_3_), 2.33–2.44 (m, 2H, 6-H, 9-H), 2.52–2.71 (m, 4H, N(CH_2_)_2_), 3.08 (br, t, *J* = 12.4 Hz, 1H, 3-H), 3.23 (t, *J* = 9.5 Hz, 1H, 7-H), 3.44–3.52 (m, 1H, 8-H), 3.60 (br, s, 4H, N(CH_2_)_2_), 5.61 (s, 1H, 5′-H), 7.10–7.34 (m, 8H, aromatic H), 7.43 (d, *J* = 7.5 Hz, 2H, aromatic H); ^13^C NMR (CDCl_3_) δ = 24.65 (CH_3_), 25.03, 25.90, 26.71 (6CH_2_), 31.65 (C-4), 36.39 (C-9), 36.57 (C-6), 37.37 (C-8), 40.95 (C-3), 44.76 (N(CH_2_)_2_), 46.20 (C-7), 46.41 (N(CH_2_)_2_), 55.73 (C-1), 58.44 (C-5), 91.15 (C-5′), 126.12, 126.42, 126.90, 127.51, 128.40, 128.51 (aromatic C), 143.76, 144.77 (aromatic C_q_), 161.71 (C-2′), 162.15 (C-4′), 165.94 (C-6′); HRMS (EI+) calcd for C_35_H_45_N_5_: 535.3675; found: 535.3716.

*rac*-(7*R*,8*R*)-2-[6-methyl-2-(piperidin-1-yl)pyrimidin-4-yl]-7,8-diphenyl-5-(pyrrolidin-1-yl)-2-azabicyclo[3.2.2]nonane **22**.

The reaction of 0.100 g **14** (0.21 mmol) and 0.186 g piperidine (2.18 mmol) in 7 mL acetonitrile gave a crude product which was purified by column chromatography (silica, CH_2_Cl_2_ + MeOH = 29 + 1) yielding 0.067 g **22** (62%, 0.13 mmol) as amorphous solid. IR = 2929, 2850, 1572, 1496, 1445, 1414, 1355, 1244, 1029, 787, 699; ^1^H NMR (CDCl_3_) δ = 1.45–1.54 (m, 4H, (CH_2_)_2_), 1.55–1.62 (m, 2H, CH_2_), 1,76–1.84 (m, 4H, 2CH_2_), 1.94–2.01 (m, 2H, 4-H), 2.06–2.17 (m, 2H, 6-H, 9-H), 2.18 (s, 3H, CH_3_), 2.28–2.38 (m, 1H, 6-H), 2.51 (t, *J* = 12.3 Hz, 1H, 9-H), 2.72–2.84 (m, 4H, N(CH_2_)_2_), 3.04–3.15 (m, 1H, 3-H), 3.29 (t, *J* = 9.6 Hz, 1H, 7-H), 3.49–3.57 (m, 1H, 8-H), 3.58–3.66 (m, 4H, N(CH_2_)_2_), 5.62 (s, 1H, 5′-H), 7.10–7.25 (m, 6H, aromatic H), 7.32 (t, *J* = 7.5 Hz, 2H, aromatic H), 7.45 (d, *J* = 7.5 Hz, 2H, aromatic H); ^13^C NMR (CDCl_3_) δ = 23.64 (2CH_2_), 24.66 (CH_3_), 25.03 (CH_2_), 25.90 ((CH_2_)_2_), 31.92 (C-4), 36.70 (C-8), 36.92 (C-9), 37.58 (C-6), 40.81 (C-3), 44.76 (N(CH_2_)_2_), 45.36 (N(CH_2_)_2_), 45.91 (C-7), 55.74 (C-1), 57.19 (C-5), 91.15 (C-5′), 126.11, 126.46, 126.83, 127.59, 128.39, 128.53 (aromatic C), 143.67, 144.71 (aromatic C_q_), 161.74 (C-2′), 162.14 (C-4′), 165.97 (C-6′); HRMS (EI+) calcd for C_34_H_43_N_5_: 521.3519; found: 521.3533.

*rac*-(7*R*,8*R*)-3-[6-methyl-2-(piperidin-1-yl)pyrimidin-4-yl]-6,9-diphenyl-1-(piperidin-1-yl)-3-azabicyclo[3.2.2]nonane **23**.

The reaction of 0.126 g **15** (0.26 mmol) and 0.218 g piperidine (2.56 mmol) in 8mL acetonitrile gave a crude product which was purified by column chromatography (silica, CH_2_Cl_2_ + MeOH = 29 + 1) yielding 0.094 g **23** (69%, 0.18 mmol) as amorphous solid. IR = 2929, 2850, 1573, 1444, 1418, 1306, 1244, 1212, 1099, 1025, 786, 753, 699; ^1^H NMR (CDCl_3_) δ = 1.42–1.51 (m, 2H, CH_2_), 1.54–1.70 (m, 10H, 5CH_2_), 1.83–1.92 (m, 1H, 8-H), 1.98–2.16 (m, 3H, 5-H, 7-H, 8-H), 2.17–2.25 (m, 1H, 7-H), 2.22 (s, 3H, CH_3_), 2.54–2.64 (m, 2H, 2NCH), 2.83–2.94 (m, 2H, 2NCH), 3.03 (d, *J* = 12.8 Hz, 1H, 4-H), 3.17 (br, d, *J* = 14.4 Hz, 1H, 2-H), 3.37 (br, t, *J* = 9.3 Hz, 1H, 9-H), 3.45 (t, *J* = 9.3 Hz, 1H, 6-H), 3.74–3.87 (m, 5H, 4-H, N(CH_2_)_2_), 5.33 (br, 1H, 2-H), 5.61 (s, 1H, 5′-H), 7.10 (d, *J* = 7.3 Hz, 2H, aromatic H), 7.15–7.43 (m, 8H, aromatic H); ^13^C NMR (CDCl_3_) δ = 24.66 (CH_3_), 25.05 (2CH_2_), 25.92 (2CH_2_), 26.71 (2CH_2_), 29.57 (C-8), 38.85 (C-7), 39.41 (C-9), 43.99 (C-6), 44.83 (N(CH_2_)_2_), 44.98 (C-5), 47.17 (N(CH_2_)_2_), 49.38 (C-2), 50.21 (C-4), 60.42 (C-1), 90.94 (C-5′), 126.39, 126.45, 126.70, 128.36, 128.40, 128.78 (aromatic C), 143.68, 146.22 (aromatic C_q_), 161.50 (C-2′), 163.04 (C-4′), 166.29 (C-6′); HRMS (EI+) calcd for C_35_H_45_N_5_: 535.3675; found: 535.3695.

*rac*-(7*R*,8*R*)-3-[6-methyl-2-(piperidin-1-yl)pyrimidin-4-yl]-6,9-diphenyl-1-(pyrrolidin-1-yl)-3-azabicyclo[3.2.2]nonane **24**.

The reaction of 0.090 g **16** (0.19 mmol) and 0.173 g piperidine (2.03 mmol) in 5mL acetonitrile gave a crude product which was purified by column chromatography (silica, CH_2_Cl_2_ + MeOH = 19 + 1) yielding 0.054 g **24** (53%, 0.10 mmol) as amorphous solid. IR = 2926, 2850, 1551, 1442, 1416, 1288, 1243, 1180, 1024, 786, 751, 698; ^1^H NMR (CDCl_3_) δ = 1.56–1.69 (m, 6H, (CH_2_)_3_), 1.78–1.85 (m, 4H, (CH_2_)_2_), 1.95 (br, t, *J* = 11.3 Hz, 1H, 8-H), 2.11 (br, s, 1H, 5-H), 2.14 (br, t, *J* = 11.6 Hz, 1H, 7-H), 2.22 (s, 3H, CH_3_), 2.24 (br, t, *J* = 12.1 Hz, 1H, 8-H), 2.34–2.40 (m, 1H, 7-H), 2.77–2.86 (m, 2H, 2NCH), 2.94–3.03 (m, 2H, 2NCH), 3.10 (d, *J* = 12.9 Hz, 1H, 4-H), 3.25 (d, *J* = 14.3 Hz, 1H, 2-H), 3.38–3.47 (m, 2H, 6-H 9-H), 3.74–3.81 (m, 4H, N(CH_2_)_2_), 3.86 (br, 1H, 4-H), 5.22 (br, 1H, 2-H), 5.64 (s, 1H, 5′-H), 7.09 (d, *J* = 7.4 Hz, 2H, aromatic H), 7.15–7.44 (m, 8H, aromatic H); ^13^C NMR (CDCl_3_) δ = 23.99 ((CH_2_)_2_), 24.67 (CH_3_), 25.04 (CH_2_), 25.87 (2CH_2_), 32.09 (C-8), 38.98 (C-9), 39.17 (C-7), 43.94 (C-6), 44.84 (N(CH_2_)_2_), 45.52 (C-5), 45.91 (N(CH_2_)_2_), 48.52 (C-2), 50.34 (C-4), 58.76 (C-1), 91.07 (C-5′), 126.34, 126.44, 126.79, 128.32, 128.37, 128.77 (aromatic C), 143.58, 146.09 (aromatic C_q_), 161.50 (C-2′), 163.14 (C-4′), 166.23 (C-6′); HRMS (EI+) calcd for C_34_H_43_N_5_: 521.3519; found: 521.3546.

### 4.3. Biological Tests

#### 4.3.1. In Vitro Microplate Assay against *P. falciparum*

In vitro activity against erythrocytic stages of *P. falciparum* was determined using a ^3^H-hypoxanthine incorporation assay [25,26] using the drug sensitive NF54 strain [27] or the chloroquine- and pyrimethamine-resistant K1 strain [28]. Compounds were dissolved in DMSO at 10 mg/mL and further diluted in medium before being added to parasite cultures incubated in RPMI 1640 medium without hypoxanthine, supplemented with HEPES (5.94 g/L), NaHCO_3_ (2.1 g/L), neomycin (100 U/mL), Albumax^®^ (5 g/L) and washed human red cells A+ at 2.5% haematocrit (0.3% parasitaemia). Serial drug dilutions of eleven 3-fold dilution steps covering a range from 100 to 0.002 μg/mL were prepared. The 96-well plates were incubated in a humidified atmosphere at 37 °C; 4% CO_2_, 3% O_2_, 93% N_2_. After 48 h, 0.05 mL of ^3^H-hypoxanthine (= 0.5 μCi) was added to each well of the plate. The plates were incubated for a further 24 h under the same conditions. The plates were then harvested with a Betaplate™ cell harvester (Wallac, Zurich, Switzerland), and the red blood cells transferred onto a glass fibre filter then washed with distilled water. The dried filters were inserted into a plastic foil with 10 mL of scintillation fluid, and counted in a Betaplate™ liquid scintillation counter (Wallac, Zurich, Switzerland). IC_50_ values were calculated from sigmoidal inhibition curves by linear regression [29] using Microsoft Excel. Chloroquine (Sigma C6628) was used as control.

#### 4.3.2. In Vitro Microplate Assay against *T. brucei rhodesiense*

Minimum essential medium (50 µL) supplemented with 25 mM HEPES, 1g/L additional glucose, 1% MEM non-essential amino acids (100×), 0.2 mM 2-mercaptoethanol, 1mM Na-pyruvate and 15% heat-inactivated horse serum was added to each well of a 96-well microtiter plate. Serial drug dilutions of eleven 3-fold dilution steps covering a range from 100 to 0.002 μg/mL were prepared. Then 4 × 10^3^ bloodstream forms of *T. b. rhodesiense* STIB 900 in 50 µL was added to each well and the plate incubated at 37 °C under a 5 % CO_2_ atmosphere for 70 h. Next, 10 µL resazurin solution (resazurin, 12.5 mg in 100 mL double-distilled water) was added to each well and incubation continued for a further 2–4 h [30]. Then the plates were read with a Spectramax Gemini XS microplate fluorometer (Molecular Devices Cooperation, Sunnyvale, CA, USA) using an excitation wave length of 536 nm and an emission wave length of 588 nm. Data were analyzed with the graphic programme Softmax Pro (Molecular Devices Cooperation, Sunnyvale, CA, USA), which calculated IC_50_ values by linear regression [29] and 4-parameter logistic regression from the sigmoidal dose inhibition curves. Melarsoprol (Arsobal Sanofi-Aventis, received from WHO) was used as control.

#### 4.3.3. In Vitro Cytotoxicity with L-6 Cells

Assays were performed in 96-well microtiter plates, each well containing 0.1 mL of RPMI 1640 medium supplemented with 1% L-glutamine (200 mM) and 10% fetal bovine serum, and 4000 L-6 cells (a primary cell line derived from rat skeletal myoblasts, ATCC CRL-1458™) [31,32]. Serial drug dilutions of eleven 3-fold dilution steps covering a range from 100 to 0.002 μg/mL were prepared. After 70 h of incubation the plates were inspected under an inverted microscope to assure growth of the controls and sterile conditions. Then, 0.01 mL resazurin solution (resazurin, 12.5 mg in 100 mL double-distilled water) was added to each well and the plates incubated for another 2 h. The plates were read with a Spectramax Gemini XS microplate fluorometer (Molecular Devices Cooperation, Sunnyvale, CA, USA) using an excitation wave length of 536 nm and an emission wave length of 588 nm. The IC_50_ values were calculated by linear regression [29] from the sigmoidal dose inhibition curves using SoftmaxPro software (Molecular Devices Cooperation, Sunnyvale, CA, USA). Podophyllotoxin (Sigma P4405) was used as control.

## 5. Conclusions

The synthesis and the evaluation of the structure–activity relationships of the antiprotozoal activities of azabicyclo-nonane pyrimidine hybrids were reported. A number of structure–activity relationships were revealed. A couple of 1-piperidino-3-azabicyclo-nonanes with a 2-aminopyrimidine moiety exhibited in vitro activity against *P. falciparum* NF54 in submicromolar concentration and high selectivity. A 2-azabicyclo-nonane with a pyrrolidino substituent on both the bicyclic ring system as well as on the pyrimidine ring even possessed improved activity against the multiresistant K1 strain of *P. falciparum*. Three 5-pyrrolidino-2-azabicyclo-nonanes have shown good trypanocidal activity and selectivity. Further modifications of the substitution pattern of the pyrimidine moiety will be investigated in a future project.

## Figures and Tables

**Table 1:** Activities of compounds **1–24** against *P. falciparum* NF54, *P. falciparum* K1, *T. brucei rhodesiense* (STIB900) and L-6 cells, expressed as IC_50_ (µM) ^a^.

Compounds	*P. falciparum* NF54 ^b^	S.I. = IC_50_ (Cyt.)/IC_50_ (*P.f.NF54*)	*P. falciparum K1* ^c^	S.I. = IC_50_ (Cyt.)/IC_50_ (*P.f.K1*)	*T. brucei rhodesiense STIB900*	S.I. = IC_50_ (Cyt.)/IC_50_ (*T.b.r*.)	Cytotoxicity L-6 Cells
**1**			0.64 ^d^	140.2 ^d^	6.57 ^d^	13.70 ^d^	89.74 ^d^
**2**			0.56 ^d^	215.0 ^d^	1.16 ^d^	103.0 ^d^	120.4 ^d^
**3**			0.75 ^d^	162.9 ^d^	2.02 ^d^	60.5 ^d^	122.3 ^d^
**4**	0.072 ^e^	1013	0.095 ^d^	764.8 ^d^	1.00 ^d^	72.90 ^d^	72.93 ^d^
**5**	0.212 ± 0.012	50.2	0.140 ± 0.033	75.95 ^d^	3.31 ± 0.235	3.22	10.65 ± 1.935
**6**	0.088 ± 0.057	142.5			0.161 ± 0	77.89	12.54 ± 3.307
**7**	0.024 ± 0.004	333.3	0.051 ± 0.024	155.6	0.573 ± 0.132	13.96	8.00 ± 1.892
**8**	0.062 ± 0.001	72.3			0.322 ± 0.151	13.91	4.48 ± 1.444
**9**	0.051 ± 0.007	190.8	0.094 ± 0.004	103.6	1.56 ± 0.643	6.24	9.73 ± 0.661
**10**	0.079 ± 0.001	142.5	0.216 ± 0.037	52.19	0.853 ± 0.016	13.20	11.26 ± 0.033
**11**	0.023 ± 0.001	188.3	0.087 ± 0.002	49.51	0.201 ± 0.039	21.54	4.33 ± 0.235
**12**	0.033 ± 0.016	337.3	0.172 ± 0.051	64.87	0.946 ± 0.374	11.76	11.13 ± 0.836
**13**	0.694 ± 0.018	4.47	0.567 ± 0.047	5.45	1.55 ± 0.047	2.00	3.10 ± 0.051
**14**	1.04 ± 0.302	9.25	0.366 ± 0.095	26.30	1.64 ± 0.022	5.86	9.62 ± 1.099
**15**	1.49 ± 0.784	18.23	2.075 ± 0.430	13.09	9.51 ± 0.914	2.86	27.16 ± 11.77
**16**	0.281 ± 0.006	44.63	0.292 ± 0.021	42.97	4.31 ± 0.021	2.91	12.54 ± 1.437
**17**	0.617 ± 0.008	37.65			0.575 ± 0.033	40.40	23.23 ± 9.928
**18**	0.136 ± 0.016	110.7	0.059 ± 0.002	254.5	0.095 ± 0.032	158.4	15.05 ± 0.483
**19**	5.29 ± 0.134	11.05	0.694 ± 0.268	84.23	3.16 ± 1.063	18.49	58.44 ± 22.87
**20**	0.087 ± 0.059	47.82	0.087 ± 0.047	47.95	0.764 ± 0.335	5.44	4.16 ± 0.039
**21**	4.70 ± 1.185	22.06			8.72 ± 4.200	11.89	103.7 ± 26.99
**22**	0.406 ± 0.015	16.58			0.107 ± 0.049	62.90	6.73 ± 3.076
**23**	0.607 ± 0.010	26.33	0.570 ± 0.012	28.04	3.38 ± 0.009	4.73	15.98 ± 1.241
**24**	3.79 ± 0.163	16.31			1.45 ± 0.116	42.63	61.81 ± 27.87
PYR	0.017 ^f^		10.00				
CQ	0.0039	23313	0.163	558.4			90.92
MEL					0.010	778.0	7.78
POD							0.012

PYR = pyrimethamine; CQ = chloroquine; MEL = melarsoprol; POD = podophyllotoxin. ^a^ Values represent the average of four determinations (two determinations of two independent experiments). ^b^ sensitive to chloroquine and pyrimethamine. ^c^ resistant to chloroquine, sulfadoxine and pyrimethamine. ^d^ Ref. [12]. ^e^ Ref. [13]. ^f^ Ref. [24].

## Data Availability

The data presented in this study are available in this article.

## Supplementary Materials

The following are available online at https://www.mdpi.com/article/10.3390/molecules28010307/s1: ^1^H-, ^13^C-NMR and MS-spectra; synthesis route of compounds **1**–**4**.

## Sample Availability

Samples of selected compounds are not available from the authors.
