# Supplementary material for: Synthesis and Antiprotozoal Activity of Azabicyclo-Nonane Pyrimidine Hybrids
Source: Molecules. 2022 Dec 30;28(1):307. doi: 10.3390/molecules28010307 (PMC9821907; doi:10.3390/molecules28010307)
Supplement: Supplementary file 1 [file molecules-28-00307-s001.zip › molecules-2084372-supplementary.pdf]

Supplementary material

Synthesis and Antiprotozoal Activity of Azabicyclo-nonane Pyrimidine Hybrids

Clemens Hinteregger [1], Johanna Dolensky [1], Werner Seebacher [1], Robert Saf [2], Pascal Mäser [3,4], Marcel Kaiser [3,4] and Robert Weis [1,*]

[1] Pharmaceutical Chemistry, Institute of Pharmaceutical Sciences, University of Graz, Schubertstraße 1, A-8010 Graz, Austria
[2] Institute for Chemistry and Technology of Materials (ICTM), Graz University of Technology, Stremayrgasse 9, A-8010 Graz, Austria
[3] Swiss Tropical and Public Health Institute, Kreuzstraße 2, CH-4123 Allschwil, Switzerland
[4] Swiss Tropical and Public Health Institute, University of Basel, Petersplatz 1, CH-4003 Basel, Switzerland
* Correspondence: robert.weis@uni-graz.at; Tel.: +43-316-380-5379

NMR and HRMS spectra of compounds 5 – 24
Synthesis route of compounds 1-4

5 H nmr

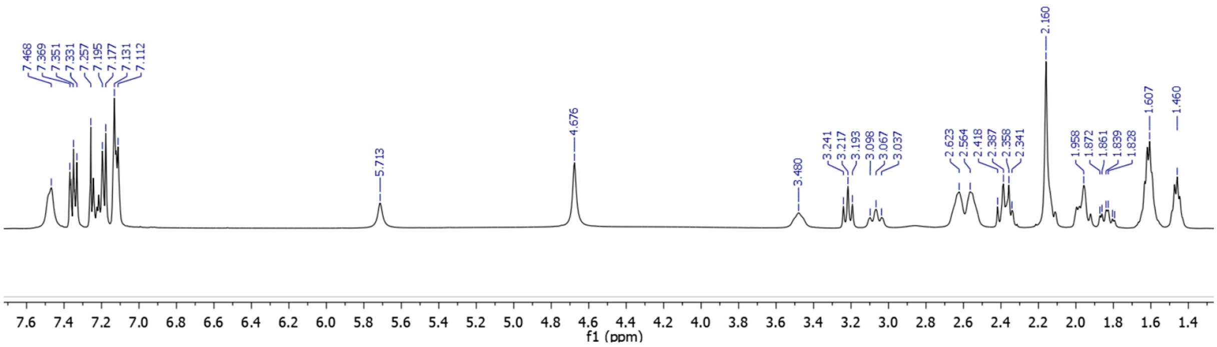
7.468
7.369
7.351
7.331
7.257
7.195
7.177
7.131
7.112
5.713
4.676
3.480
3.241
3.217
3.193
3.098
3.067
3.037
2.623
2.564
2.418
2.387
2.358
2.341
2.160
1.958
1.872
1.861
1.839
1.828
1.607
1.460
f1 (ppm)

5 C nmr

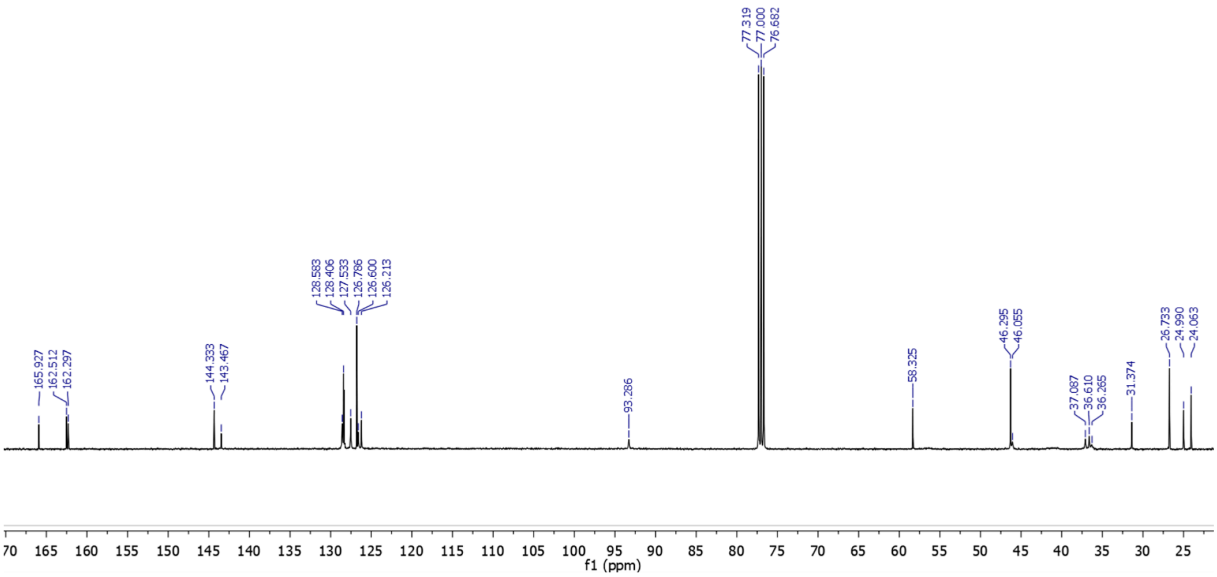
165.927
162.512
162.297
144.333
143.467
128.583
128.406
127.533
126.786
126.600
126.213
93.286
77.319
77.000
76.682
58.325
46.295
46.055
37.087
36.610
36.265
31.374
26.733
24.990
24.063
f1 (ppm)

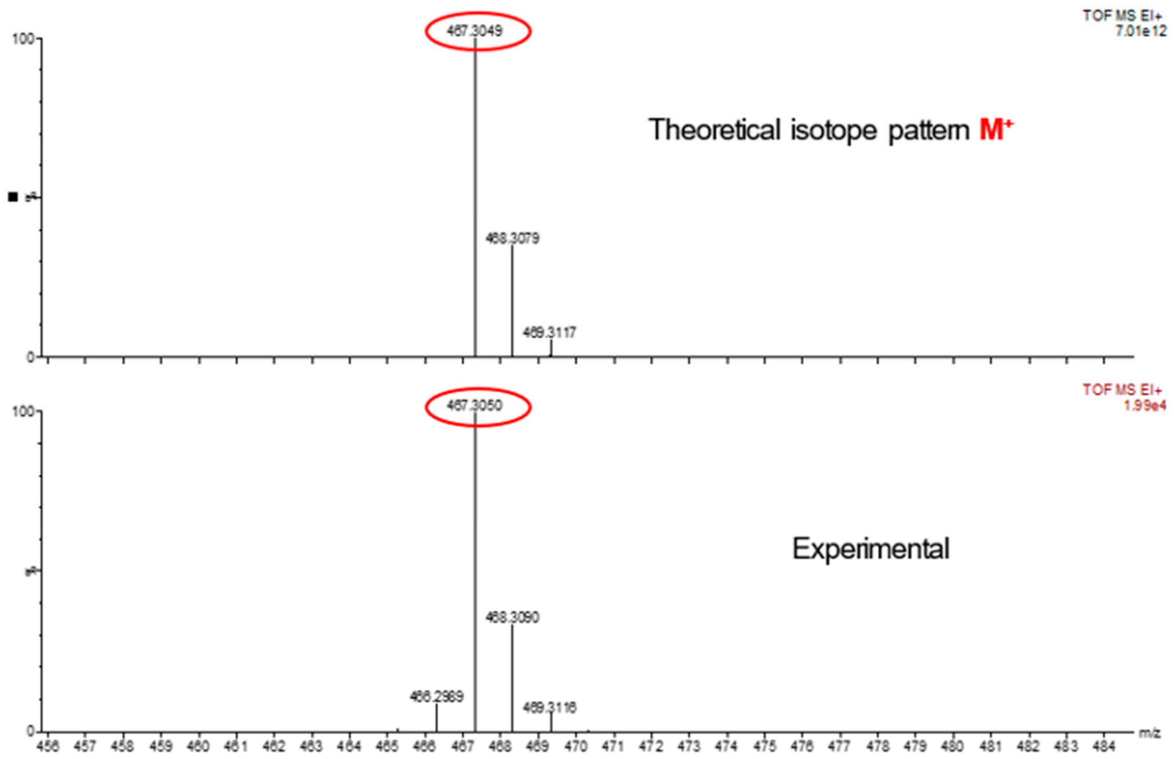
TOF MS EI+
7.01e12
467.3049
Theoretical isotope pattern M+
468.3079
469.3117
TOF MS EI+
1.99e4
467.3050
Experimental
468.3090
466.2989
469.3116
m/z

6 H nmr

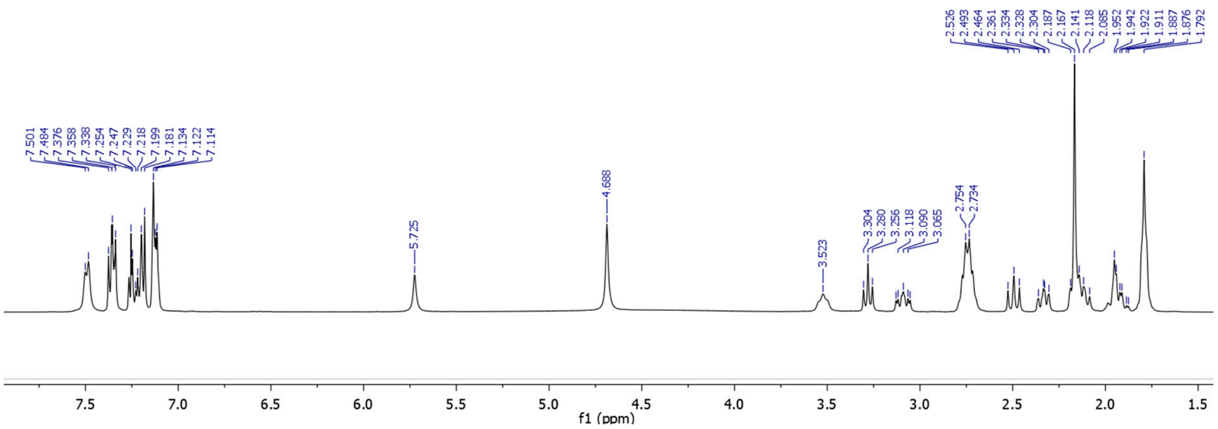

6 C nmr

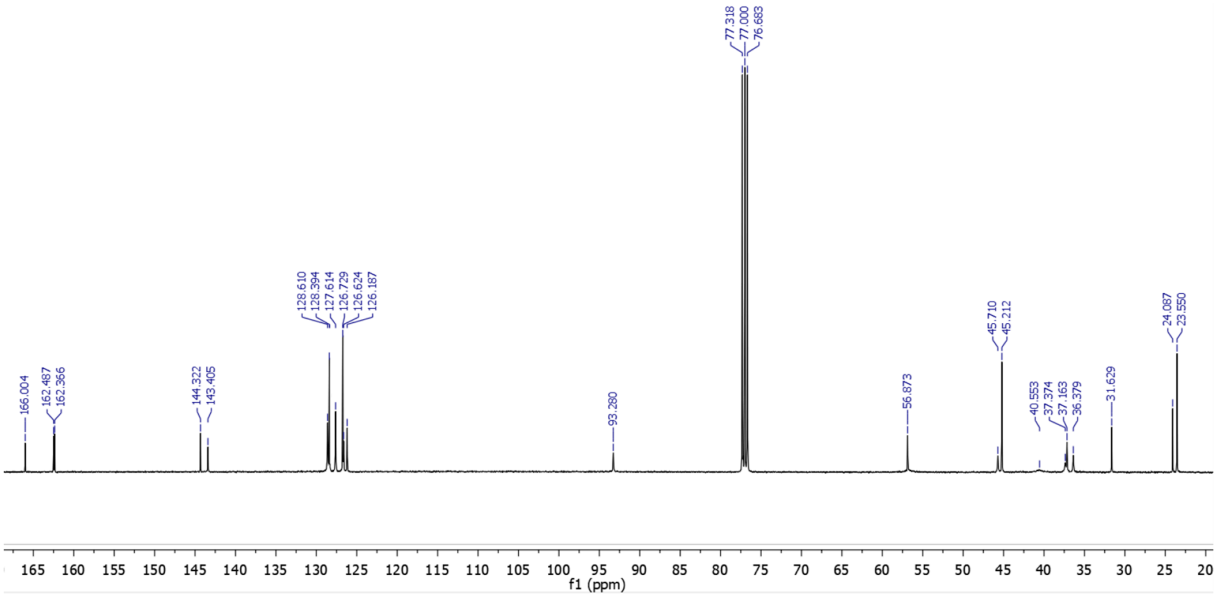

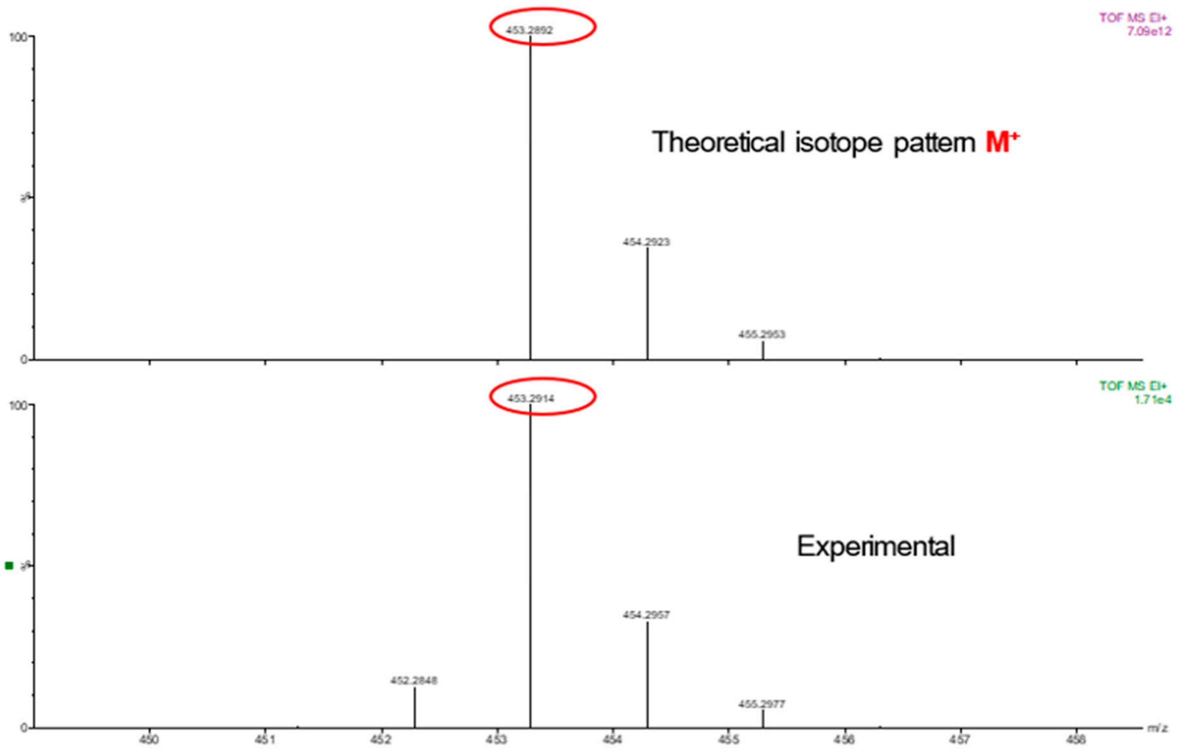

7 H nmr

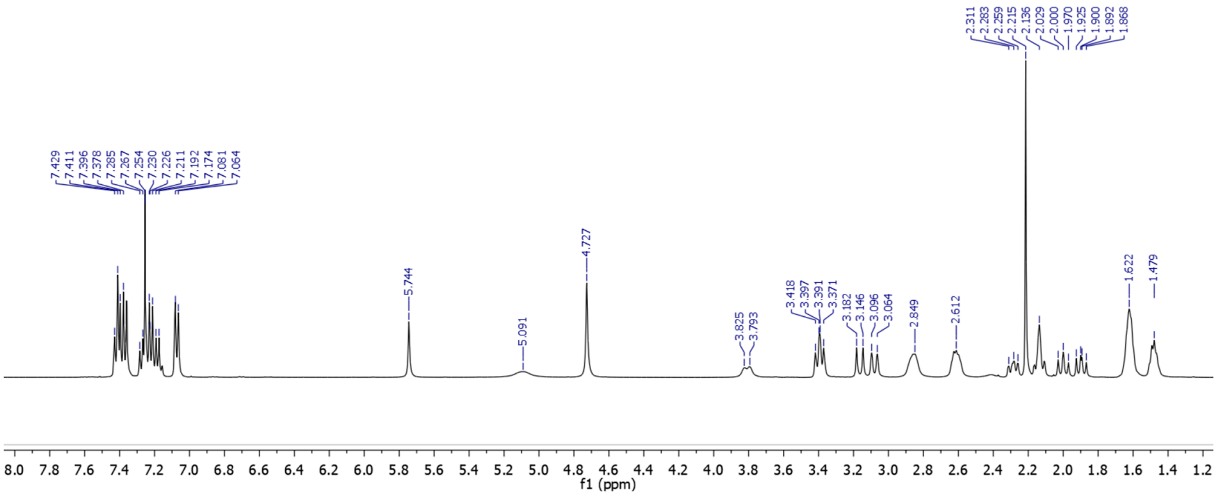

7.429
7.411
7.396
7.378
7.285
7.267
7.254
7.230
7.226
7.211
7.192
7.174
7.081
7.064
5.744
5.091
4.727
3.825
3.793
3.418
3.397
3.391
3.371
3.182
3.146
3.096
3.064
2.849
2.612
2.311
2.283
2.259
2.215
2.136
2.029
2.000
1.970
1.925
1.900
1.892
1.868
1.622
1.479
8.0 7.8 7.6 7.4 7.2 7.0 6.8 6.6 6.4 6.2 6.0 5.8 5.6 5.4 5.2 5.0 4.8 4.6 4.4 4.2 4.0 3.8 3.6 3.4 3.2 3.0 2.8 2.6 2.4 2.2 2.0 1.8 1.6 1.4 1.2
f1 (ppm)

7 C nmr

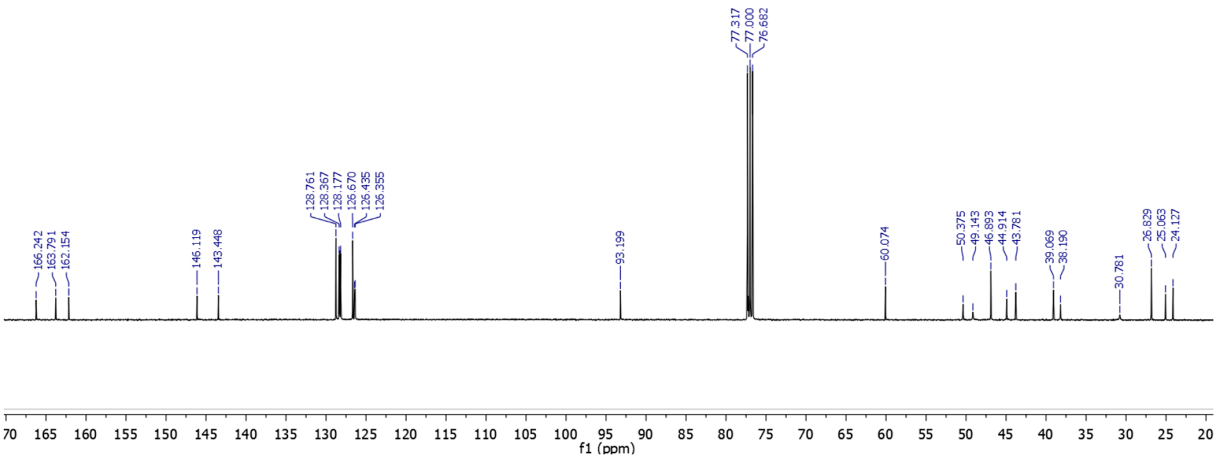

166.242
163.791
162.154
146.119
143.448
128.761
128.367
128.177
126.670
126.435
126.355
93.199
77.317
77.000
76.682
60.074
50.375
49.143
46.893
44.914
43.781
39.069
38.190
30.781
26.829
25.063
24.127
70 165 160 155 150 145 140 135 130 125 120 115 110 105 100 95 90 85 80 75 70 65 60 55 50 45 40 35 30 25 20
f1 (ppm)

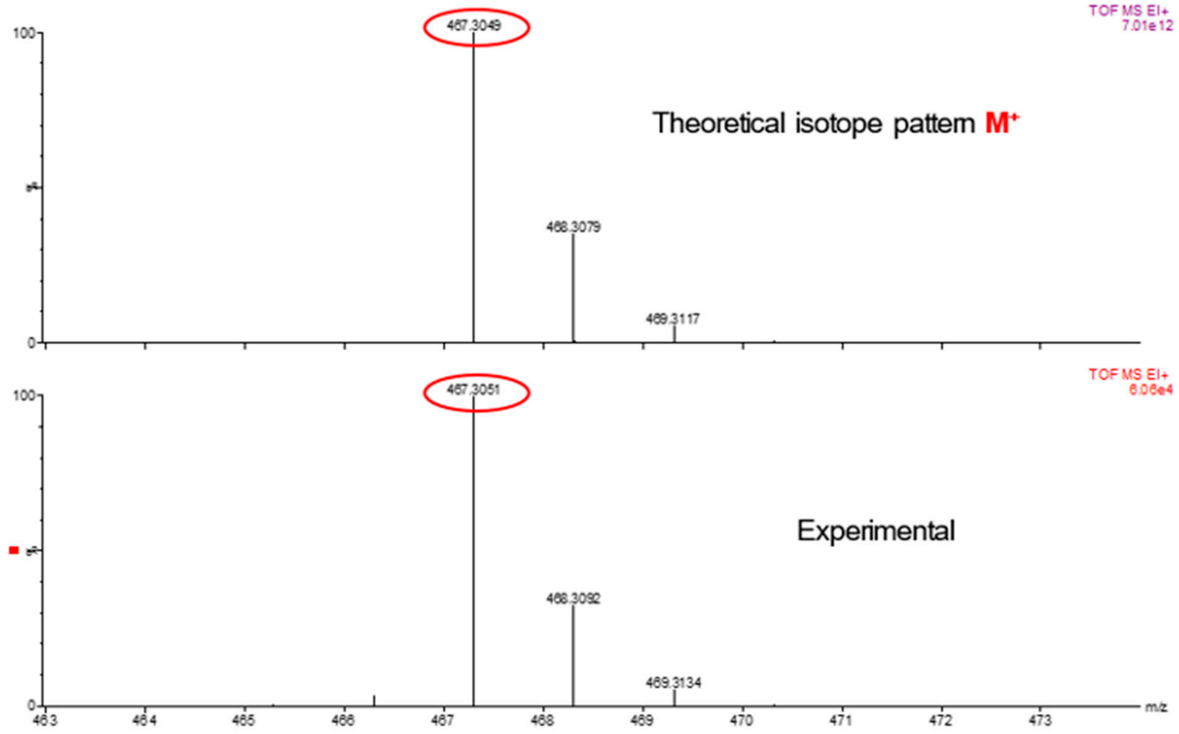

TOF MS EI+
7.01e12
467.3049
468.3079
469.3117
Theoretical isotope pattern M+
100
0
TOF MS EI+
6.06e4
467.3051
468.3092
469.3134
Experimental
100
0
463 464 465 466 467 468 469 470 471 472 473
m/z

8 H nmr

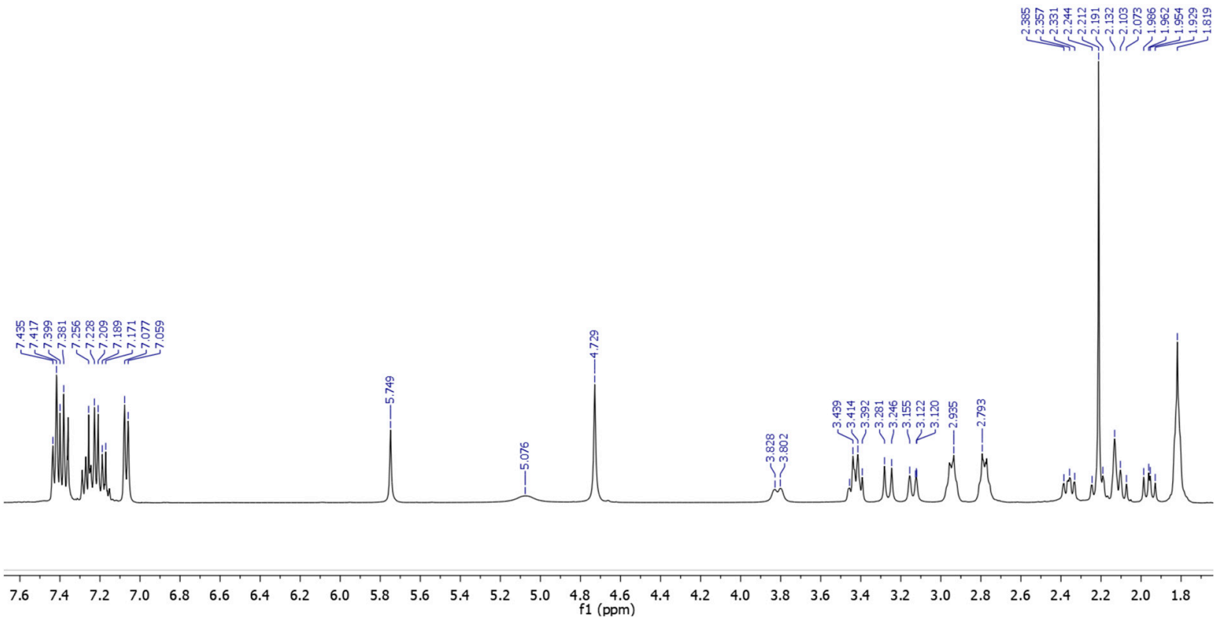
2.385
2.357
2.331
2.244
2.212
2.191
2.132
2.103
2.073
1.986
1.962
1.954
1.929
1.819
7.435
7.417
7.399
7.381
7.256
7.228
7.209
7.189
7.171
7.077
7.059
5.749
5.076
4.729
3.828
3.802
3.439
3.414
3.392
3.281
3.246
3.155
3.122
3.120
2.935
2.793
7.6 7.4 7.2 7.0 6.8 6.6 6.4 6.2 6.0 5.8 5.6 5.4 5.2 5.0 4.8 4.6 4.4 4.2 4.0 3.8 3.6 3.4 3.2 3.0 2.8 2.6 2.4 2.2 2.0 1.8
f1 (ppm)

8 C nmr

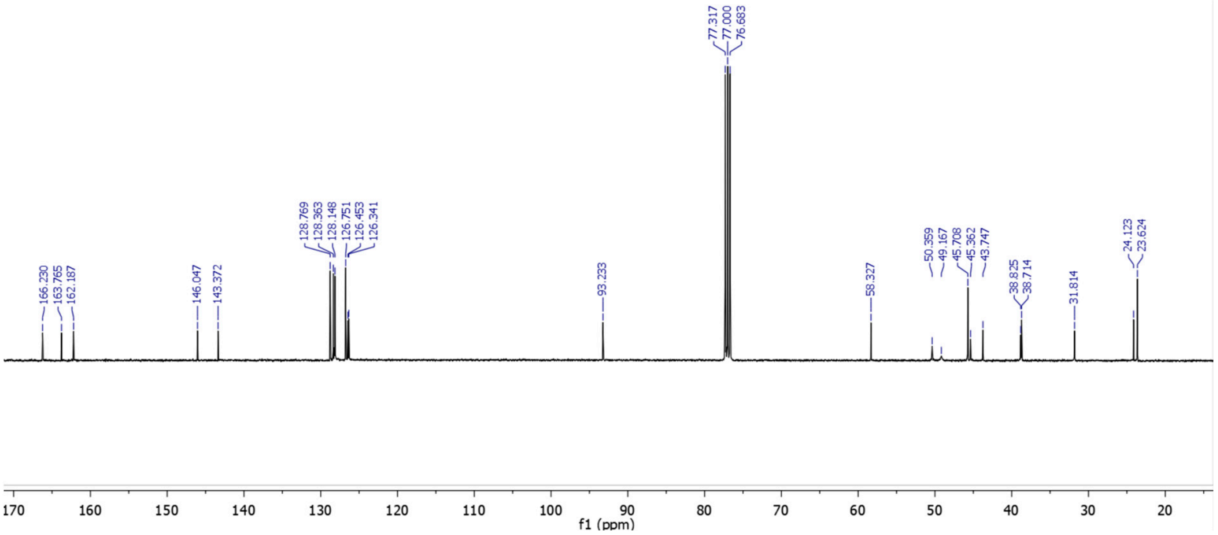
77.317
77.000
76.683
128.769
128.363
128.148
126.751
126.453
126.341
166.230
163.765
162.187
146.047
143.372
93.233
58.327
50.359
49.167
45.708
45.362
43.747
38.825
38.714
31.814
24.123
23.624
170 160 150 140 130 120 110 100 90 80 70 60 50 40 30 20
f1 (ppm)

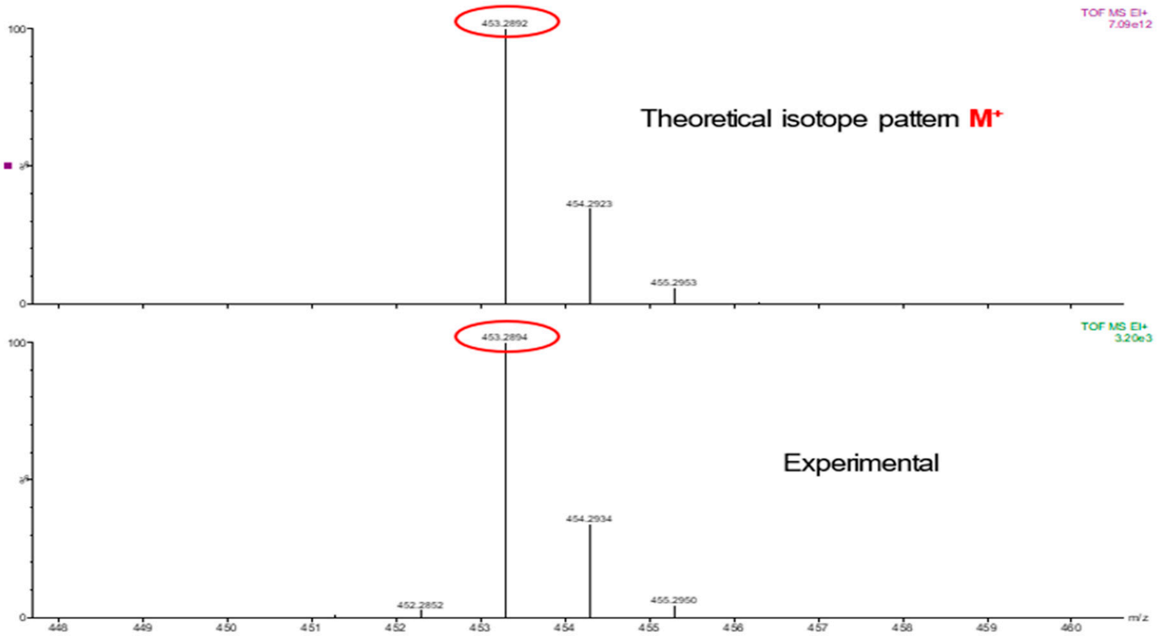
453.2892
TOF MS EI+
7.09e12
Theoretical isotope pattern M+
454.2923
455.2953
453.2894
TOF MS EI+
3.20e3
Experimental
454.2934
452.2852
455.2950
448 449 450 451 452 453 454 455 456 457 458 459 460
m/z

9 H nmr

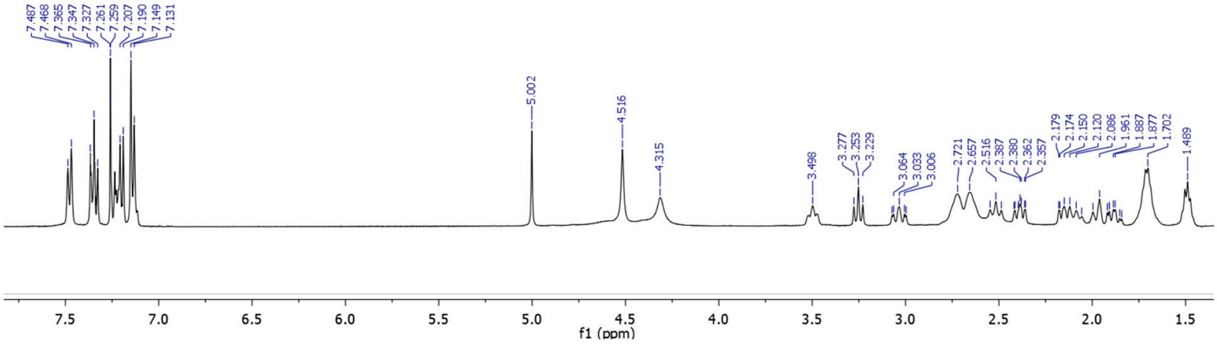

7.487
7.468
7.365
7.347
7.327
7.261
7.259
7.207
7.190
7.149
7.131
5.002
4.516
4.315
3.498
3.277
3.253
3.229
3.064
3.033
3.006
2.721
2.657
2.516
2.387
2.380
2.362
2.357
2.179
2.174
2.150
2.120
2.086
1.961
1.887
1.877
1.702
1.489
7.5 7.0 6.5 6.0 5.5 5.0 4.5 4.0 3.5 3.0 2.5 2.0 1.5
f1 (ppm)

9 C nmr

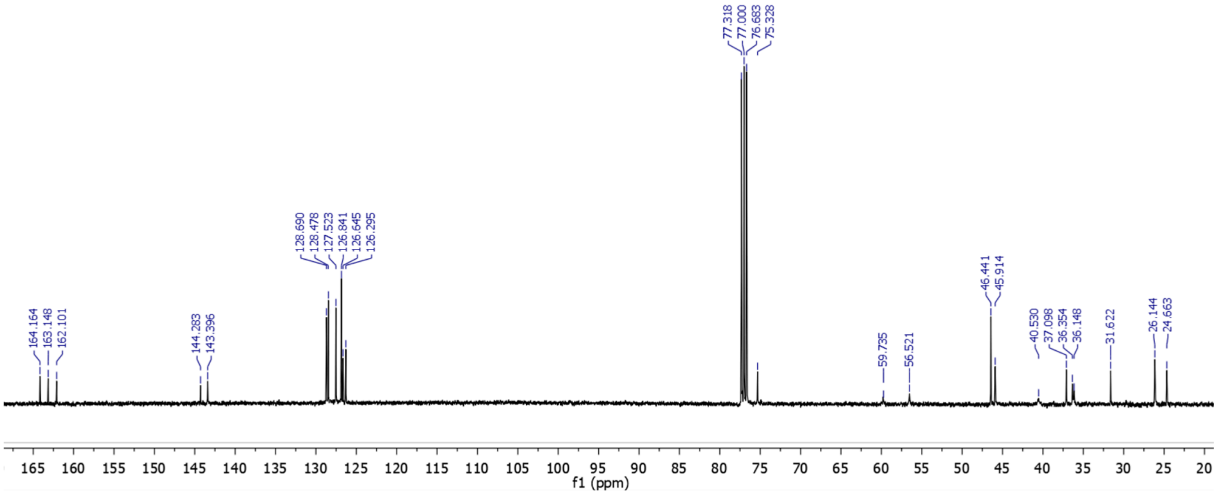

164.164
163.148
162.101
144.283
143.396
128.690
128.478
127.523
126.841
126.645
126.295
77.318
77.000
76.683
75.328
59.735
56.521
46.441
45.914
40.530
37.098
36.354
36.148
31.622
26.144
24.663
165 160 155 150 145 140 135 130 125 120 115 110 105 100 95 90 85 80 75 70 65 60 55 50 45 40 35 30 25 20
f1 (ppm)

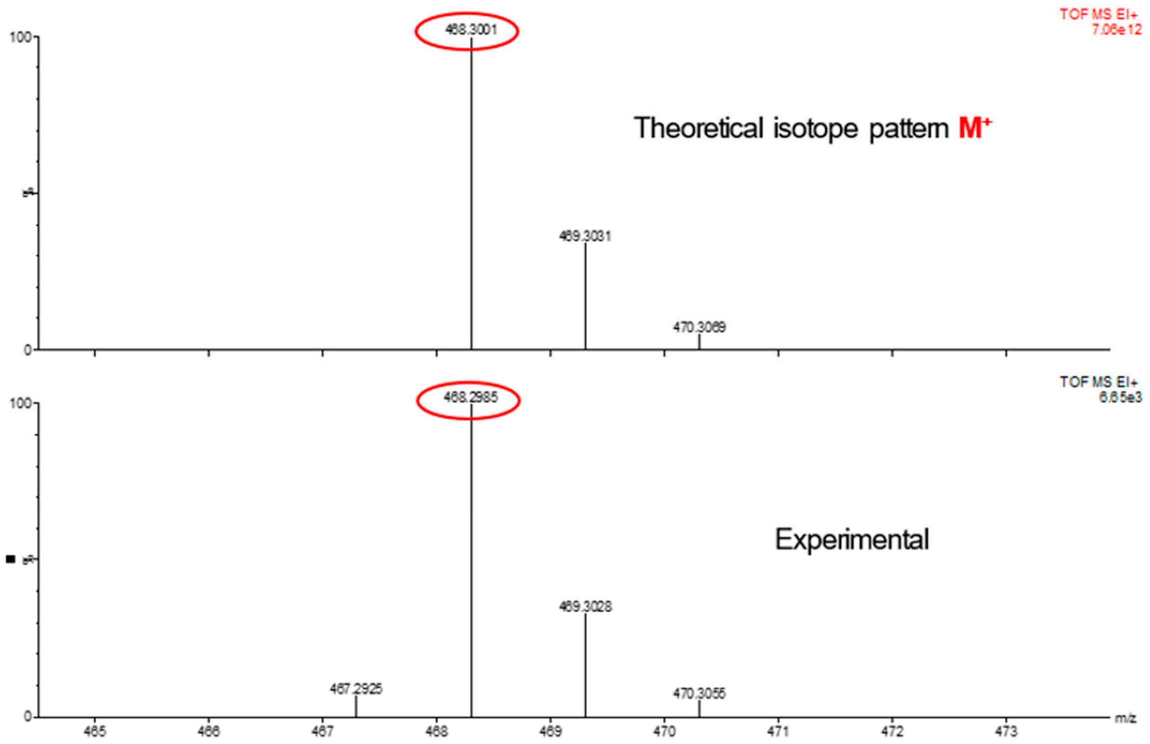

TOF MS EI+
7.06e12
Theoretical isotope pattern M+
468.3001
469.3031
470.3069
100
0
TOF MS EI+
6.65e3
Experimental
468.2985
469.3028
467.2925
470.3055
100
0
465 466 467 468 469 470 471 472 473
m/z

10 H nmr

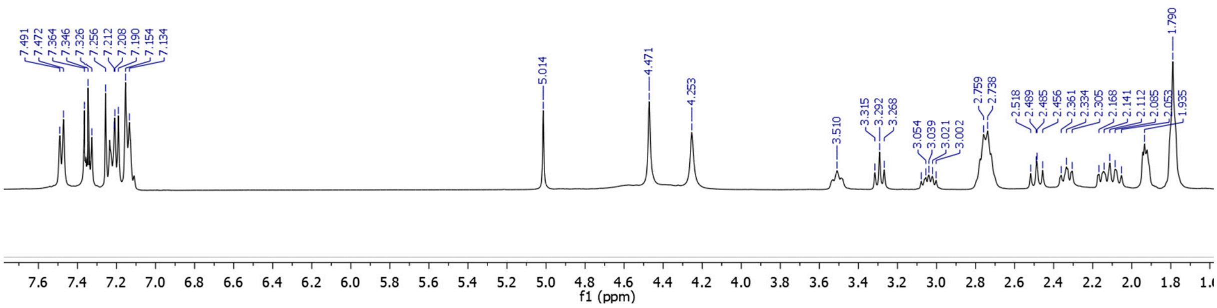

10 C nmr

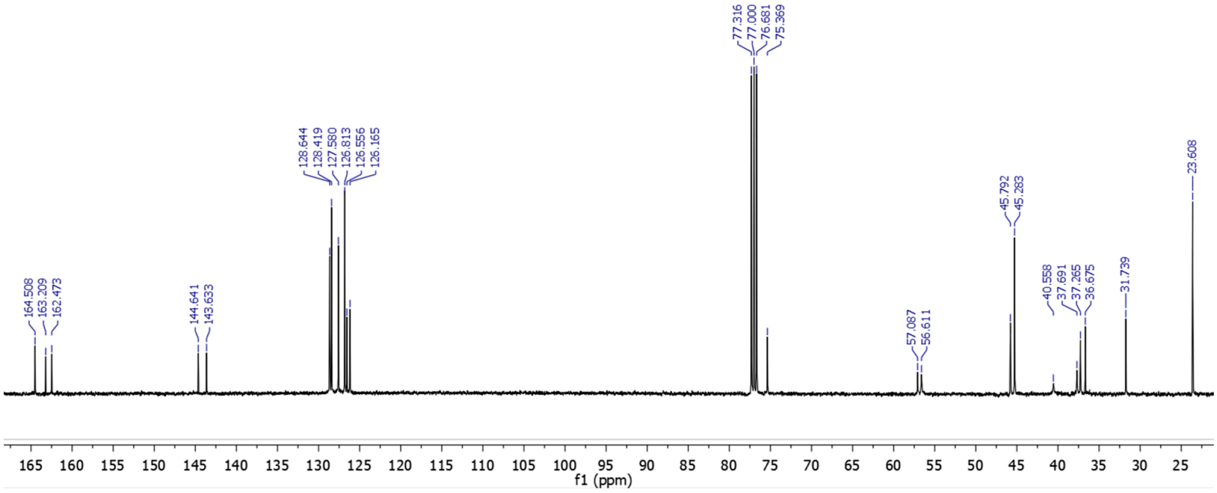

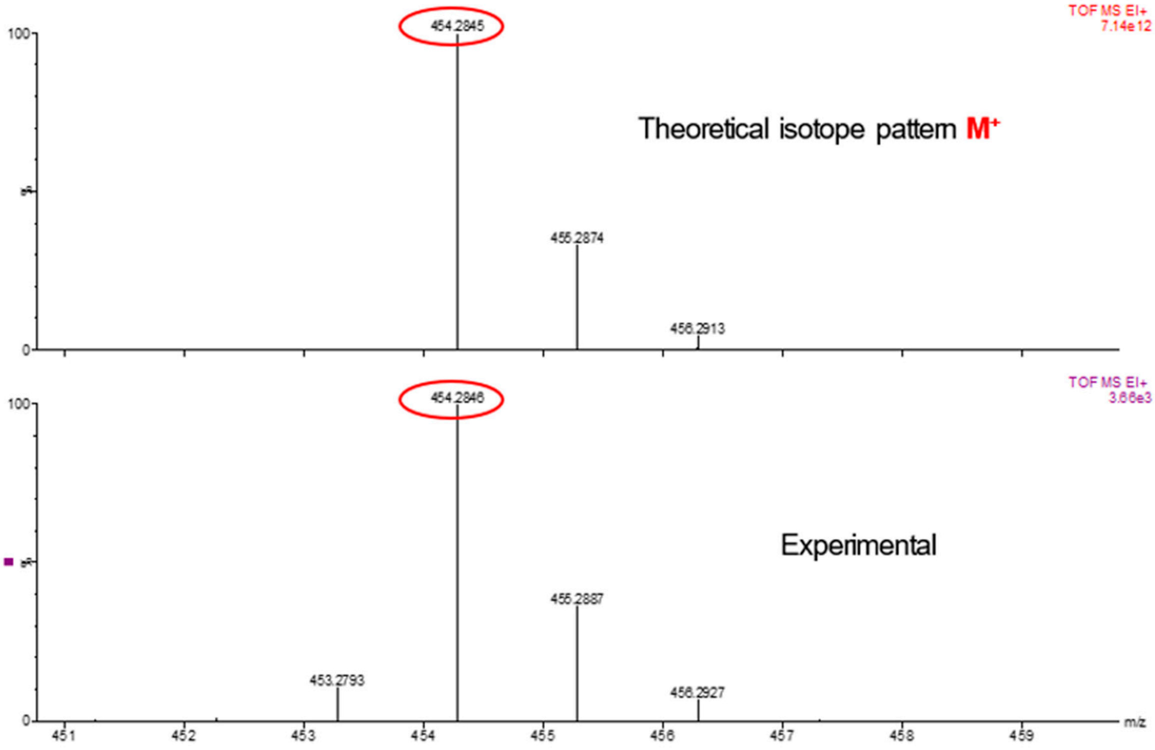

11 H nmr

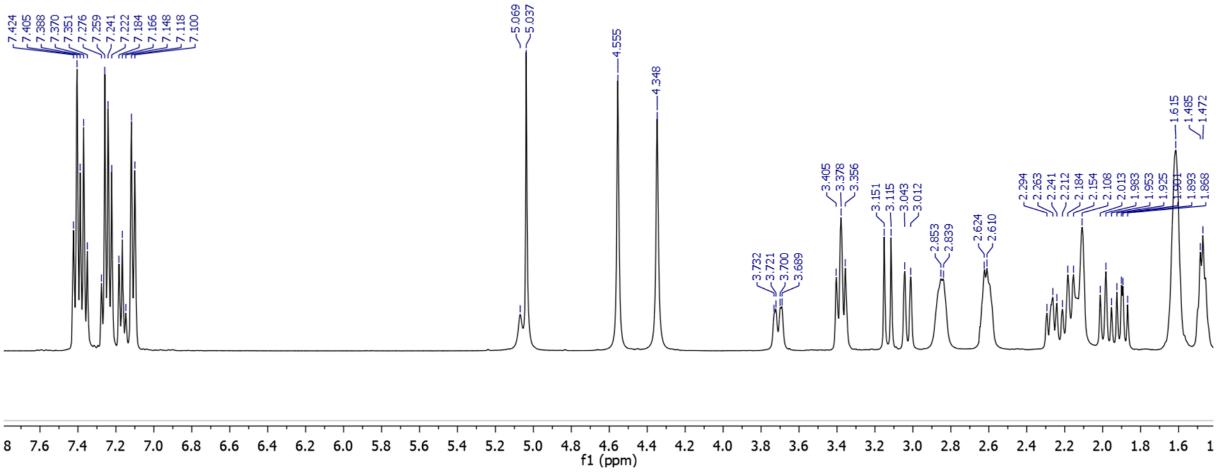

11 C nmr

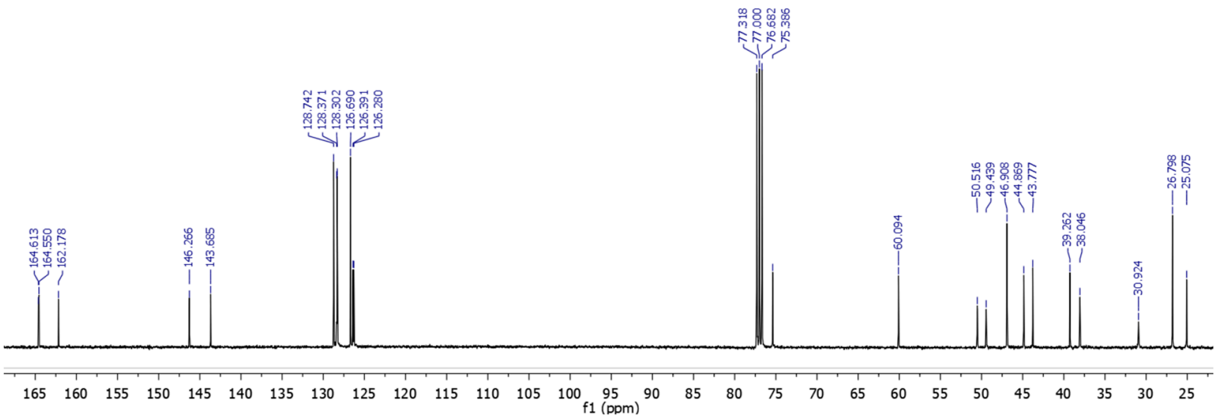

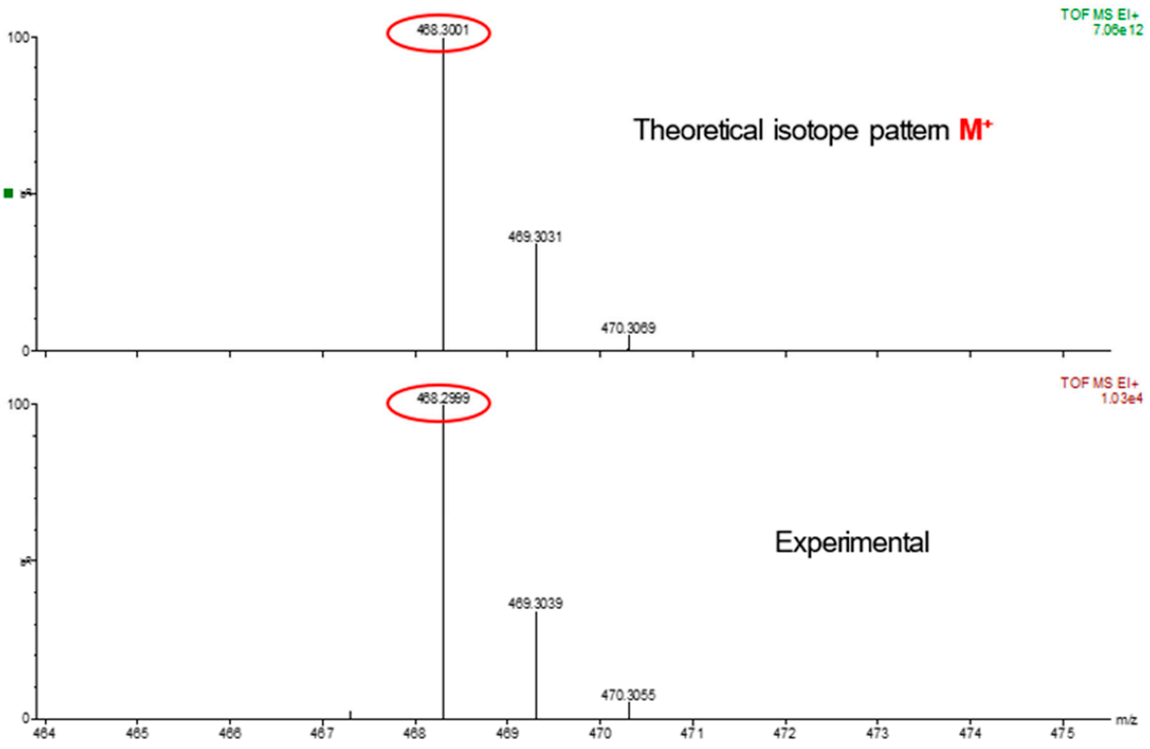

12 H nmr

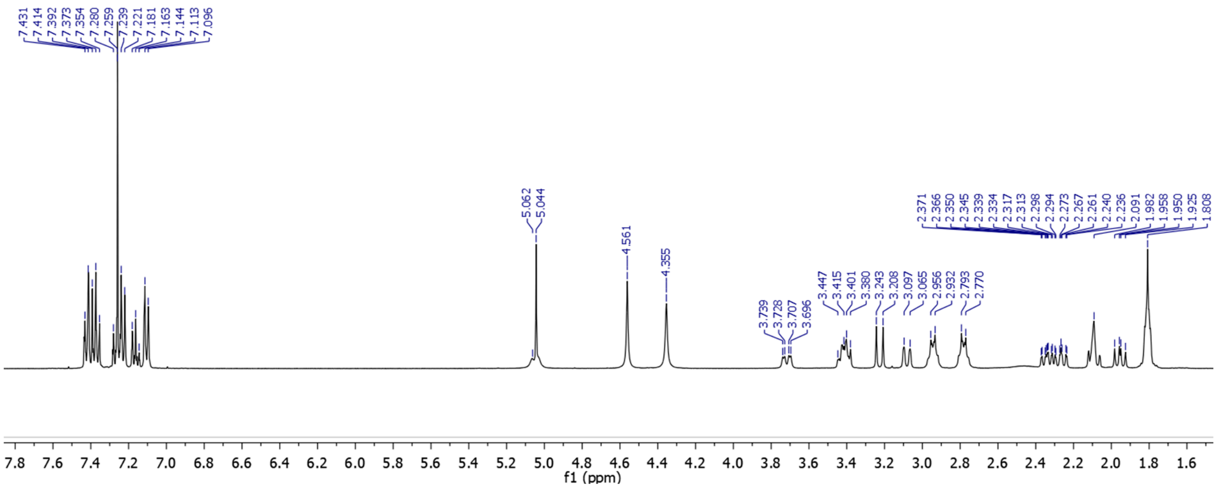

12 C nmr

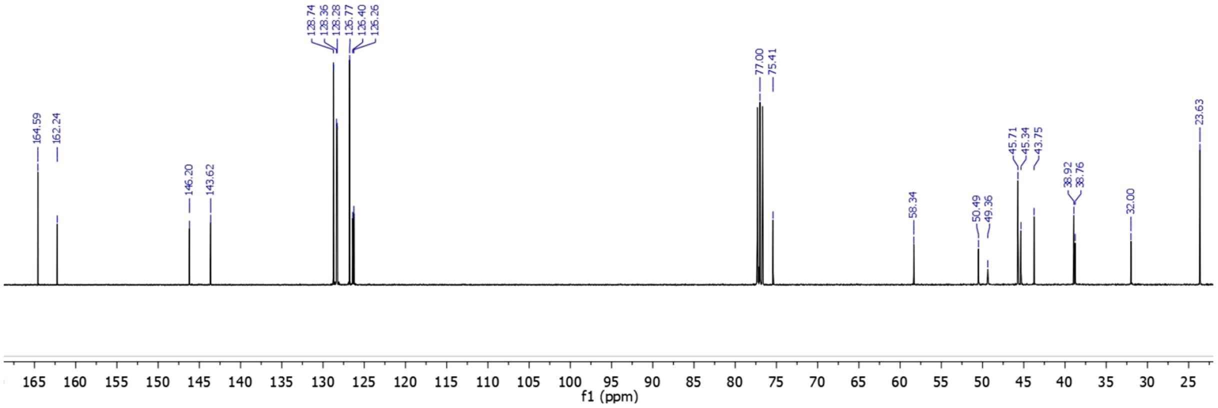

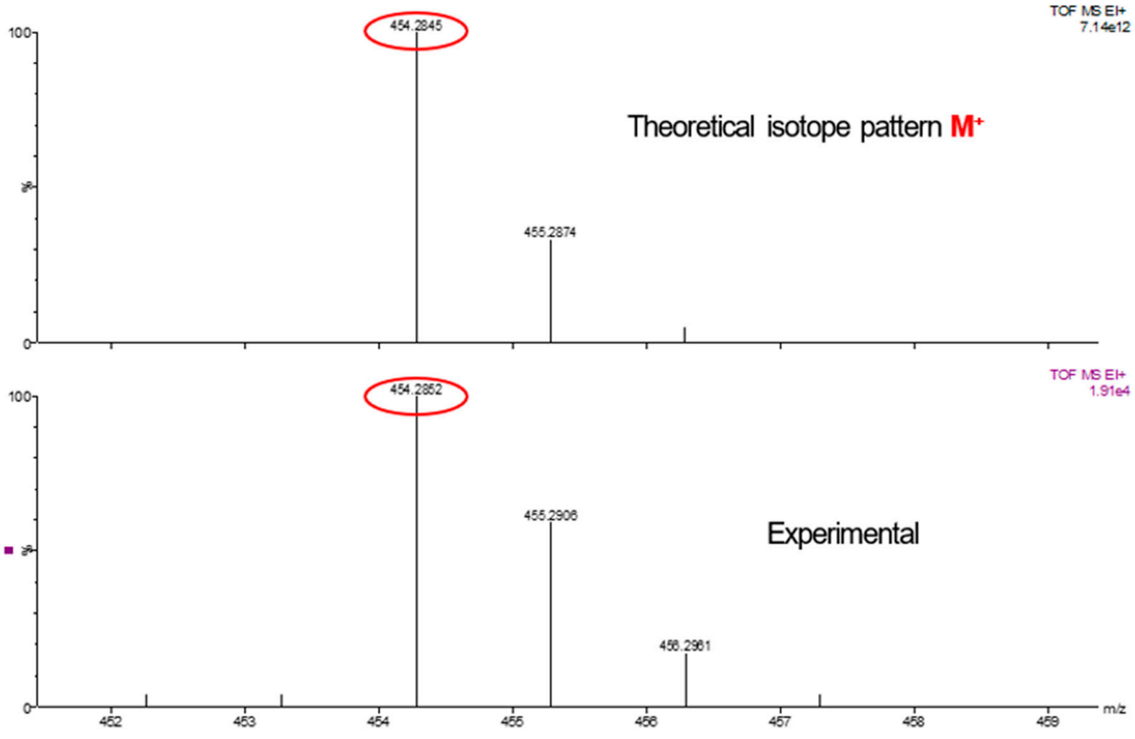

13 H nmr

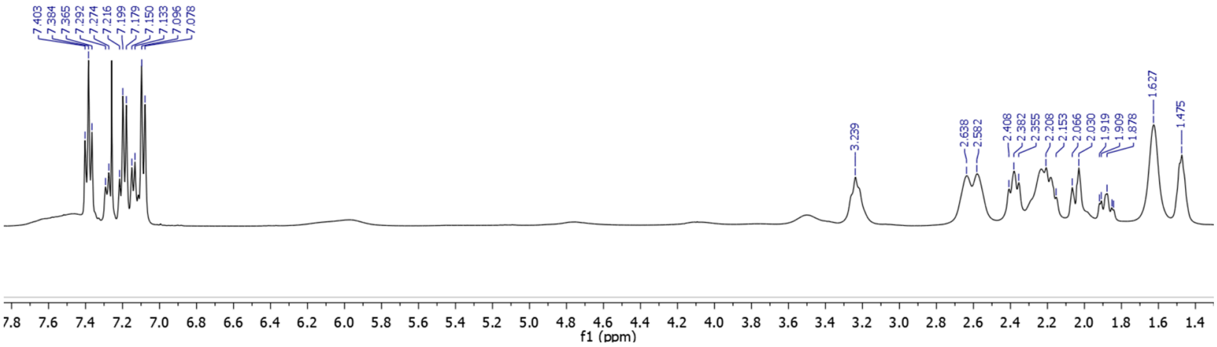

13 C nmr

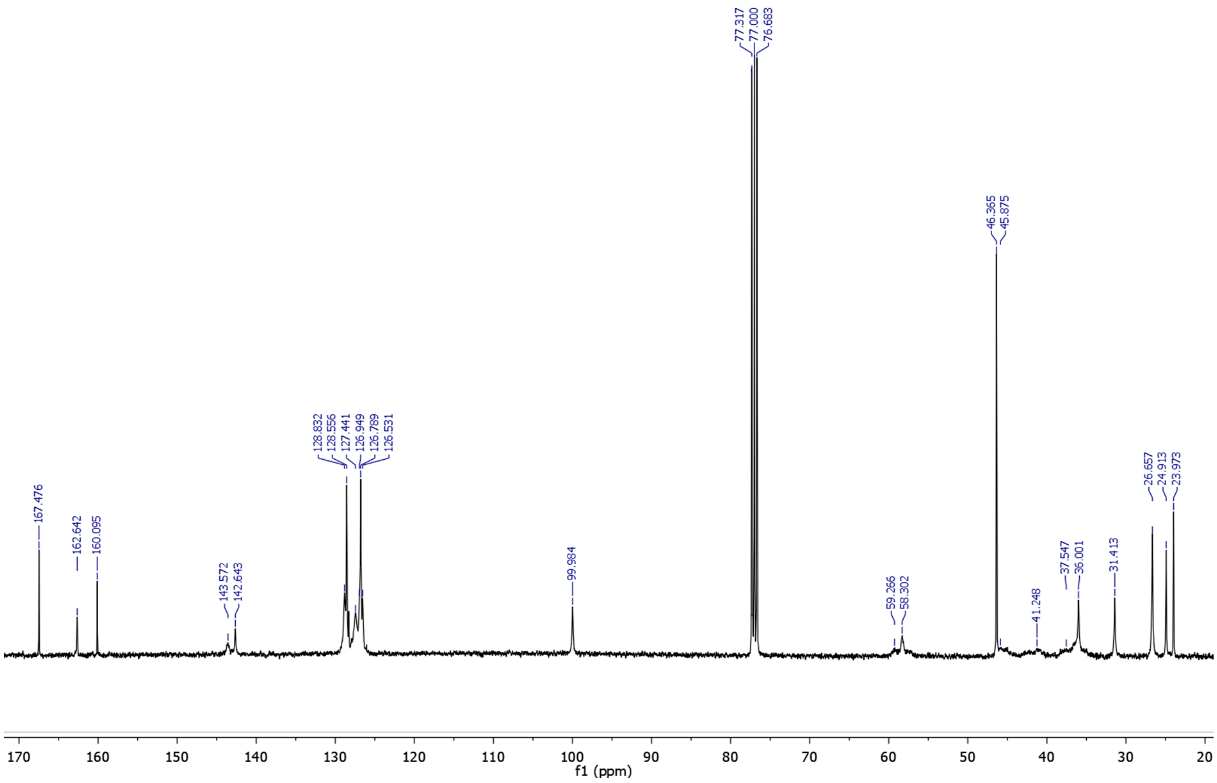

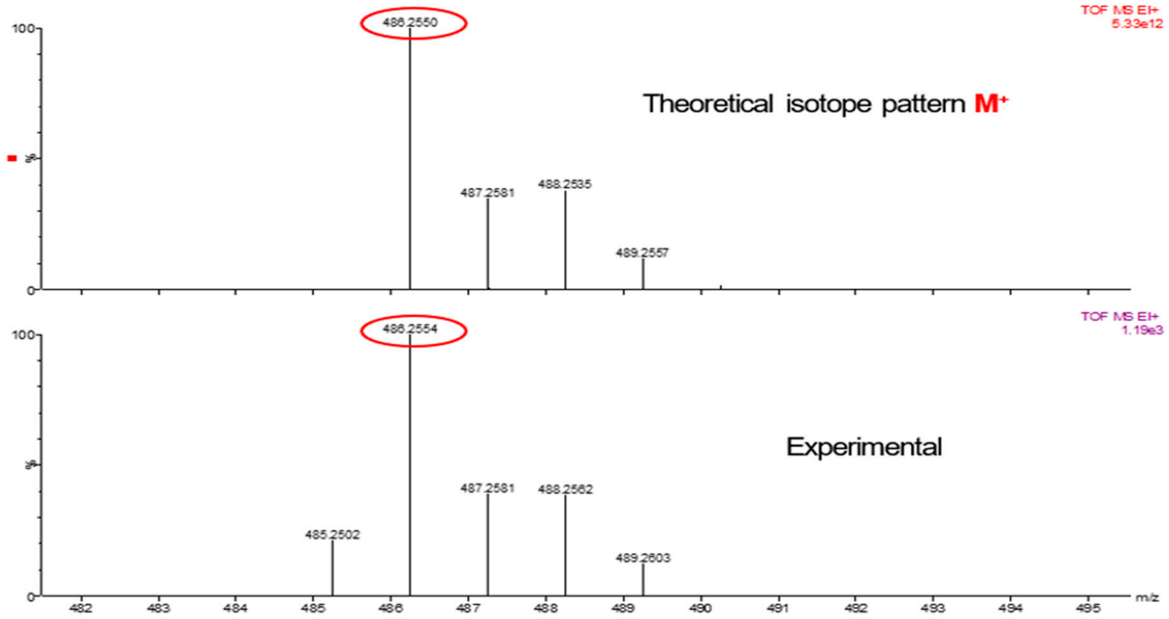

14 H nmr

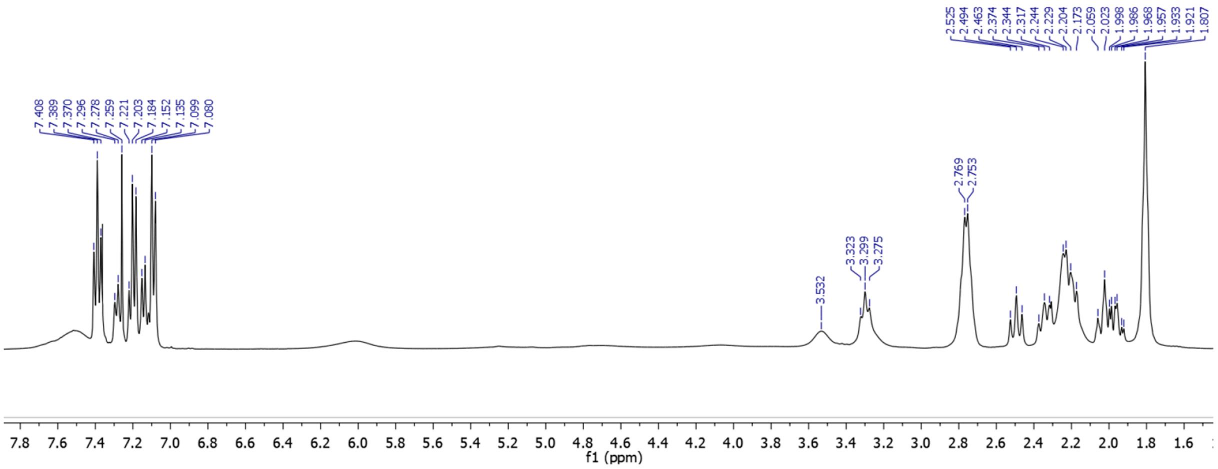

14 C nmr

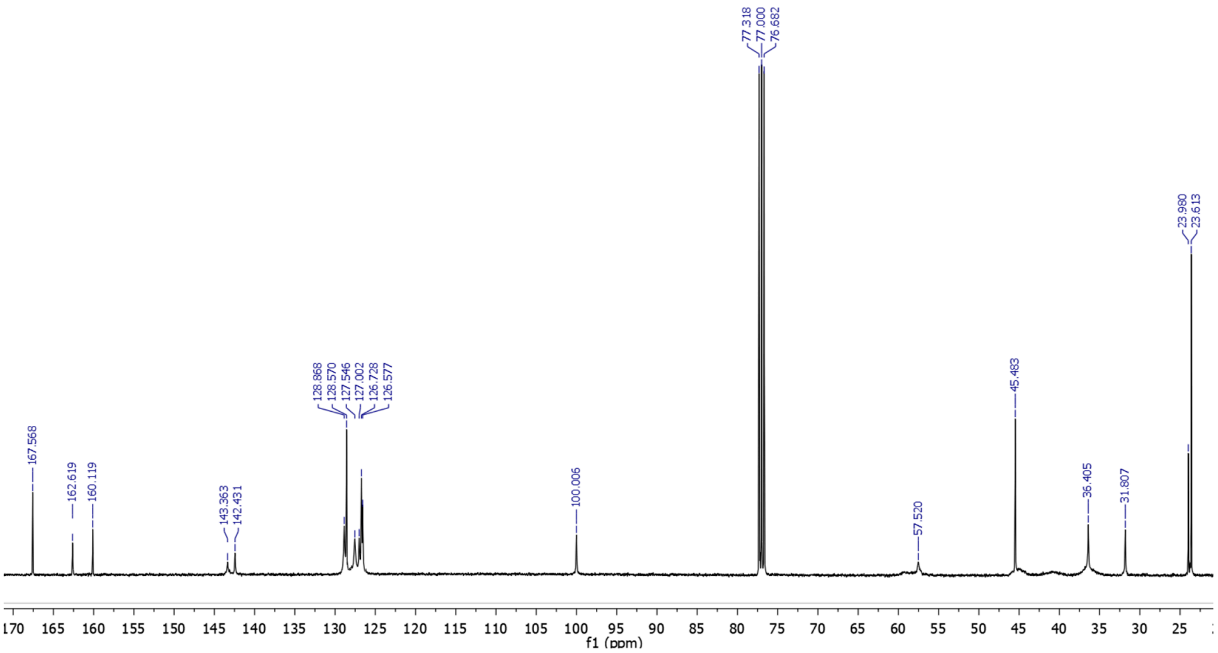

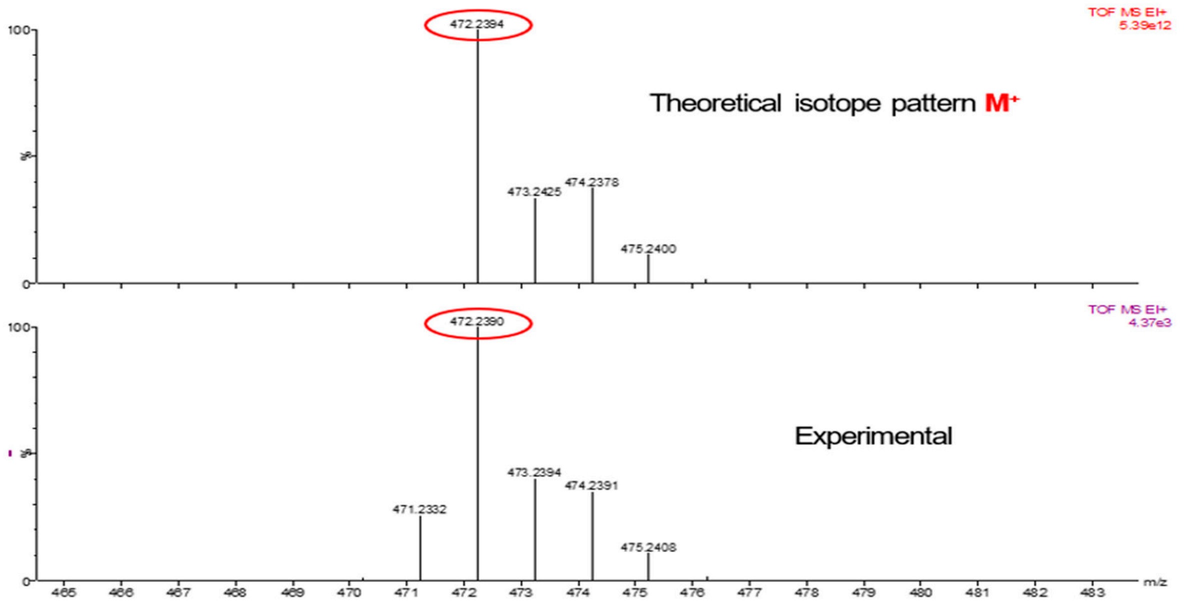

15 H nmr

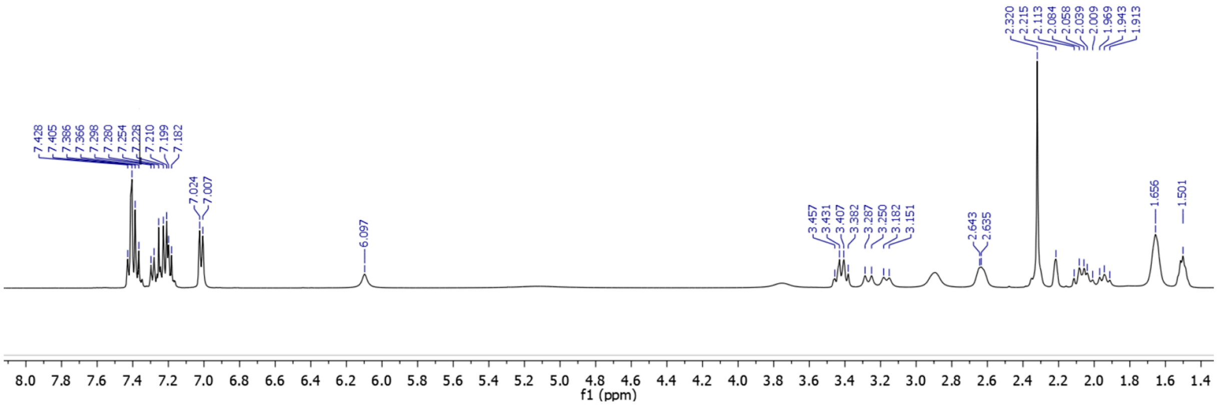

15 C nmr

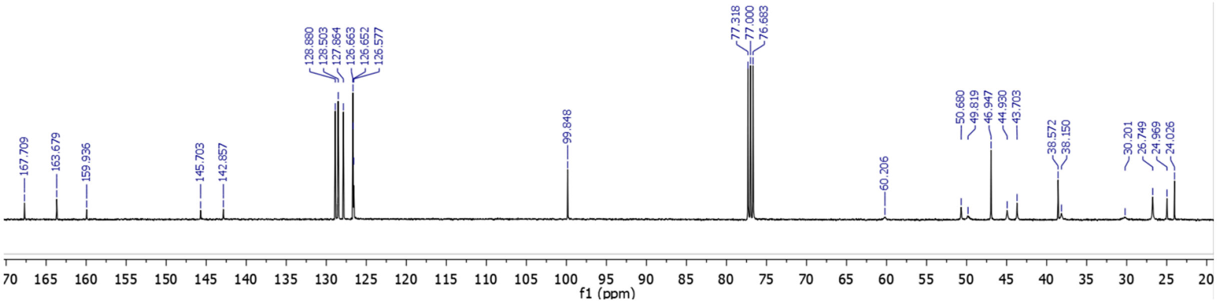

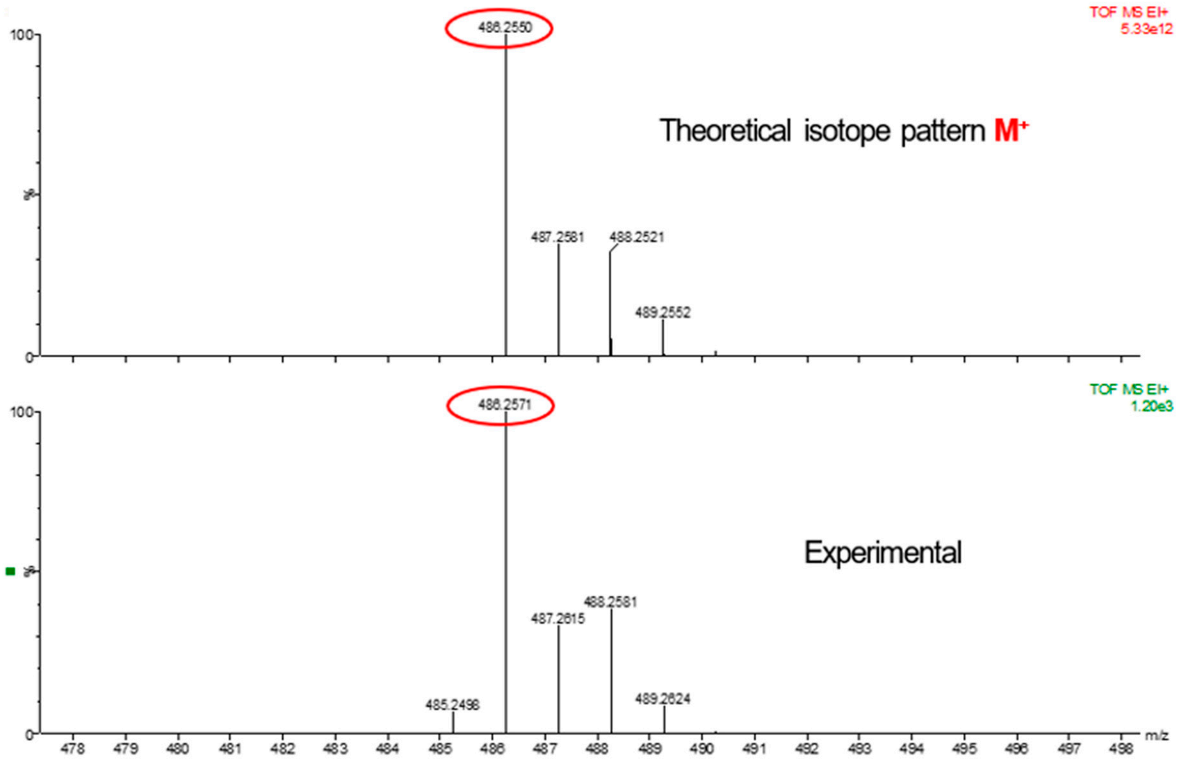

16 H nmr

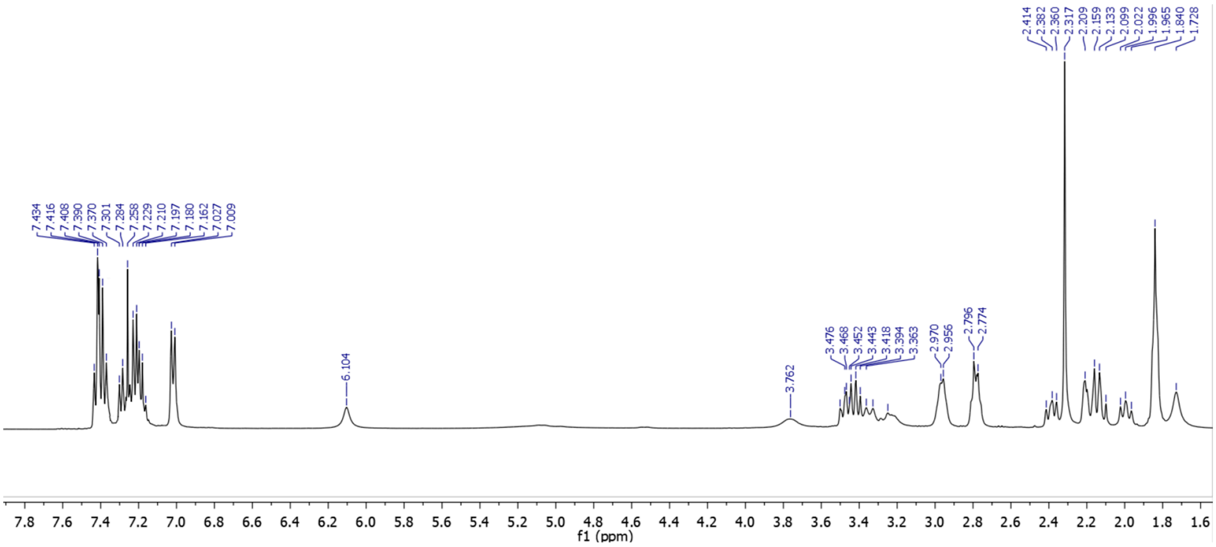

16 C nmr

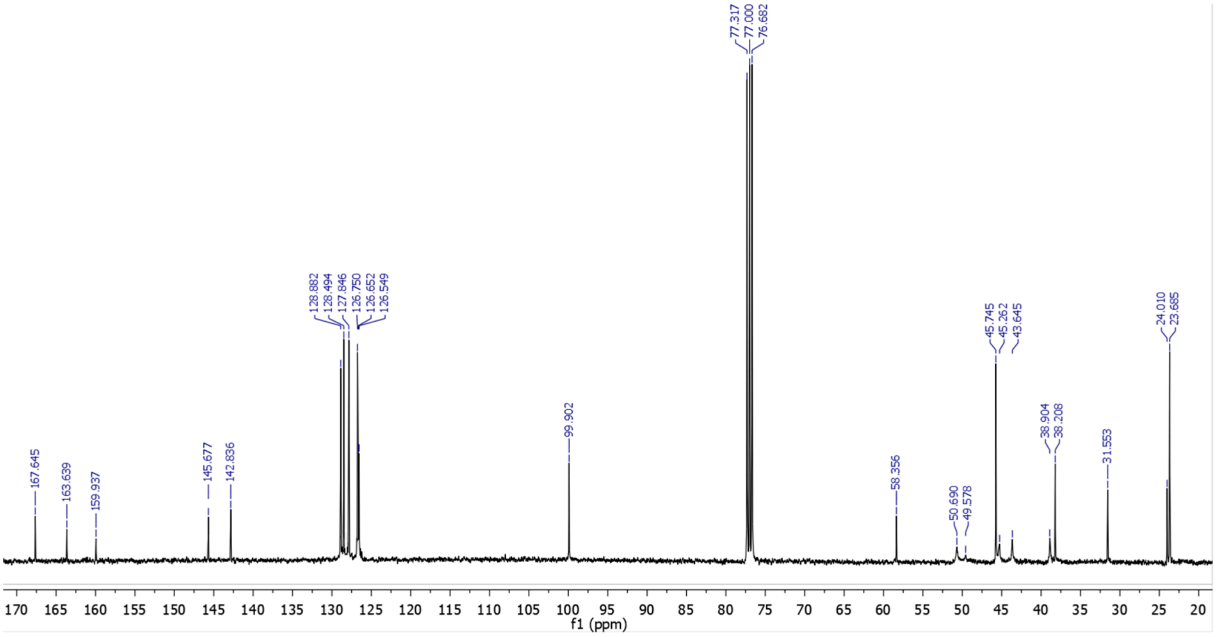

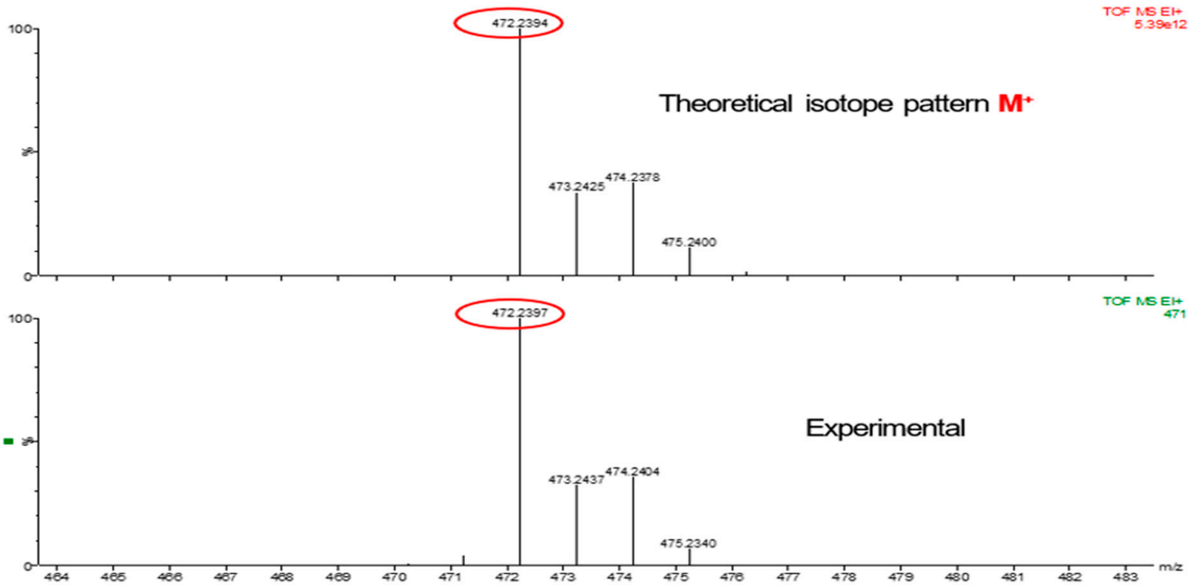

17 H nmr

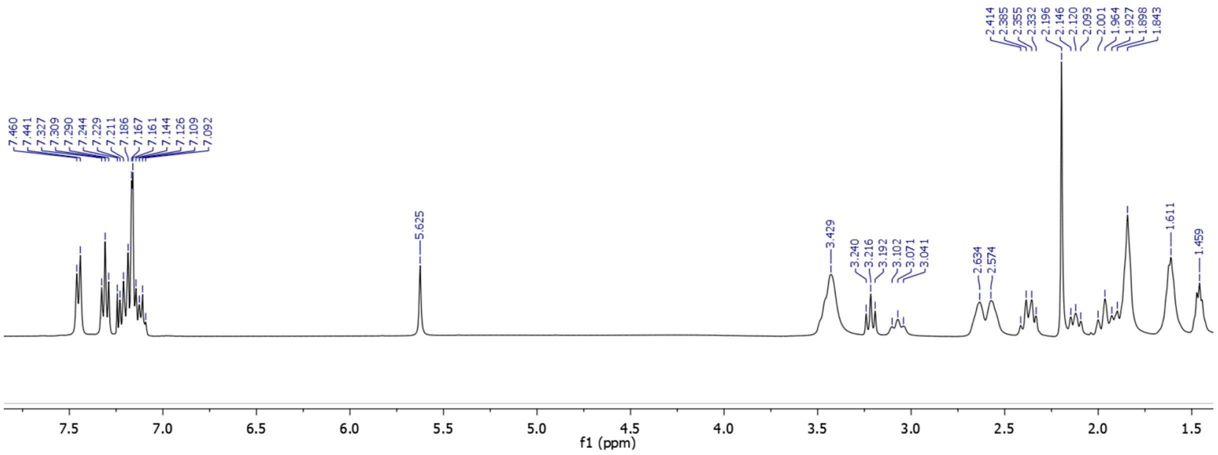

17 C nmr

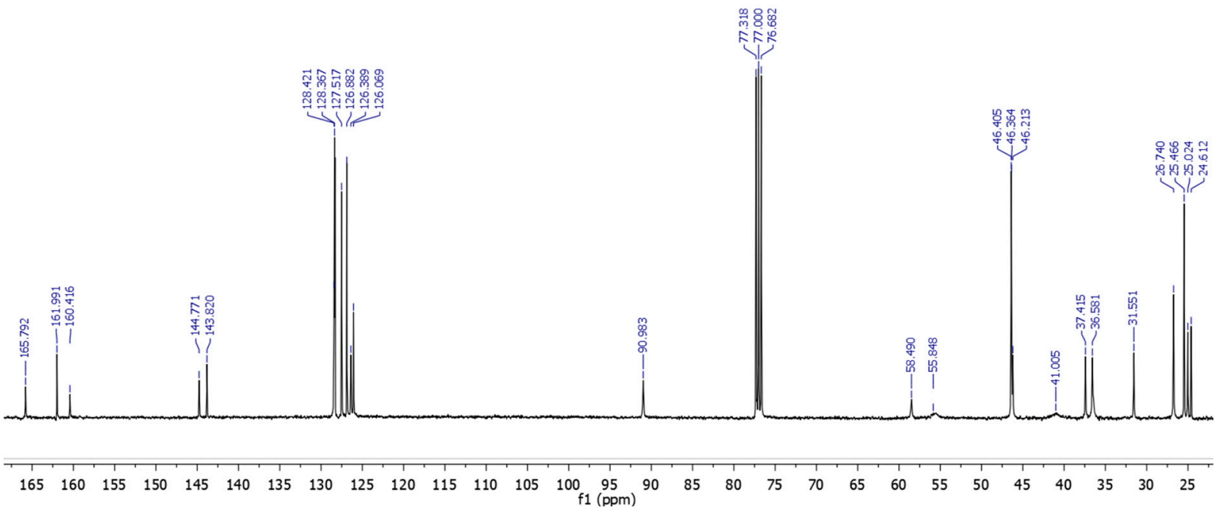

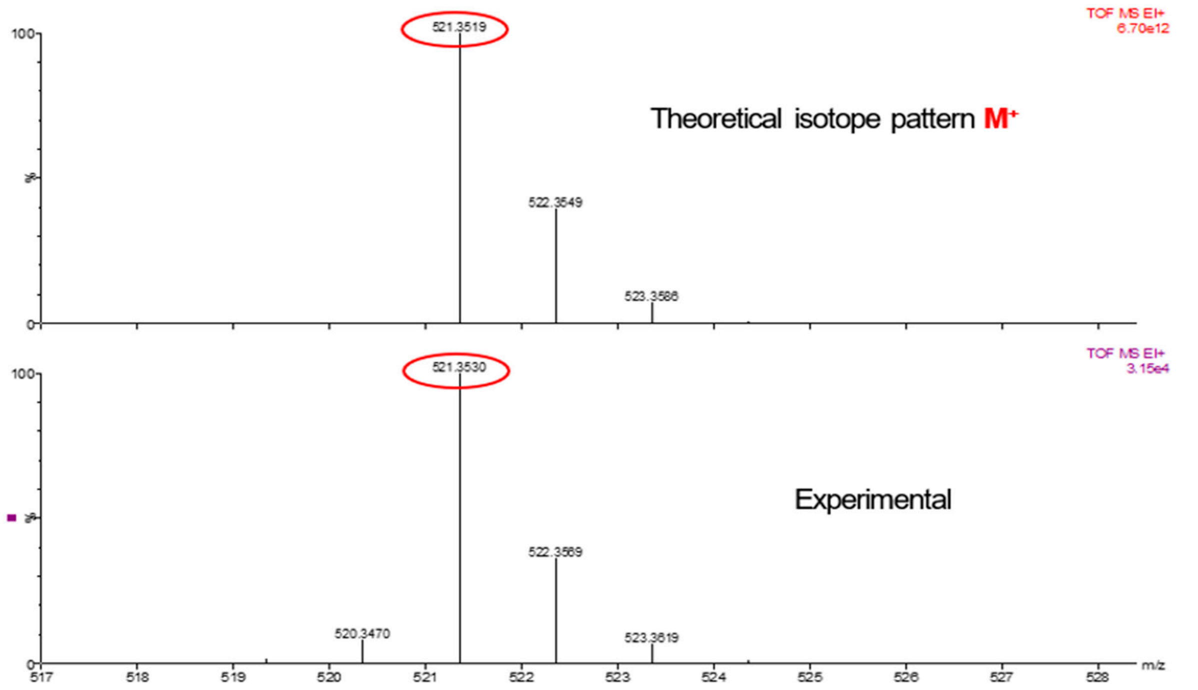

18 H nmr

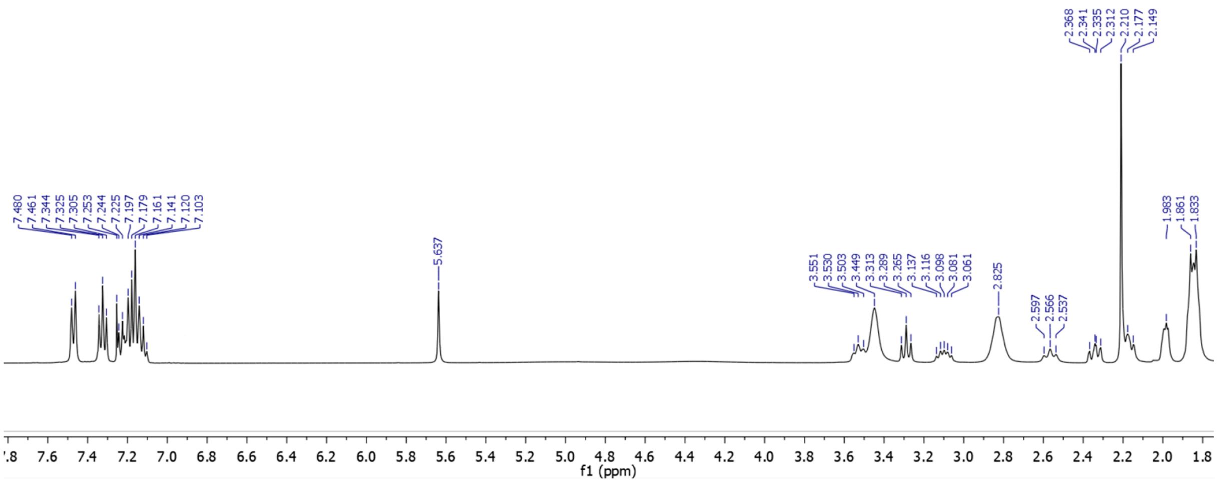

18 C nmr

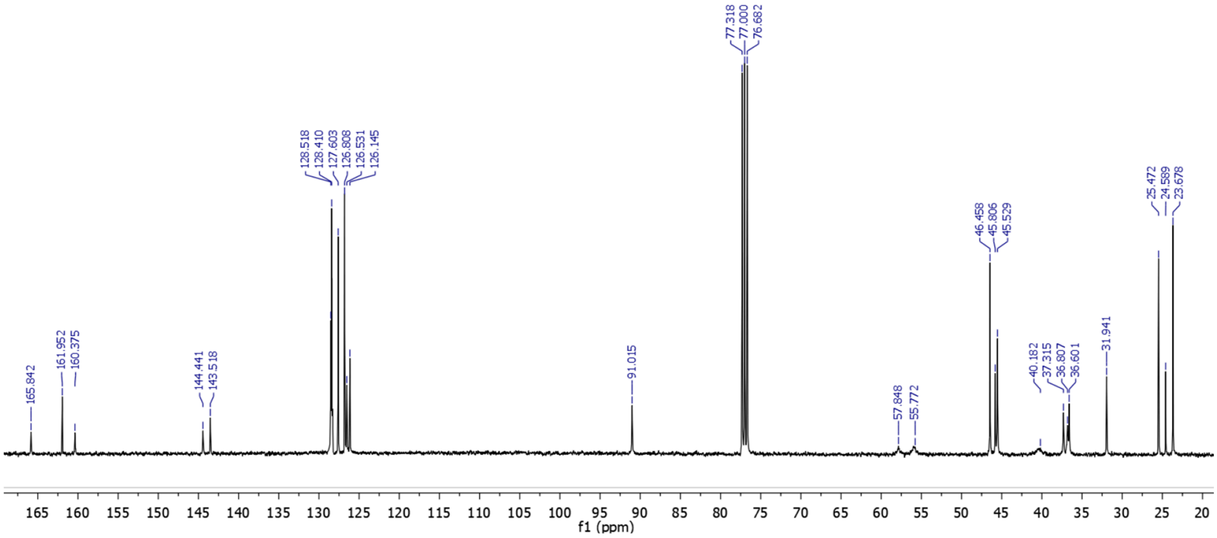

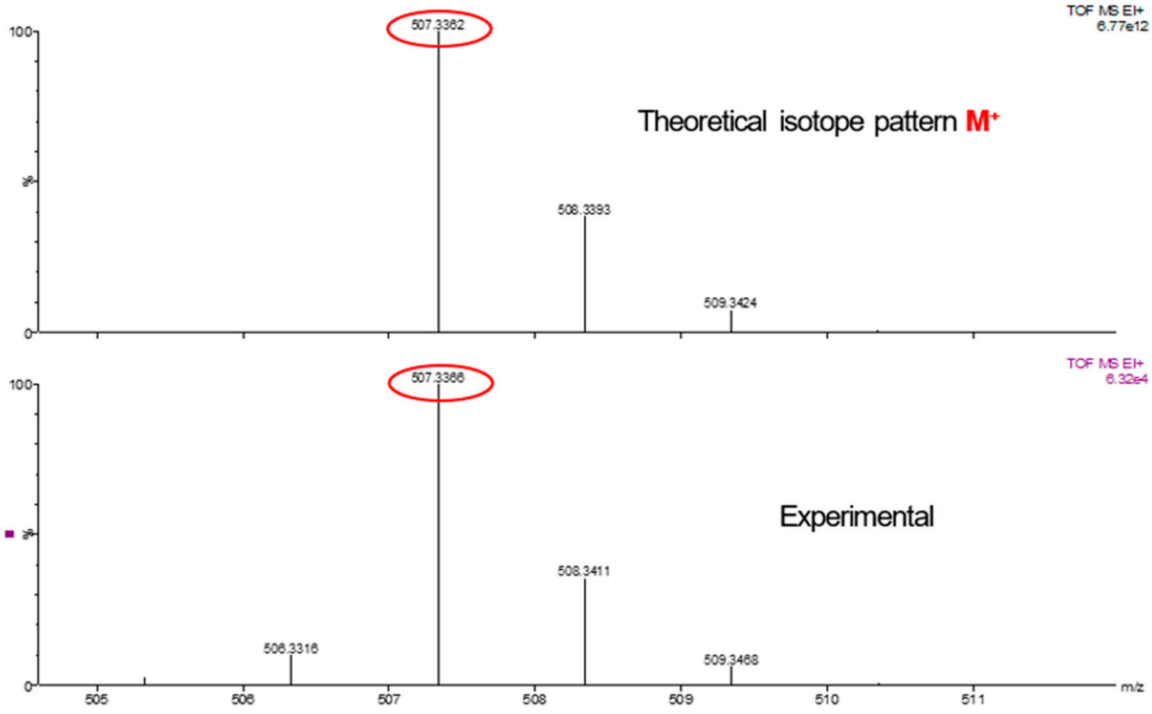

19 H nmr

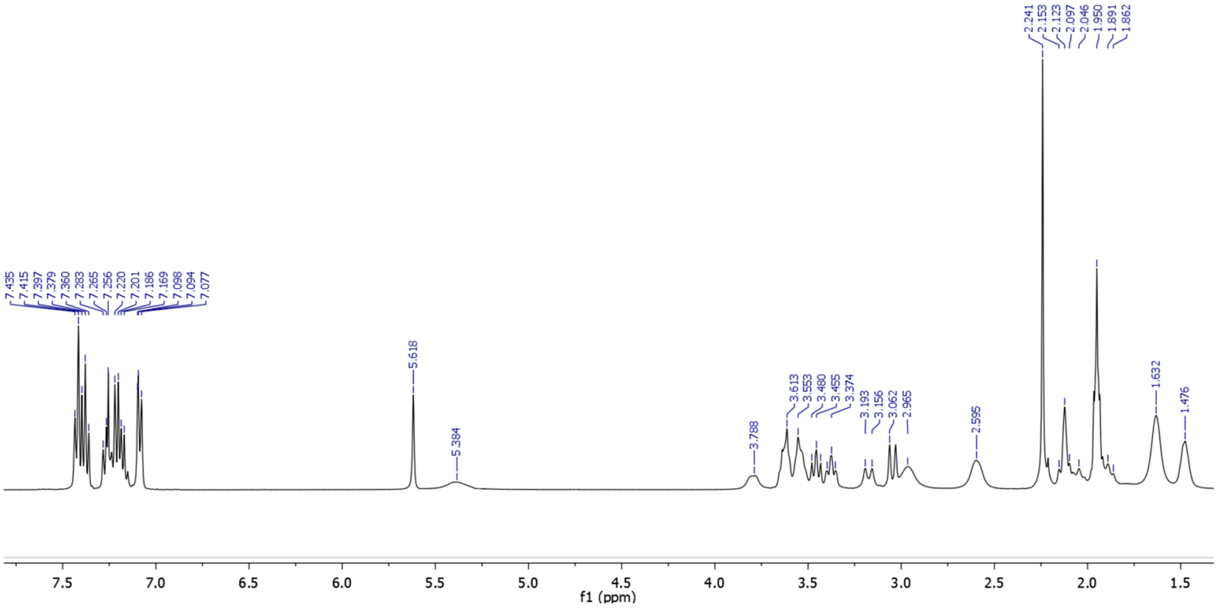

19 C nmr

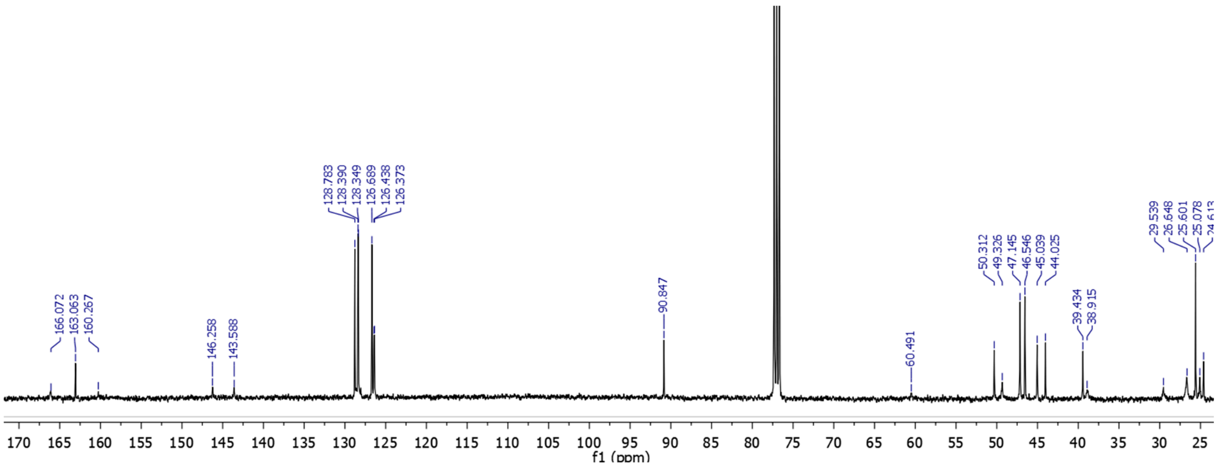

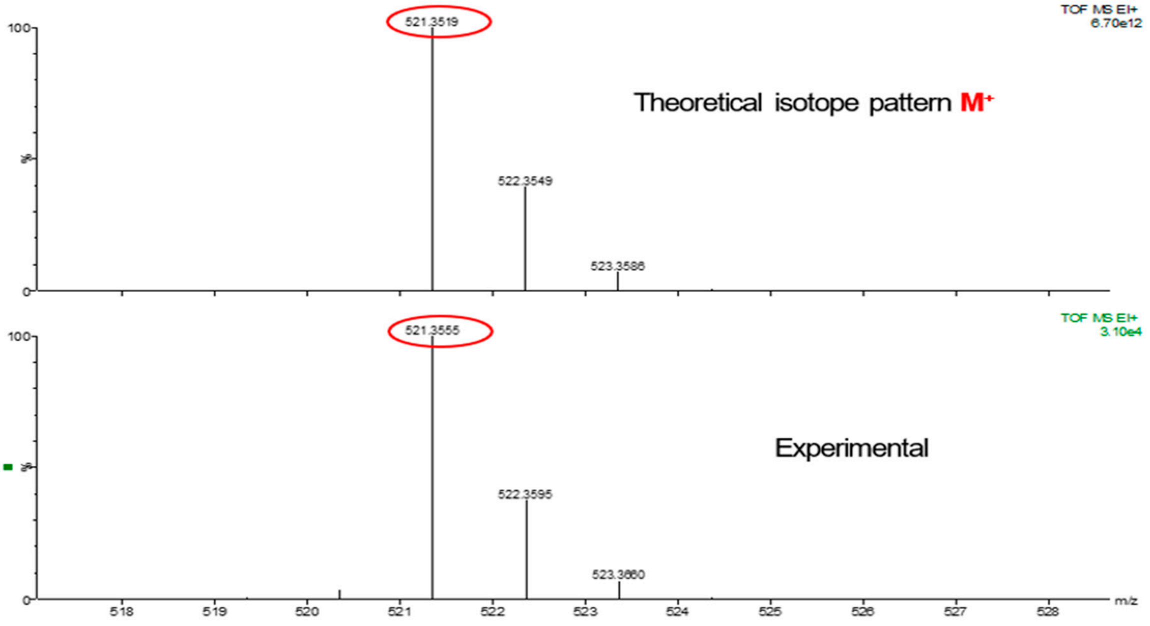

20 H nmr

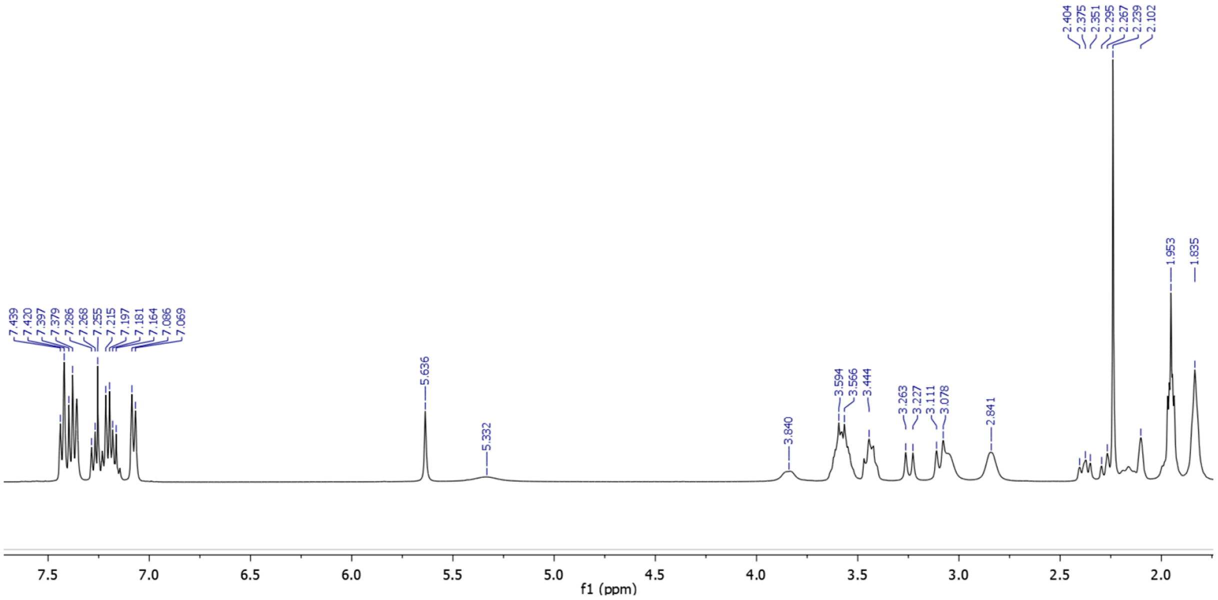

20 C nmr

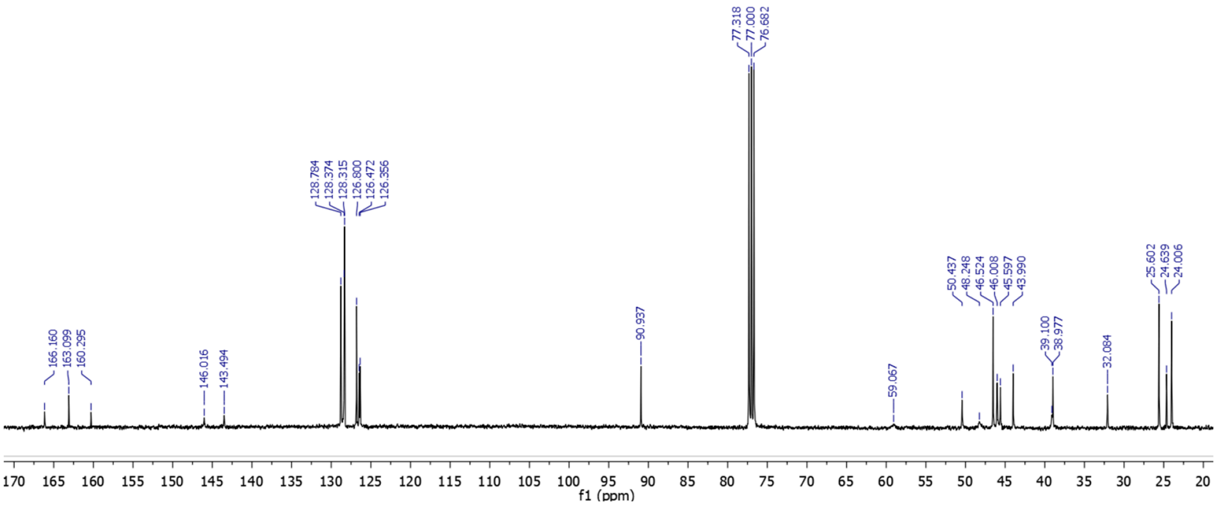

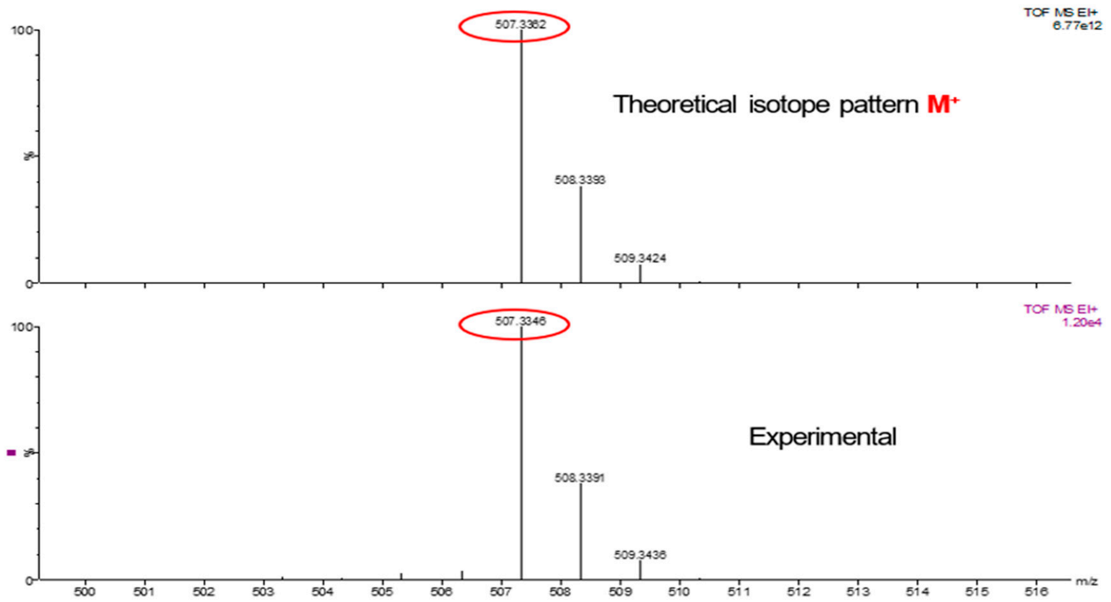

21 H nmr

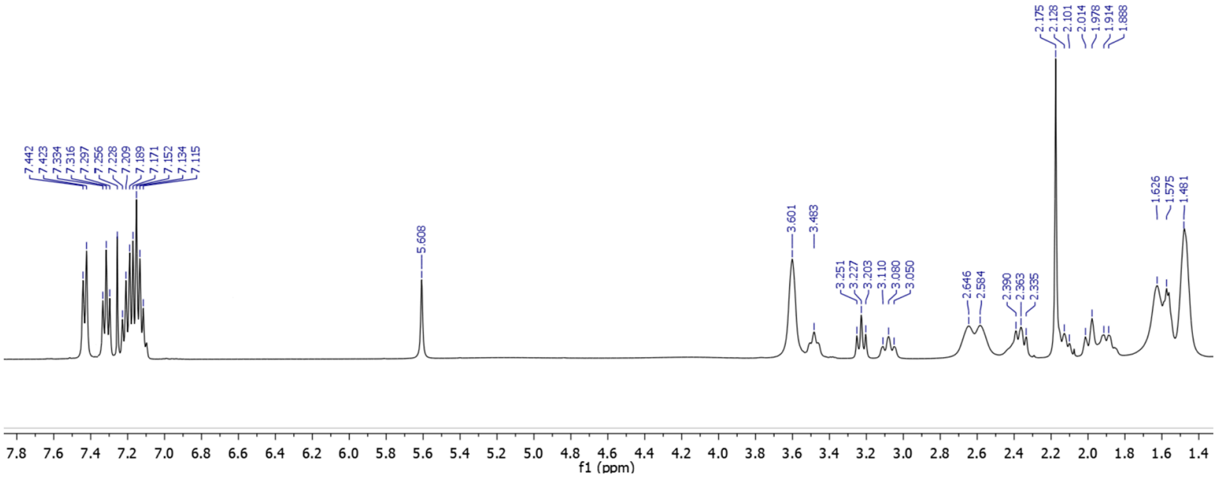

21 C nmr

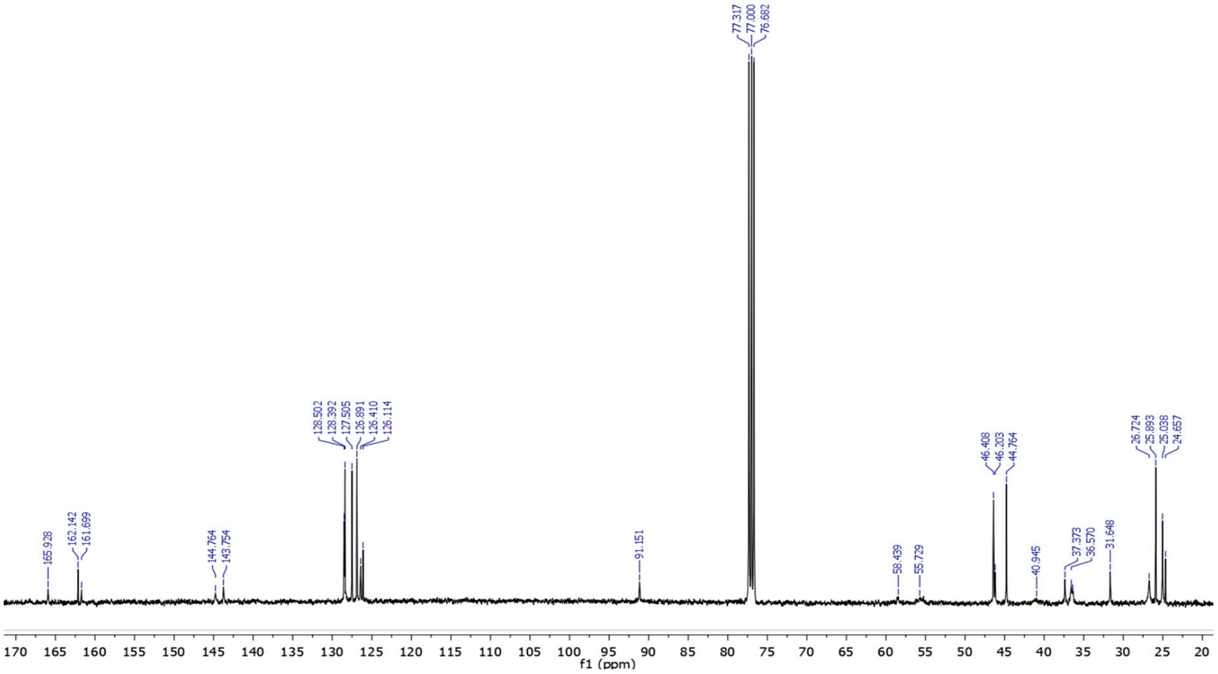

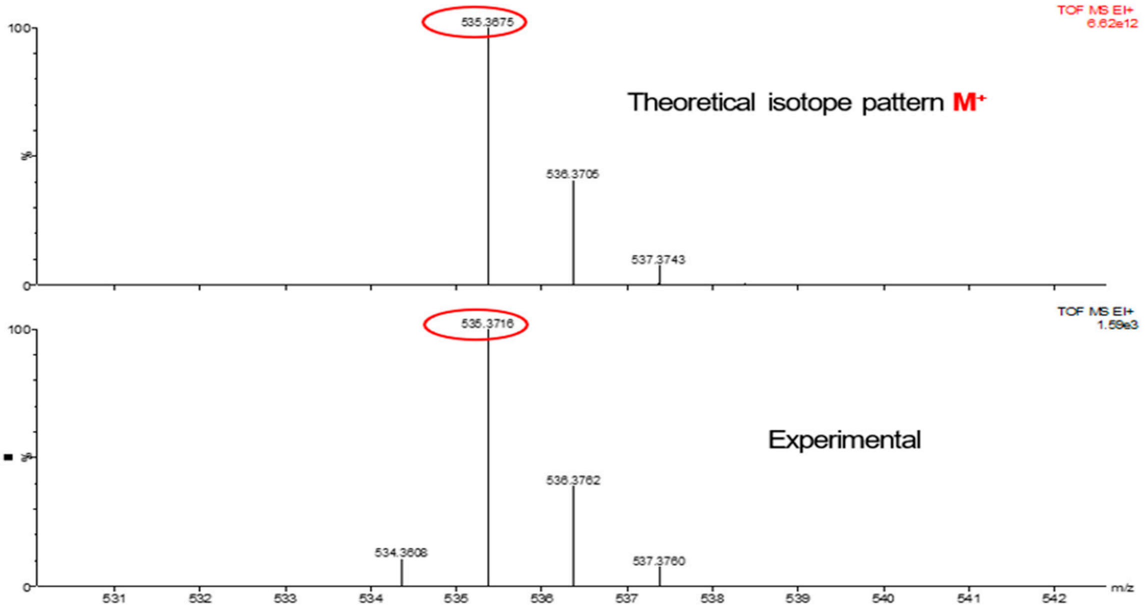

22 H nmr

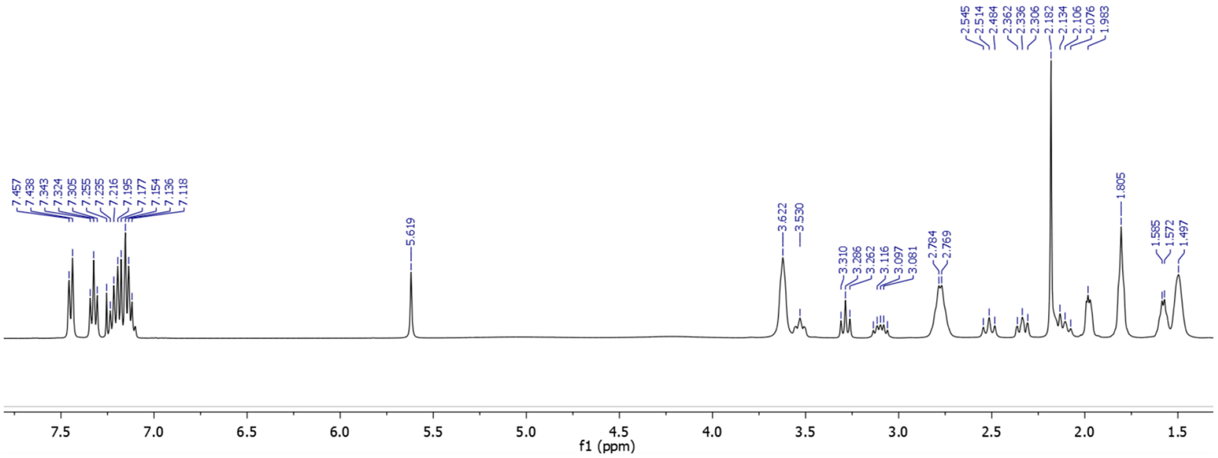

22 C nmr

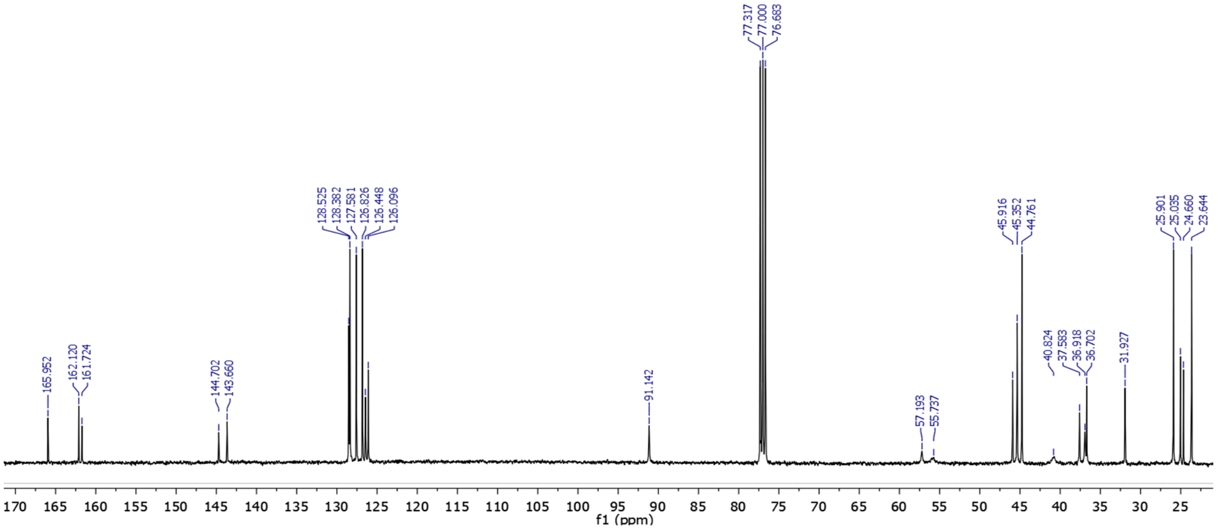

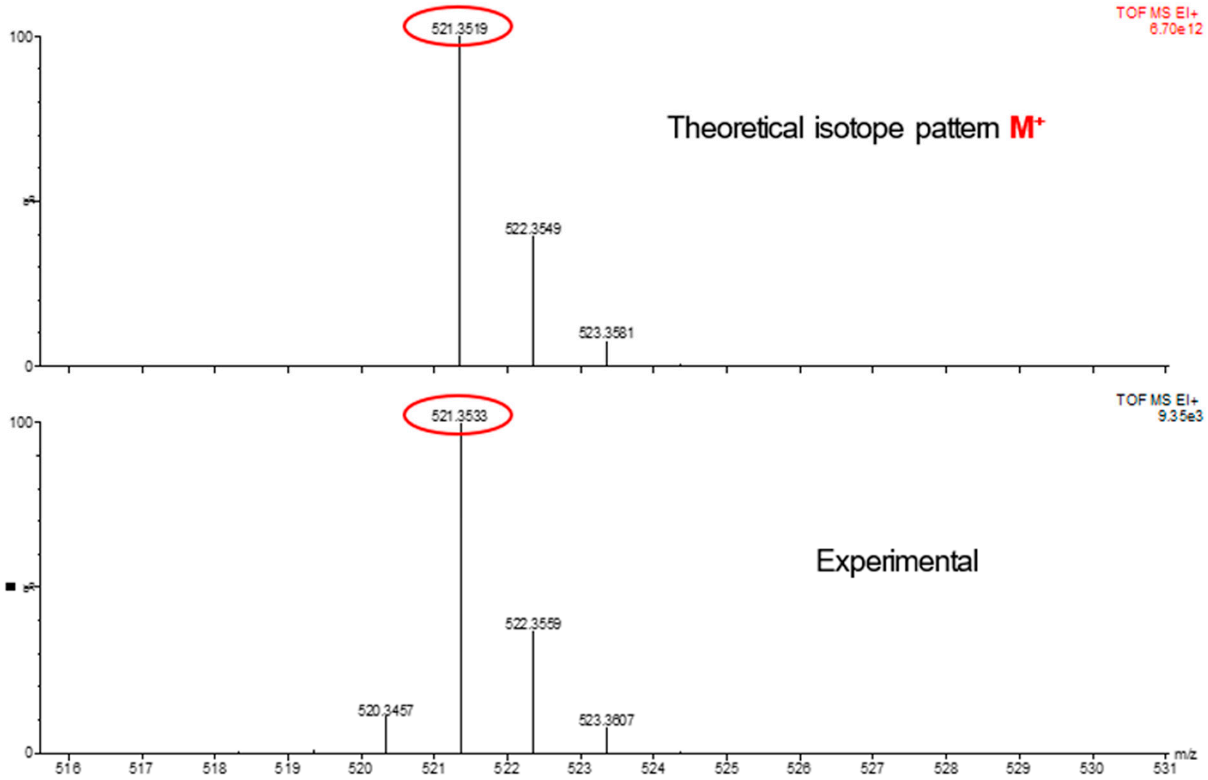

23 H nmr

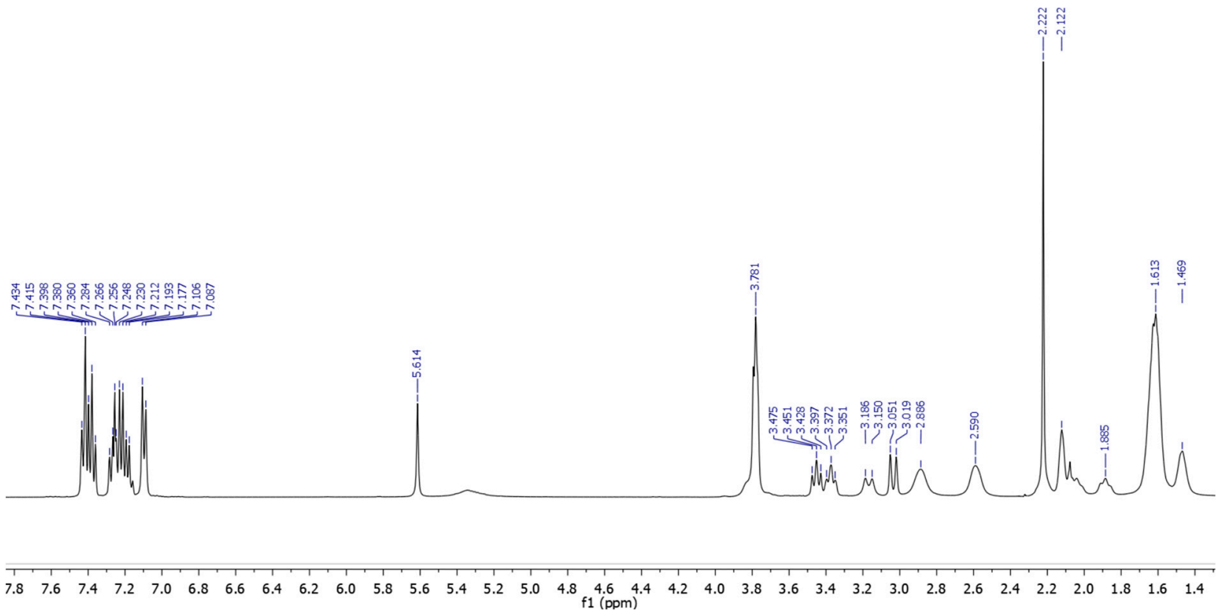

23 C nmr

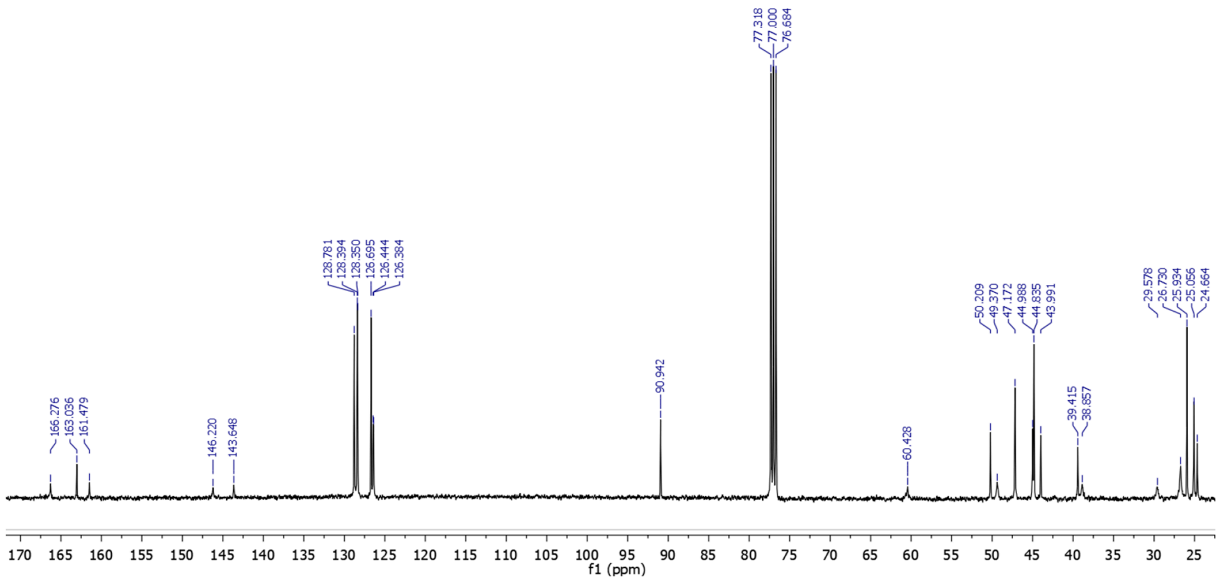

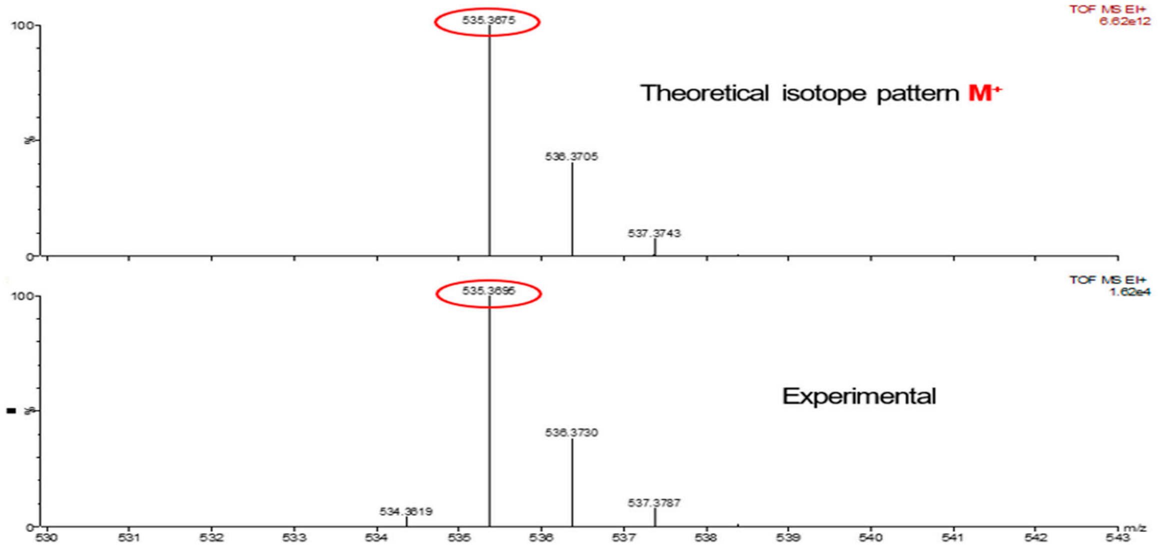

24 H nmr

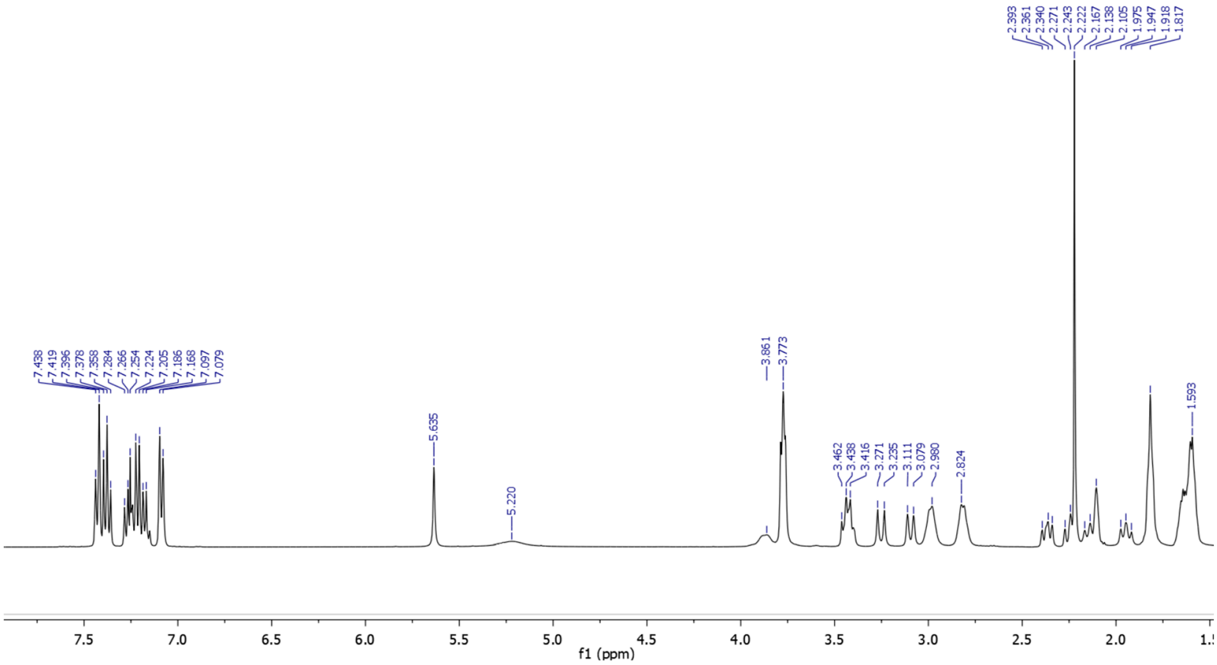

24 C nmr

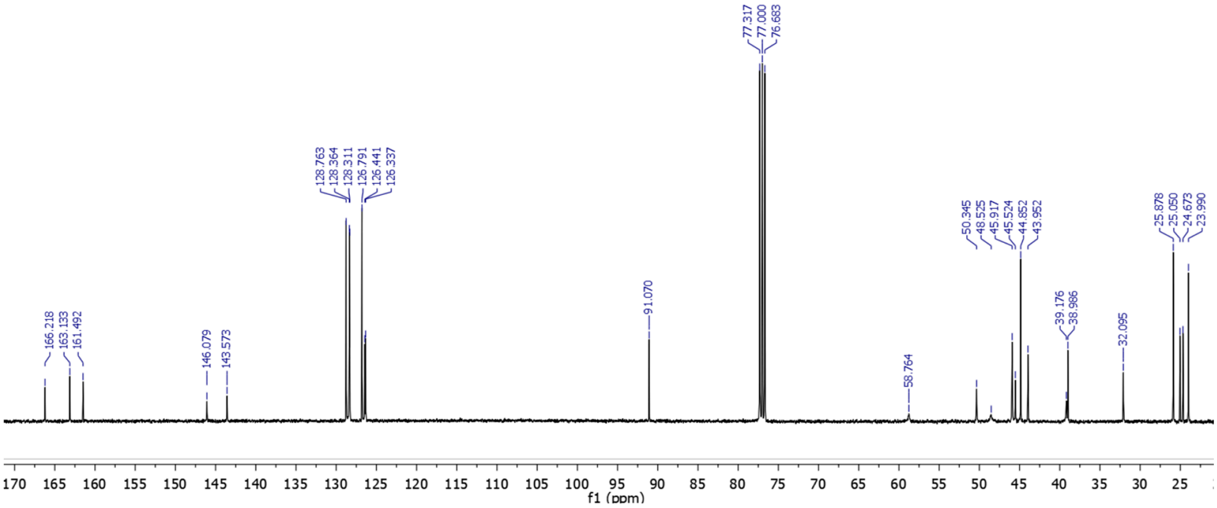

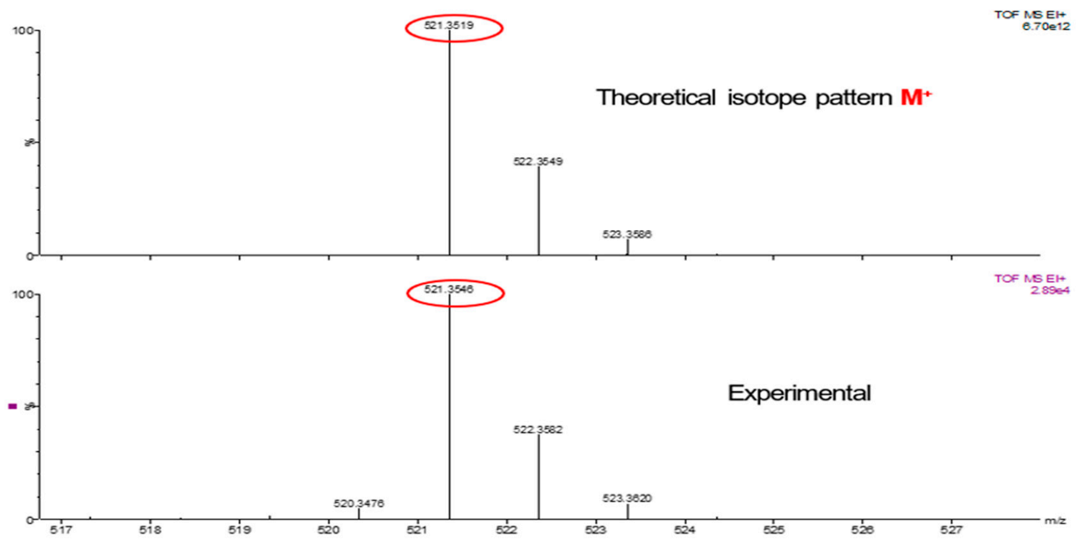

Synthesis route of compounds 1-4

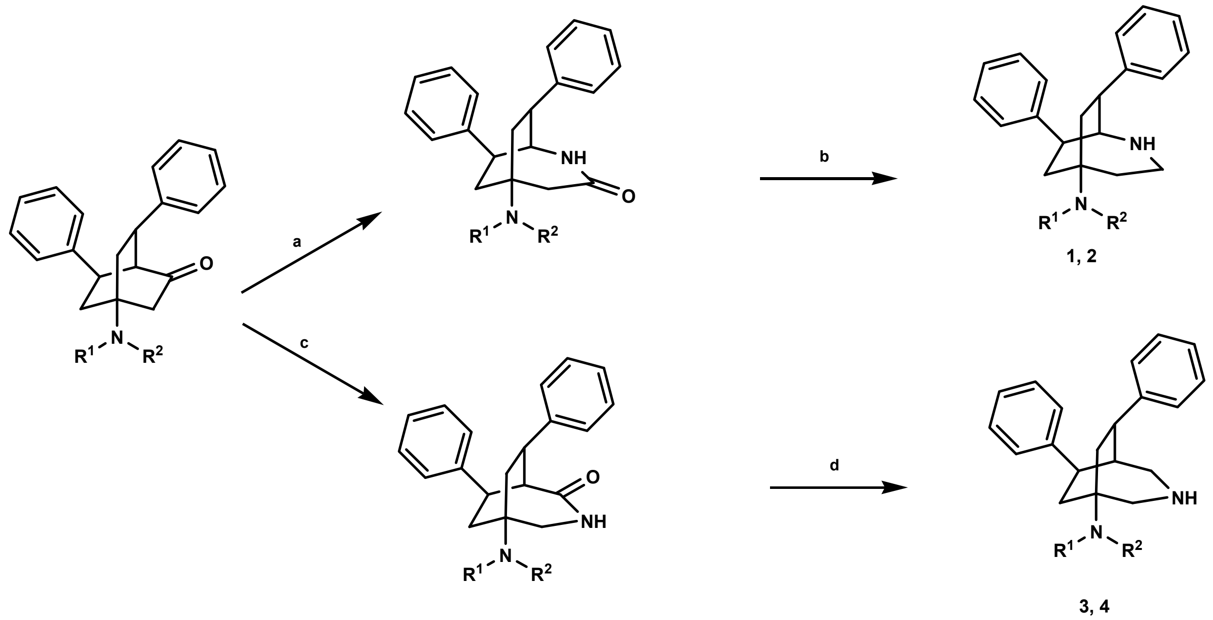

Compounds	R^1+R^2
**1, 3**	-$(CH_2)_5$-
**2, 4**	-$(CH_2)_4$-

Preparation of **1-4**. Reagents and conditions: (a) hydroxylamine-O-sulfonic acid, glacial acetic acid, 145°C, 16h; (b) $LiAlH_4$, diethyl ether, 55°C, 48h; (c) NaN_3, conc. H_2SO_4, 20°C; (d) $LiAlH_4$, diethyl ether, 55°C, 18h.
